# The Application of Flow Chemistry for the Synthesis of Alkyl Sodium Compounds and Their Transformations with Weinreb Amides and Carboxylic Acids

**DOI:** 10.1021/acs.orglett.4c02314

**Published:** 2024-08-14

**Authors:** Paula Knupe-Wolfgang, Bennett Mahn, Gerhard Hilt

**Affiliations:** Institute of Chemistry, Carl von Ossietzky University Oldenburg, Carl-von-Ossietzky-Straße 9-11, 26129 Oldenburg, Germany

## Abstract

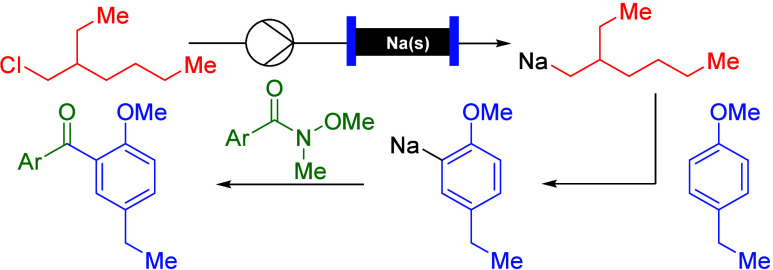

Herein, we describe the application of a flow system for the generation of a soluble organo sodium compound and the transformation of this primary nucleophile with various Weinreb amides for the synthesis of alkyl-aryl ketones. Thereafter, the generation of secondary sodium intermediates, such as benzylic sodium nucleophiles or *ortho*-metalated sodium nucleophiles from various carbon pre-nucleophiles, is described. These transformations generated more complex ketones, and in this investigation the key aspect was to identify factors for the chemoselective and regioselective C–H deprotonation of concurring sites within the starting material. Finally, the direct synthesis of ketones from carboxylic acids and the organo sodium compound is described, revealing interesting aspects regarding the nucleophilicity and basicity of the alkyl sodium reagent.

For the synthesis of ketones from carboxylic acid derivatives with carbon nucleophiles, the use of Weinreb amides is a prominent strategy to avoid the double nucleophilic attack on the carbonyl carbon for the formation of tertiary alcohols as side-product (Scheme 1a). As carbon nucleophiles, organo lithium compounds and Grignard reagents have been used on various occasions.^1^ The Weinreb amides were also recently used by Hevia for the synthesis of ketones using benzyl sodium reagents which were prepared in a batch-type fashion (Scheme 1b). Based on the instability of alkyl sodium reagents, a stabilizing triamine ligand had to be added.^2^ Some time ago, we explored the possibilities to generate a chiral cyclic Weinreb amide derivative (Scheme 1c) for the three-component synthesis of α-chiral ketones.^2,3^

**Scheme 1:**
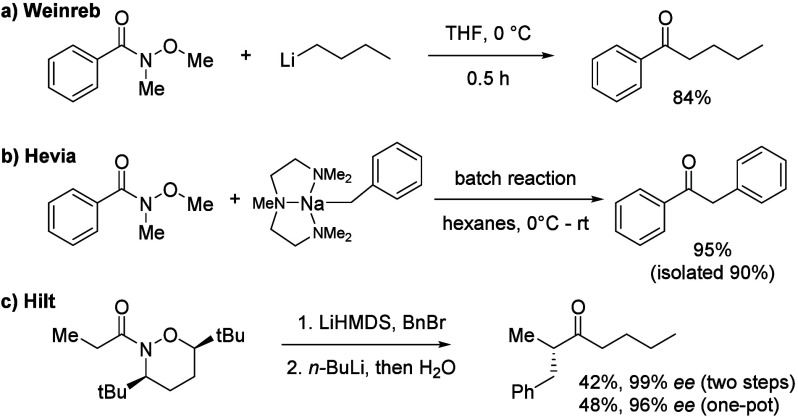
Transformation of Weinreb Amides with First-Row Metal Carbon Nucleophiles

In this reaction sequence, the α-alkylation of the amide was followed by the reaction with organo lithium compounds, such as *n*-BuLi or *s*-BuLi,^4^ to generate the desired ketone. However, in these reactions, up to 3.0 equiv of the alkyl lithium reagent had to be applied to achieve good conversions and good yields.

Therefore, we became interested in the use of organo sodium reagents, which are more reactive, but also quite unstable when prepared in a batch-type mode, unless stabilized by multidentate amine ligands as shown above by Hevia (Scheme 1b).^5^

Inspired by the ground-breaking work reported by Knochel for the synthesis of soluble organo sodium compounds from the alkyl chloride **1**, we also attempted the use of such organo sodium reagents, such as **2**, prepared in a flow system.^6^ The organo sodium reagent was then brought to reaction with Weinreb amides, such as **3a**, in n-hexane at room temperature for the formation of desired ketone **4a**. In this setup, we intended to explore some interesting aspects of this transformation. The outline of the transformation is given in Scheme 2.

**Scheme 2:**
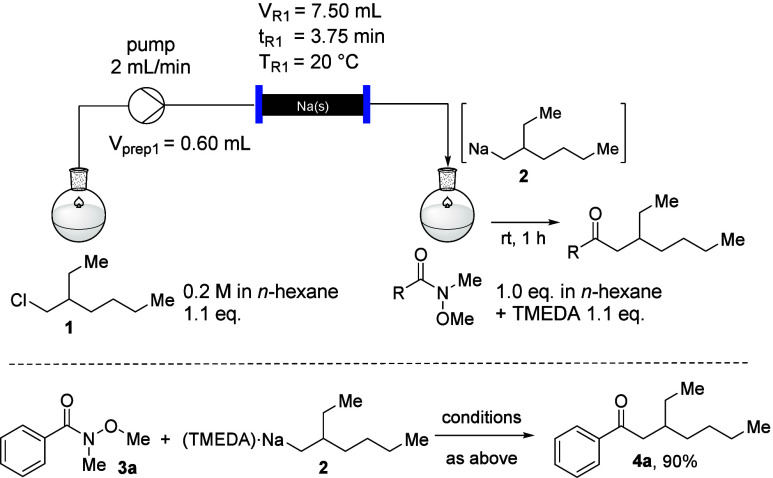
Outline of the Flow Chemistry for the Synthesis of Organo Sodium Reagents and Reaction with a Weinreb Amide

Based on the results reported by Knochel and Hevia,^5,6^ we applied the in-flow-generated organo sodium reagent **2** for the synthesis of the ketones, such as **4a**, from a simple Weinreb amide **3a** to confirm that our flow system is able to reproduce the reported results (Scheme 2). At first, we were concerned that the reproducibility of such transformations in a flow system could be problematic because numerous reaction parameters involved in the heterogeneous transformation, such as the particle size of the sodium, the flow rate, the concentration of **1**, the concentration of TMEDA (= *N*,*N*,*N′*,*N′*-tetramethylethylenediamine) for the solubilization and stabilization of the organo sodium reagent, and so on, have to be considered. In contrast to the report by Knochel, where a self-made sodium suspension was utilized, we obtained similar results with commercially available sodium dispersions for the synthesis of **4a**.

In the aftermath of this successful test reaction, which showed that our setup is in accordance with the literature, we decided to evaluate the performance of the organo sodium compound **2** in the reaction with a number of Weinreb amides, modifying the substituent R in these Weinreb amides of type **1** (Scheme 3). These transformations were performed to determine advantages or disadvantages compared to the Hevia batch-type reaction method (see Scheme 1b).^5^ The results of this investigation are summarized in Scheme 3.

**Scheme 3:**
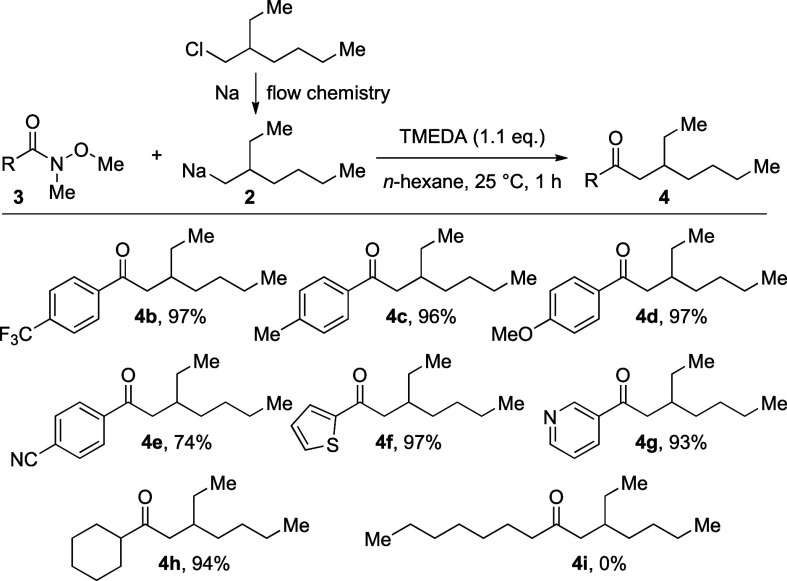
Transformation of Weinreb Amides with Organo Sodium Carbon Nucleophile **2**

For the synthesis of the ketones **4a**–**h**, 1.1 equiv of the alkyl sodium reagent was used from the flow system utilizing commercially available sodium particles, the alkyl chloride **1** in hexane at 20 °C.^7^ The addition to the Weinreb amide in hexane in the presence of 1.1 equiv of TMEDA was performed at 20 °C (generally, 1 h reaction time). The desired ketones of type **4** were obtained in good to excellent yields whereby electron-deficient aryl substituents (**4b**) as well as electron-neutral (**4a**/**4c**) and electron-rich aryls (**4d**) were accepted to afford the desired ketones in very good yields. The only exception was the 4-NC-C_6_H_4_-substituted Weinreb amide (**4e**), where also a nucleophilic attack of the alkyl sodium reagent **2** to the nitrile moiety led to the formation of side-products and diminished the amount of the desired product **4e**. However, this product was still isolated in a reasonably good yield of 74%. Also, of considerable interest are heteroarene-substituted Weinreb amides, such as those to afford the products **4f** and **4g**. The heteroatom of the aromatic substituents could have led to an *ortho*-directed deprotonation of the arene ring and undesired side-products or diminished yields by reprotonation during the workup. However, the yields of **4f** and **4g** of 97% and 93%, respectively, illustrate that such side-reactions did not occur, but that probably the heteroatom is helpful to direct the nucleophilic attack to the adjacent carbonyl group of these Weinreb amides. When aliphatic Weinreb amides were used, an interesting observation was made. While the Weinreb amide derived from cyclohexyl carboxylic acid afforded the desired ketone **4h** in a very good yield of 94%, the corresponding ketone **4i**, derived from octanoic acid, could not be obtained under these conditions. This sharp contrast in reactivity can be rationalized by the basicity of the alkyl sodium reagent **2**, which led to deprotonation of the less-hindered starting material **3i**, and after aqueous workup the starting material was recovered (R = *n*-heptyl) unchanged. Accordingly, only a small limitation of the substituent R of the aromatic Weinreb amide was detectable (R = 4-NC-C_6_H_4_, for the product **4e**) in this series of experiments utilizing a flow system for the generation of the sensitive primary organo sodium nucleophile **2**. However, it should be clear that other reactive substituents on the aryl moiety, such as an aryl iodide, or other carbonyl groups, such as an aldehyde, will cause side reactions with the organo sodium reagent **2**.

In light of our previous investigation applying cyclic Weinreb amide-type reagents,^2^ we were very pleased that these transformations gave excellent yields with 1.1 equiv of the primary alkyl sodium reagent **2** in most reactions utilizing aromatic and sterically hindered alkyl Weinreb amides.

Therefore, we directed our attention to the synthesis of secondary sodium nucleophiles, which are generated in the reaction of **2** with appropriate pre-nucleophiles of type **5** (Scheme 4). For this purpose, the primary alkyl sodium reagent **2** was added to the pre-nucleophile (1.5 equiv of H-X-R) in hexane at 20 °C to generate the secondary nucleophile **6***in situ* in the presence of TMEDA (1.1 equiv). After a 15 min reaction time, the Weinreb amide (1.0 equiv) was added and the mixture was stirred for an additional hour at ambient temperature before workup to generate the desired ketone of type **7**.

**Scheme 4:**
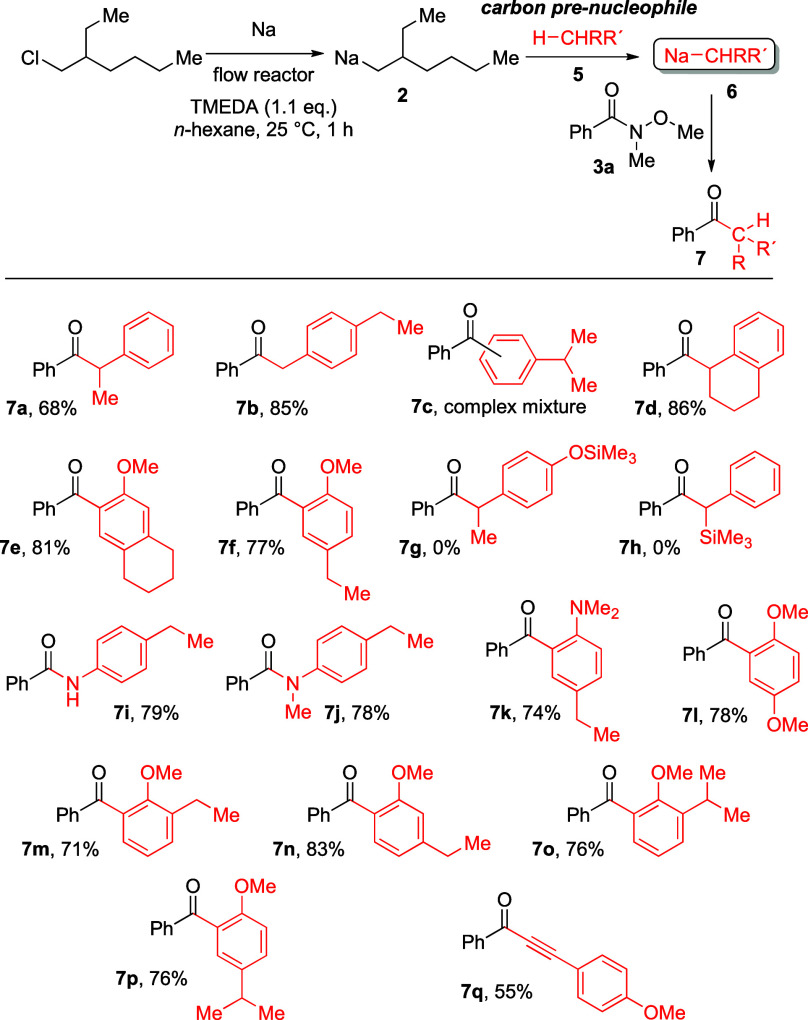
Generation of Secondary Sodium Reagents and Their Reaction with the Weinreb Amide **3a**

For this purpose, at first, ethylbenzene was applied as pre-nucleophile to ensure that our flow system generates similar results to those described by Knochel for the synthesis of **7a**. Thereafter, an arene derivative (4-ethyltoluene) was utilized to reproduce the results described by Hevia.^5^

In this set of experiments, we were interested to determine the factors under which a deprotonation of a substituted arene takes place, particularly when alternative sites for the deprotonation are present in the carbon pre-nucelophile **5**. The results of these reactions are summarized in Scheme 4.

The transformation of ethylbenzene as pre-nucleophile with **2** and the reaction with the Weinreb amide **3a** led to the formation of **7a** in acceptable yield (68%) comparable with the yield reported by Hevia for the batch reaction method (75%). When 4-ethyltoluene was used as carbon pre-nucleophile (**5**), two different benzylic sites could have been deprotonated by **2**: either the methyl group or the benzylic position of the ethyl group. The deprotonation occurred selectively at the methyl group for the formation of **7b** in good, 85% yield. The isopropyl group in the carbon pre-nucleophile isopropylbenzene could not be deprotonated selectively. It seems that in this case the arene ring was deprotonated and led to a mixture of regioisomers, which could not be assigned by ^1^H and ^13^C NMR. However, tetrahydronaphthalene led to a good yield of **7d** (86%) upon deprotonation of the benzylic position, whereas the corresponding methoxy derivative showed a different chemoselectivity. In this case, a directed *ortho*-deprotonation was observed and **7e** was formed chemo- and regioselectively and was isolated in 81% yield. A similar behavior was observed for the synthesis of **7f** where the *ortho*-directed deprotonation of the arene ring overrules the deprotonation in the benzylic position. Accordingly, the product **7f** was isolated in a good 77% yield. In the next experiment, we attempted to invert the chemoselective deprotonation in favor of the benzylic position by introducing trimethylsilyl protection of the phenolic oxygen to generate the desired product **7g**. To our surprise, **7g** was not formed at all, and we have no explanation for this behavior at the present time. Also of interest is the transformation with benzyltrimethylsilane for the formation of **7h**. In this case, deprotonation should deliver a benzylic anion, which is additionally stabilized by the α-silyl effect. Again, the desired product **7h**, which would have been an excellent precursor for follow-up reaction toward the Peterson olefination, was not formed. Thereafter, we turned our attention toward aniline derivatives with an ethyl-substituent in the 4-position. Initially, we expected in the case of the H_2_N and the MeHN derivative also an *ortho*-directed deprotonation as in the case of the Me_2_N derivative. However, the first two derivatives gave chemoselectively the corresponding amides **7i** and **7j** in good yields upon NH-deprotonation, whereas the *ortho*-deprotonation product **7k** was formed from the Me_2_N derivative in a reasonably good isolated yield of 74%. Noteworthy, the alternative deprotonation of the ethyl group in benzylic position was not observed in the three reactions of the aniline derivatives. It is therefore not surprising that product **7l** is formed as the sole product, as alternative sites for deprotonation are not present. The yield of 78% for **7l** is in line with the other examples reported thus far for the *ortho*-directed deprotonation of methoxy and dimethylaniline derivatives and illustrates that the chemoselectivity is strongly influenced by coordinating heteroatoms overruling the deprotonation of an alkyl chain in the benzylic position if the alkyl chain is not adjacent to the heteroatom-bearing group. In the next experiment, we intended to see if the sodium base **2** would be directed by the methoxy group toward the deprotonation of the adjacent arene C–H bond or toward the deprotonation of the adjacent benzylic CH_2_ group in 2-ethylanisole. The product **7m** was isolated in 71% yield as sole product, indicating that the *ortho*-directing effect prefers the less sterically hindered aromatic C–H bond. For 3-ethylanisole, the methoxy group and the ethyl substituent are in a 1,3-relation and a regioselective C–H deprotonation of **2** at the less hindered 6-position is observed to result in the formation of **7n** in good, 83% yield. Accordingly, 2- and 4-isopropylanisole led to the formation of *ortho*-directed deprotonation/benzoylation products **7o** and **7p** in 76% yield each. When a terminal alkyne was used, the *ortho*-directing effect of the methoxy group on the arene ring is out-ruled by the CH acidity of the terminal alkyne and the product **7q** is formed chemoselectively and was isolated in 55%.

Instead of the use of Weinreb amides, a direct route to ketones is realized when carbocyclic acids are directly converted with alkyl lithium reagents.^8^ The dilithium-dialkoxide intermediates are stable under the reaction conditions and are converted to the corresponding ketones by aqueous workup. Therefore, we also investigated the alkyl sodium reagent **2** to access ketones of type **9** from carboxylic acids **8** (Scheme 5). For this purpose, the reaction conditions had to be optimized (see Supporting Information S27 and S28), and the best results were obtained when the carboxylic acid was suspended in THF at room temperature in the presence of LiCl (6.0 equiv) under sonication for 30 min and reaction overnight at room temperature to obtain the desired ketones of type **9**. The results for the transformations of aromatic/heteroaromatic and cyclohexyl carboxylic acid are summarized in Scheme 5.

**Scheme 5:**
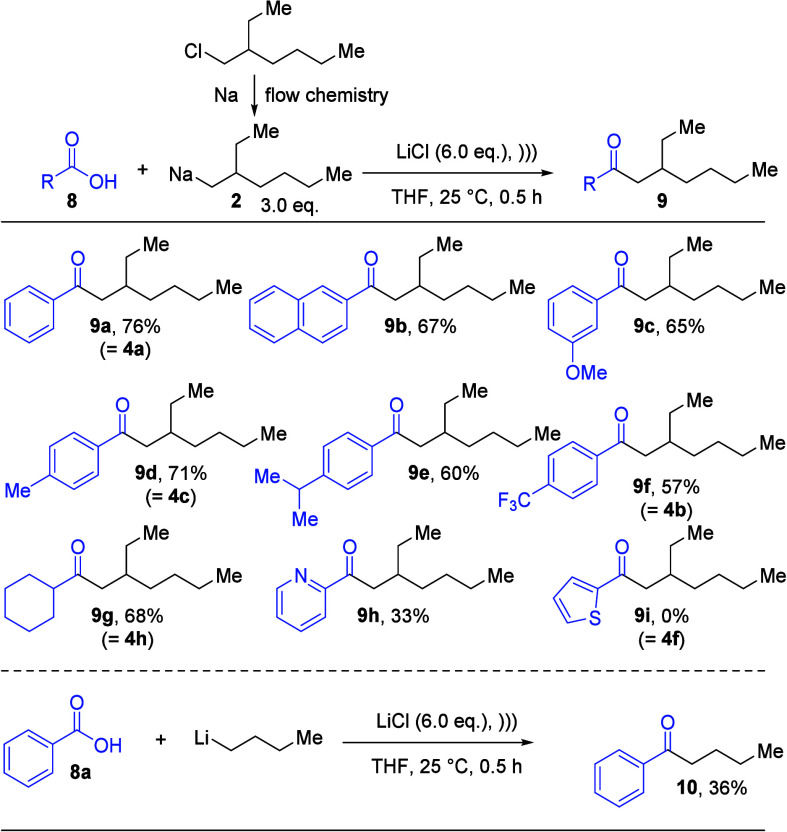
Synthesis of Ketones (**9**) from the Alkyl Sodium Reagent **2** and Carboxylic Acids (**8**)

As can be seen in Scheme 5, benzoic acid and 2-naphthoic acid gave desired ketones **9a** and **9b** in acceptable yields of 76% and 67%, respectively. Other substituted carboxylic acids could also be applied to generate the alkoxy-substituted aromatic ketone **9c**, the alkyl-substituted aryl ketones **9d** and **9e**, and the trifluoromethyl-substituted aromatic ketone **9f** in yields ranging from 57% to 71%. As an aliphatic carboxylic acid, we chose cyclohexylcarboxylic acid as the substrate, and the desired ketone **9g** was isolated in 68% yield. Finally, also two heterocyclic carboxylic acids were tested, and in this case, the picolinic acid reacted sluggishly to the ketone and only 33% of the desired ketone **9h** was isolated. Moreover, ketone **9i** derived from 2-thienyl carboxylic acid could not be obtained. We assume that, in this case, the carboxylate led to an α-directed deprotonation of the thienyl moiety by the alkyl sodium reagent **2**, and after aqueous workup the starting material is reformed. This undesired side-reaction could also account for the reduced yield for the ketone **9i**, and in these cases, the Weinreb amide route is of significant advantage. However, the methoxy group embedded in the starting material **8c** had a negligible impact on the yield for the synthesis of **9c**. Therefore, the alkyl sodium reagent **2** seems to have a hitherto unexplored potential when concurring reaction pathways are offered.

As a control experiment for the enhanced reactivity of **2**, we also reacted benzoic acid (**8a**) under the same reaction conditions as were used for the alkyl sodium reagent **2** with *n*-butyl lithium, and the ketone **10** could only be isolated in a comparable low yield of 36%.

Accordingly, we were able to convert the carboxylic acids directly into ketones with the alkyl sodium reagent **2**, while heterocyclic carboxylic acids imposed some limitations on the direct methodology.

In summary, we accomplished the flow generation of the soluble alkyl sodium reagent **2** for the synthesis of ketones from Weinreb amides in high yields. In comparison with the batch-reaction reported by Hevia, similar good results could be obtained with a simpler amine as a stabilizing agent (TMEDA). Also, the alkyl sodium reagent **2** was used for the generation of secondary alkyl sodium reagents *in situ*, which led to the ketones of type **7** when reacted with Weinreb amide **3a** as test substrate. In this part of the investigation, very interesting trends for the reaction of **2** with all-carbon pre-electrophiles were observed. The presence of heteroatom substituents leads either to α-directed deprotonation (for −OMe and −NMe_2_) or to functional group deprotonation (for −NH_2_ and −NHMe). The deprotonation of a terminal alkyne overruled the α-directing deprotonation and led to an ynone product. Finally, we also investigated the direct approach to ketones of type **9** from the corresponding carboxylic acids. Thereby, the formation of a carboxylic acid chloride and conversion into a Weinreb amide can be avoided. For a number of carboxylic acids this short cut is an attractive alternative; however, for heteroaromatic systems, such as **9h** and **9i**, this route is of low efficiency compared to the Weinreb route. Therefore, we look forward to having a way toward a playground where Weinreb amides or carboxylic acids can be converted to ketones either with the reagent **2** (or similar alkyl sodium reagents) or with secondary nucleophiles generated from **2***in situ*.

## Data Availability

The data underlying this study are available in the published article and its Supporting Information.

## Supporting Information Available

Experimental procedures; spectroscopic characterization data (PDF)
