# Supplementary material for: The Application of Flow Chemistry for the Synthesis of Alkyl Sodium Compounds and Their Transformations with Weinreb Amides and Carboxylic Acids
Source: Org Lett. 2024 Aug 14;26(33):6972–6. doi: 10.1021/acs.orglett.4c02314 (PMC11348415; doi:10.1021/acs.orglett.4c02314)
Supplement: Supplementary file 1 — ol4c02314_si_001.pdf [file ol4c02314_si_001.pdf]

SUPPORTING INFORMATION

The Application of Flow Chemistry for the Synthesis of Alkyl Sodium Compounds and their Transformations with Weinreb Amides and Carboxylic Acids

Paula Knupe-Wolfgang, Bennett Mahn and Gerhard Hilt*

Institut für Chemie, Carl von Ossietzky Universität Oldenburg, Carl-von-Ossietzky Str. 9-11, 26129 Oldenburg, Germany

gerhard.hilt@uni-oldenburg.de

Table of content

1. General Information S2

2. Educts Synthesis S3

3. Synthesis of Ketones from Weinreb Amides with 2-(Ethylhexyl)sodium S7

4. Preparation of Ketones with Secondary Sodium Compound S13

5. Preparation of Ketones from Benzoic Acid Derivatives S27

 5.1 Optimization of the Reaction Conditions S27

 5.2 Synthesized Ketones from Benzoic Acid Derivatives S29

6. Reaction under the Optimized Conditions of Benzoic Acid with *n*-Butyllithium S33

7. ^{1}H and ^{13}C NMR Spectra of the Substrate and all Products S35

8. References S76

1. General Information

All solvents were purchased commercially and were distilled under reduced pressure before use. The solvents were dried over 3 Å molecular sieve. The anhydrous *n*-hexane used was purchased commercially from TCI chemicals. Unless otherwise described, reactants and reagents were purchased commercially and used without further purification. When the reaction was carried out under absence of water and air, the experiments were carried out in oven-dried glassware using SCHLENK techniques under nitrogen atmosphere. Flash chromatography was carried out using Macherey-Nagel silica gel 60 (0.040-0.063 mm). TLC was performed on aluminum plates coated with SiO_2 F_{254} with fluorescence indicator. For the detection of the signals ultraviolet light (λ = 254 nm) or heating after the plate has been dipped into a $KMnO_4$-solution. All unknown products were identified by ^{1}H NMR, ^{13}C{^{1}H} NMR, IR and HRMS. A Vapourtec E-series Integrated Flow Chemistry System with 3rd Pump Kit, Organometallic Kit, Collection Valve Kit and Cryogenic Reaction Kit was used.

NMR spectroscopy

^{1}H, ^{13}C and ^{19}F NMR spectra were recorded on Bruker Avance 300 / 500 instruments. Chemical shifts are reported in parts per million (ppm). The spectra are referenced to the residual solvent peak of $CDCl_3$ or CD_2Cl_2. In the ^{1}H NMR spectra this corresponds with the singlet of the solvent signal of $CDCl_3$ at δ = 7.26 ppm and CD_2Cl_2 at δ = 5.32 ppm. The ^{13}C NMR spectra were referenced to the central line of the triplet of $CDCl_3$ at δ = 77.16 ppm or of the quintet of CD_2Cl_2 at δ = 53.84 ppm. The stated form of the signal describes the appearance of the signal and not the theoretically expected form.

IR Spectroscopy

IR spectra were obtained with Shimadzu IR Spirit T spectrometer equipped with diamond ATR units.

Gas chromatography (GC)

A SHIMADZU GC-2010 Plus series gas chromatograph with flame ionization detector (FID) and a MACHERY NAGEL Optima 5 HAT column (15 m) was used to record the gas chromatograms.

HRMS

MS and HRMS spectra were obtained with Waters Q-TOF Premier (ESI, positive mode) or the Thermo Scientific DFS (EI, 70 eV) spectrometers.

2. Educt Synthesis

3-(Chloromethyl)heptane (1)

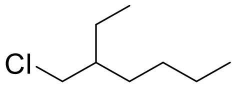

In a 500 mL three-neck flask equipped with a reflux condenser, 2-ethylhexan-1-ol (78.5 mL, 0.50 mol, 1.0 eq.) and pyridine (23 mL) were added and stirred at 0 °C. Thionyl chloride (47, 61.7 mL, 0.85 mol, 1.7 eq.) was then added over one hour. The reaction solution was stirred overnight at a temperature of 55 °C. Water (100 mL) was added to the reaction solution at 0 °C. The aqueous phase was extracted with ethyl acetate (3 x 100 mL) and the combined organic phases were dried over magnesium sulfate and filtered. The solvent was distilled off under reduced pressure and the crude product was isolated by column chromatography (SiO_2, *iso*-hexane: ethyl acetate = 99.5:0.5) to obtain 3-(chloromethyl)heptane (66.3 g, 0.45 mol, 90%) as a colorless oil.[1]

^{1}H NMR (300 MHz, $CDCl_3$): δ = 3.56-3.45 (m, 2H), 1.63-1.55 (m, 1H), 1.50-1.22 (m, 8H), 0.92-0.87 (m, 6H) ppm.

^{13}C{^{1}H} NMR (125 MHz, $CDCl_3$): δ = 48.5, 41.6, 31.1, 29.0, 24.3, 23.0, 14.2, 11.0 ppm.

The analytical data are in accordance with the literature.[1]

GP1: General procedure for the synthesis of the Weinreb amides[2]

In an oven-dried 25 ml Schlenk flask, *N,O*-dimethylhydroxylamine hydrochloride (1.0 eq.) was charged in anhydrous dichloromethane at 0 °C under a nitrogen atmosphere. Triethylamine (2.1 eq.) was added, whereby the temperature should not rise above 10 °C. As soon as the reaction solution had cooled back down to 0 °C, the corresponding acid chloride (1.0 eq.) was added and allowed to stir for the respective reaction time. Water was then added and the phases separated. The organic phase was dried over magnesium sulfate, filtered and the solvent removed under reduced pressure so that the product could be obtained.

***N*-Methoxy-*N*-methyl-4-trifluoromethylbenzamide (3b)**

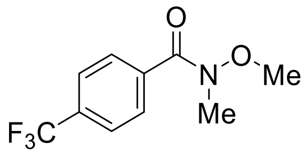

According to **GP 1**, *N,O*-dimethylhydroxylamine hydrochloride (732 mg, 7.5 mmol, 1.0 eq.) was dissolved in dichloromethane under a nitrogen atmosphere and triethylamine (1.59 g, 15.8 mmol, 2.1 eq.) and 4-(trifluoromethyl)benzoyl chloride (1.56 g, 7.5 mmol, 1.0 eq.) were added and stirred for 24 hours. After work-up as described, the product (1.64 g, 7.03 mmol, 94%) was obtained as a yellow liquid.

^{1}H NMR (300 MHz, $CDCl_3$): δ = 7.97-7.97 (m, 2H), 7.68-7.65 (m, 2H), 3.52 (s, 3H), 3.37 (s, 3H) ppm.

^{13}C{^{1}H} NMR (125 MHz, $CDCl_3$): δ = 168.5, 137.6, 132.2 (q, *J* = 32.6 Hz), 128.6, 125.0 (q, *J* = 4.1 Hz), 123.7 (q, *J* = 272.4 Hz), 61.2, 33.3 ppm.

The analytical data are in accordance with the literature.[3]

***N*-Methoxy-*N*,4-dimethylbenzamide (3c)**

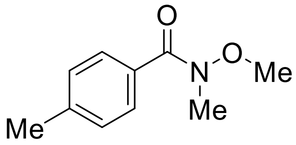

According to **GP 1**, *N,O*-dimethylhydroxylamine hydrochloride (732 mg, 7.5 mmol, 1.0 eq.) was dissolved in dichloromethane under a nitrogen atmosphere, triethylamine (1.59 g, 15.8 mmol, 2.1 eq.) and 4-methylbenzoic acid chloride (1.16 g, 7.5 mmol, 1.0 eq.) were added and stirred for 24 hours. After work-up as described, the product (1.30 g, 7.25 mmol, 97%) was obtained as a yellow liquid.

^{1}H NMR (300 MHz, $CDCl_3$): δ = 7.58 (d, *J* = 8.1 Hz, 2H), 7.19 (d, *J* = 7.9 Hz, 2H), 3.54 (s, 3H), 3.33 (s, 3H), 2.37 (s, 3H) ppm.

^{13}C{^{1}H} NMR (125 MHz, $CDCl_3$): δ = 168.9, 140.7, 130.9, 128.5, 128.1, 60.7, 33.7, 21.3 ppm.

The analytical data are in accordance with the literature.[4]

4,*N*-Dimethoxy-*N*-methylbenzamide (3d)

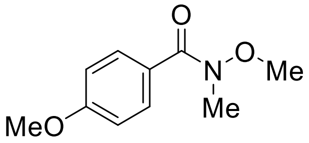

According to **GP 1**, *N,O*-dimethylhydroxylamine hydrochloride (732 mg, 7.5 mmol, 1.0 eq.) was dissolved in dichloromethane under a nitrogen atmosphere and triethylamine (1.59 g, 15.8 mmol, 2.1 eq.) and 4-methoxybenzoic acid chloride (1.28 g, 7.5 mmol, 1.0 eq.) were added and stirred for 24 hours. After work-up as described, the product (1.19 g, 8.1 mmol, 81%) was obtained as a yellow liquid.

^{1}H NMR (300 MHz, $CDCl_3$): δ = 7.71 (d, *J* = 8.9 Hz, 2H), 6.88 (d, *J* = 8.9 Hz, 2H), 3.82 (s, 3H), 3.54 (s, 3H), 3.33 (s, 3H) ppm.

^{13}C{^{1}H} NMR (125 MHz, $CDCl_3$): δ = 169.4, 161.6, 130.6, 126.0, 113.3, 61.0, 55.4, 34.0 ppm.

The analytical data are in accordance with the literature.[3]

***N*-Methoxy-*N*-methylthiophene-2-carboxamid (3f)**

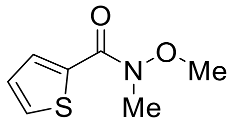

According to **GP 1**, *N,O*-dimethylhydroxylamine hydrochloride (732 mg, 7.5 mmol, 1.0 eq.) was dissolved in dichloromethane under a nitrogen atmosphere and triethylamine (1.59 g, 15.8 mmol, 2.1 eq.) and thiophene-2-carbonyl chloride (1.10 g, 7.5 mmol, 1.0 eq.) were added and stirred for 24 hours. After work-up as described, the product (1.20 g, 6.98 mmol, 93%) was obtained as a yellow liquid.

^{1}H NMR (300 MHz, $CDCl_3$): δ = 7.96 (d, *J* = 4.8 Hz, 1H), 7.54 (d, *J* = 6.1 Hz, 1H), 7.10 (dd, *J* = 4.9, 4.0 Hz, 1H), 3.77 (s, 3H), 3.37 (s, 3H) ppm.

^{13}C{^{1}H} NMR (125 MHz, $CDCl_3$): δ = 161.9, 134.0, 133.0, 132.1, 126.6, 61.3, 32.7 ppm.

The analytical data are in accordance with the literature.[5]

N-Methoxy-N-methylcyclohexanecarboxamide (3h)

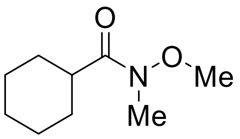

According to **GP 1**, *N,O*-dimethylhydroxylamine hydrochloride (732 mg, 7.5 mmol, 1.0 eq.) was dissolved in dichloromethane under a nitrogen atmosphere and triethylamine (1.59 g, 15.8 mmol, 2.1 eq.) and cyclohexanecarbonyl chloride (1.10 g, 7.5 mmol, 1.0 eq.) were added and stirred for 24 hours. After work-up as described, the product (1.28 g, 7.47 mmol, 99%) was obtained as a yellow liquid.

^{1}H NMR (300 MHz, $CDCl_3$): δ = 3.67 (s, 3H), 3.15 (s, 3H), 2.69-2.62 (m, 1H), 1.79-1.65 (m, 5H), 1.52-1.39 (m, 2H), 1.33-1.14 (m, 3H) ppm.

^{13}C{^{1}H} NMR (125 MHz, $CDCl_3$): δ = 177.4, 61.4, 39.8, 32.1, 28.9, 25.77, 25.76 ppm.

The analytical data are in accordance with the literature.[3]

N-Methoxy-N-methyloctanamide (3i)

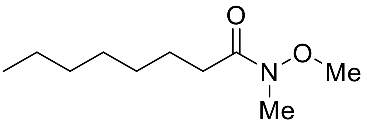

According to **GP 1**, *N,O*-dimethylhydroxylamine hydrochloride (732 mg, 7.5 mmol, 1.0 eq.) was dissolved in dichloromethane under a nitrogen atmosphere and triethylamine (1.59 g, 15.8 mmol, 2.1 eq.) and octanoic acid chloride (1.22 g, 7.5 mmol, 1.0 eq.) were added and stirred for 24 hours. After work-up as described, the product (1.25 g, 6.70 mmol, 89%) was obtained as a yellow liquid.

^{1}H NMR (300 MHz, $CDCl_3$): δ = 3.63 (s, 3H), 3.12 (s, 3H), 2.36 (t, J = 7.2 Hz, 2H), 1.56 (quin, J = 7.2 Hz, 2H),1.34-1.18 (m, 8H) 0.81 (t, J = 6.9 Hz, 3H) ppm.

^{13}C{^{1}H} NMR (125 MHz, $CDCl_3$): δ = 174.7, 61.1, 32.0, 31.8, 31.6, 29.3, 29.0, 24.6, 22.5, 14.0 ppm.

The analytical data are in accordance with the literature.[6]

3. Synthesis of Ketones from Weinreb Amides with 2-(Ethylhexyl)sodium

GP 2: General procedure for preparation and activation of the sodium fixed-bed reactor

An oven-dried Omnifit® Labware glass column (length: 25 cm; inner diameter: 6.6 mm) was sealed on one side with a non-adjustable PTFE end piece. The sodium dispersion (30 wt% in toluene, particle size <0.1 mm, 5 mL) was injected into the glass column using a 10 mL syringe without a cannula. The column was sealed with the adjustable PTFE end piece. The non-adjustable PTFE end piece was connected to the pump with a tube (PTFE Teflon, I.D. = 0.8 mm) while the adjustable end piece was fitted with another tube (PTFE Teflon, I.D. = 0.8 mm), which led to the reaction flask. The fixed-bed reactor was placed upright (with the reactor inlet pointing downwards) in a graduated cylinder filled with *i*-propanol (V = 500 mL) to maintain a temperature of 20 °C. After washing with *n*-hexane (run time: 10 min, flow rate: 2.0 mL/min), the sodium was activated by pumping a solution of *iso*-propanol (0.1 M in *n*-hexane, run time: 2 min, flow rate: 5.0 mL/min). Subsequently, a solution of 3-(chloromethyl)heptane (0.2 M in *n*-hexane, run time: 15 min, flow rate: 2.0 mL/min) was pumped through the column.[1]

GP 3: General procedure for the synthesis of (2-ethylhexyl)sodium (2)

A solution of 3-(chloromethyl)heptane (0.2 M) in *n*-hexane was prepared. The solution was pumped through the activated sodium fixed-bed reactor (see **GP 2**, flow rate: 2.0 mL/min) at 20 °C. Subsequently, after reaching flow equilibrium, the 2-(ethylhexyl)sodium was injected into a flask for the corresponding time.

GP 4: General procedure for the preparation of ketones

According to **GP 3**, (2-ethylhexyl)sodium (1.10 eq., 2 mL/min) was injected into an oven-dried Schlenk flask filled with TMEDA (1.10 eq.) and the Weinreb amide (1.00 eq.) in anhydrous *n*-hexane. The reaction solution was stirred for one hour at room temperature. The solvent was distilled under reduced pressure and the product was obtained after column chromatographic purification.

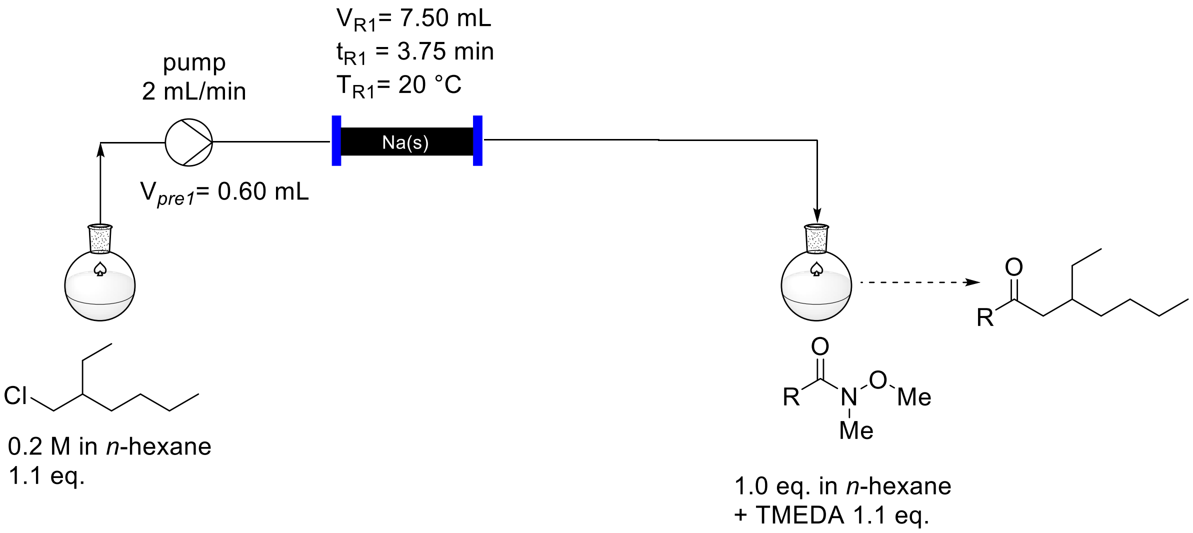

3-Ethyl-1-phenylheptan-1-one (4a)

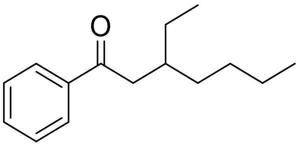

According to **GP 4**, *N*-methoxy-*N*-methylbenzamide (496 mg, 3.0 mmol, 1.0 eq.) and TMEDA (383 mg, 3.3 mmol, 1.1 eq.) were added in *n*-hexane. Subsequently, (2-ethylhexyl)sodium (0.2 M, 16.5 mL, 3.3 mmol, 1.1 eq.) was added and stirred for one hour. The solvent was removed under reduced pressure. The crude product was purified by column chromatography to give the product (587 mg, 2.7 mmol, 90%) as a yellow liquid.

$\mathbf{R_f}$ (SiO_2, dichloromethane) = 0.73.

^{1}H NMR (300 MHz, $CDCl_3$): δ = 7.97-7.94 (m, 1H), 7.58-7.43 (m, 3H), 2.88-2.86 (m, 2H), 2.08-2.00 (m, 2H), 1.43-1.28 (m, 8H), 0.91-0.86 (m, 6H) ppm.

^{13}C{^{1}H} NMR (75 MHz, $CDCl_3$): δ = 201.0, 137.6, 132.9, 128.7, 128.2, 43.2, 35.8, 33.4, 29.0, 26.6, 23.1, 14.2, 11.0 ppm.

The analytical data are in accordance with the literature.[12]

3-Ethyl-1-[4-(trifluoromethyl)phenyl]heptan-1-one (4b)

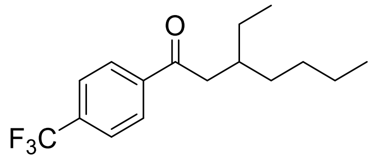

According to **GP 4**, *N*-methoxy-*N*-methyl-4-trifluoromethylbenzamid (700 mg, 3.0 mmol, 1.0 eq.) and TMEDA (383 mg, 3.3 mmol, 1.1 eq.) were added in *n*-hexane. Subsequently, (2-ethylhexyl)sodium (0.2 M, 16.5 mL, 3.3 mmol, 1.1 eq.) was added and stirred for one hour. The solvent was removed under reduced pressure. The crude product was purified by column chromatography to give the product (831 mg, 2.9 mmol, 97%) as a yellow liquid.

$\mathbf{R_f}$ (SiO_2, dichloromethane) = 0.92.

^{1}**H NMR** (300 MHz, $CDCl_3$): δ = 8.04 (d, J = 8.2 Hz, 2H), 7.70 (d, J = 8.2 Hz, 2H), 2.88 (d, J = 6.6 Hz, 1H), 2.05-1.99 (m, 2H), 1.43-1.26 (m, 8H), 0.90-0.85 (m, 6H) ppm.

^{13}**C{^{1}H} NMR** (75 MHz, $CDCl_3$): δ = 200.2, 140.7, 134.6 (q, J_{CF}= 32.7 Hz), 128.9, 126.1 (q, J_{CF} = 3.9 Hz), 124.2 (q, J = 272.6 Hz), 43.9, 36.2, 33.8, 29.4, 27.0, 23.5, 14.6, 11.4 ppm.

^{19}**F NMR** (470 MHz, $CDCl_3$): δ = −63.18 ppm.

IR: $\tilde{\nu}$ = 2960, 2930, 2875, 2860, 1690, 1581, 1511, 1461, 1410, 1380, 1321, 1281, 1208, 1167, 1128, 1108, 1065, 1012, 982, 908, 854, 827, 757, 734, 678, 604, 505 cm^{-1}.

HRMS (EI): calcd. for $C_{16}H_{21}F_3O$ ($[M]^+$): m/z = 286.1544; found: m/z = 286.1537.

3-Ethyl-1-(*p*-tolyl)heptan-1-one (4c)

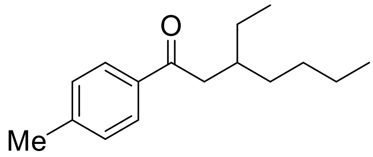

According to **GP 4**, *N*-methoxy-*N*,4-dimethylbenzamide (548 mg, 3.0 mmol, 1.0 eq.) and TMEDA (383 mg, 3.3 mmol, 1.1 eq.) were added in *n*-hexane. Subsequently, (2-ethylhexyl)sodium (0.2 M, 16.5 mL, 3.3 mmol, 1.1 eq.) was added and stirred for one hour. The solvent was removed under reduced pressure. The crude product was purified by column chromatography to give the product (667 mg, 2.8 mmol, 96%) as a yellow liquid.

$\mathbf{R_f}$ (SiO_2, dichloromethane) = 0.77.

^{1}**H NMR** (300 MHz, $CDCl_3$): δ = 7.78 (d, *J* = 8.2 Hz, 2H), 7.17 (d, *J* = 7.8 Hz, 2H), 2.76 (d, *J* = 6.7 Hz, 2H), 2.33 (s, 3H), 2.00-1.91 (m, 1H), 1.35-1.20 (m, 8H), 0.80 (t, *J* = 7.4 Hz, 6H) ppm.

^{13}**C{^{1}H} NMR** (75 MHz, $CDCl_3$): δ = 200.6, 143.6, 135.2, 129.3, 128.3, 43.1, 35.9, 33.4, 29.0, 26.6, 23.1, 21.7, 14.2, 11.0 ppm.

The analytical data are in accordance with the literature.[13]

3-Ethyl-1-(4-methoxyphenyl)heptan-1-one (4d)

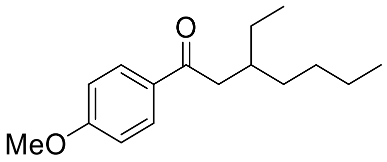

According to **GP 4**, *N*,4-dimethoxy-*N*-methylbenzamide (586 mg, 3.0 mmol, 1.0 eq.) and TMEDA (383 mg, 3.3 mmol, 1.1 eq.) were added in *n*-hexane. Subsequently, (2-ethylhexyl)sodium (0.2 M, 16.5 mL, 3.3 mmol, 1.1 eq.) was added and stirred for one hour. The solvent was removed under reduced pressure. The crude product was purified by column chromatography to give the product (723 mg, 2.9 mmol, 97%) as a yellow liquid.

$\mathbf{R_f}$ (SiO_2, dichloromethane) = 0.63.

^{1}**H NMR** (300 MHz, $CDCl_3$): δ = 7.93 (d, *J* = 8.9 Hz, 2H), 6.92 (d, *J* = 8.9 Hz, 2H), 3.85 (s, 3H), 2.80 (d, *J* = 6.7 Hz, 2H), 2.00 (q, *J* = 6.2 Hz, 1H), 1.71-1.15 (m, 8H), 0.99-0.61 (m, 6H) ppm.

^{13}**C{^{1}H} NMR** (75 MHz, $CDCl_3$): δ = 199.5, 163.3, 130.7, 130.5, 113.7, 55.5, 42.8, 36.0, 33.4, 29.0, 26.6, 23.1, 14.2, 11.0 ppm.

IR: $\tilde{\nu}$ = 2957, 2927, 2873, 2857, 1674, 1599, 1576, 1509, 1460, 1417, 1367, 1307, 1256, 1216, 1167, 1110, 1039, 1004, 980, 960, 827, 809, 773, 727, 633, 609, 590, 579, 511 cm^{-1}.

HRMS (EI): calcd. for $C_{16}H_{24}O_2$ ($[M]^+$): m/z = 248.1776; found: m/z = 248.1776.

4-(3-Ethylheptanoyl)benzonitrile (4e)

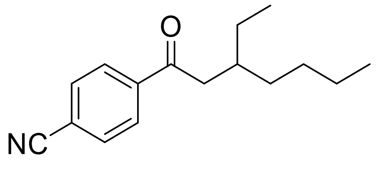

According to **GP 4**, 4-cyano-*N*-methoxy-*N*-methylbenzamide (571 mg, 3.0 mmol, 1.0 eq.) and TMEDA (383 mg, 3.3 mmol, 1.1 eq.) were added in *n*-hexane. Subsequently, (2-

ethylhexyl)sodium (0.2 M, 16.5 mL, 3.3 mmol, 1.1 eq.) was added and stirred for one hour. The solvent was removed under reduced pressure. The crude product was purified by column chromatography to give the product (541 mg, 2.2 mmol, 74%) as a colorless liquid.

R_f (SiO_2, dichloromethane) = 0.31.

^{1}H NMR (300 MHz, $CDCl_3$): δ = 8.01 (d, *J* = 8.4 Hz, 2H), 7.75 (d, *J* = 8.4 Hz, 2H), 2.87 (d, *J* = 6.6 Hz, 2H), 2.01 (p, *J* = 6.0 Hz, 1H), 1.41-1.24 (m, 8H), 0.89-0.84 (m, 6H) ppm.

^{13}C{^{1}H} NMR (75 MHz, $CDCl_3$): δ = 199.4, 140.5, 132.6, 128.6, 118.1, 116.2, 43.4, 35.6, 33.3, 28.9, 26.5, 23.0, 14.6, 11.0 ppm.

IR: $\tilde{\nu}$ = 2960, 2929, 2973, 2859, 2256, 2232, 1687, 1607, 1567, 1462, 1403, 1280, 1209, 1176, 1014, 983, 960, 909, 853, 824, 729, 649, 579, 564, 544 cm^{-1}.

HRMS (EI): calcd. for $C_{16}H_{21}NO$ ($[M]^+$): m/z = 243.1623; found: m/z = 243.1608.

3-Ethyl-1-thiophen-2-ylheptan-1-one (4f)

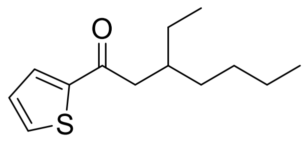

According to **GP 4**, *N*-methoxy-*N*-methylthiophene-2-carboxamide (514 mg, 3.0 mmol, 1.0 eq.) and TMEDA (383 mg, 3.3 mmol, 1.1 eq.) were added in *n*-hexane. Subsequently, (2-ethylhexyl)sodium (0.2 M, 16.5 mL, 3.3 mmol, 1.1 eq.) was added and stirred for one hour. The solvent was removed under reduced pressure. The crude product was purified by column chromatography to give the product (650 mg, 2.9 mmol, 97%) as a yellow liquid.

R_f (SiO_2, dichloromethane) = 0.80.

^{1}H NMR (300 MHz, $CDCl_3$): δ = 7.67 (d, *J* = 3.7 Hz, 1H), 7.58 (d, *J* = 5.6 Hz, 1H), 7.10-7.07 (m, 1H), 2.77 (d, *J* = 6.7 Hz, 2H), 2.05-1.97 (m, 1H), 1.43-1.25 (m, 8H), 0.88-0.83 (m, 6H) ppm.

^{13}C{^{1}H} NMR (75 MHz, $CDCl_3$): δ = 193.6, 145.1, 133.4, 131.7, 128.1, 43.9, 36.3, 33.2, 28.9, 26.5, 23.0, 14.1, 10.9 ppm.

IR: $\tilde{\nu}$ = 2957, 2927, 2873, 2857, 1655, 1518, 1460, 1414, 1378, 1354, 1281, 1232, 1184, 1081, 1057, 1040, 978, 908, 857, 754, 717, 658, 647, 608 cm^{-1}.

HRMS (EI): calcd. for $C_{13}H_{20}OS$ ($[M]^+$): m/z = 224.1235; found: m/z = 224.2140.

3-Ethyl-1-pyridin-3-ylheptan-1-one (4g)

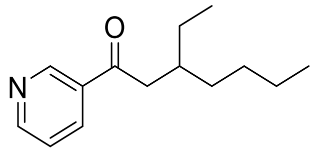

According to **GP 4**, *N*-methoxy-*N*-methylnicotinamide (499 mg, 3.0 mmol, 1.0 eq.) and TMEDA (383 mg, 3.3 mmol, 1.1 eq.) were added in *n*-hexane. Subsequently, (2-ethylhexyl)sodium (0.2 M, 16.5 mL, 3.3 mmol, 1.1 eq.) was added and stirred for one hour. The solvent was removed under reduced pressure. The crude product was purified by column chromatography to give the product (614 mg, 2.8 mmol, 93%) as a yellow liquid.

$\mathbf{R_f}$ (SiO_2, dichloromethane) = 0.65.

^{1}**H NMR** (300 MHz, $CDCl_3$): δ = 9.13 (d, *J* = 1.8 Hz, 1H), 8.73 (dd, *J* = 4.8, 1.5 Hz, 1H), 8.19 (dt, *J* = 8.0, 1.8 Hz, 1H), 7.41-7.36 (m, 1H), 2.85 (d, *J* = 6.7 Hz, 2H), 2.00 (q, *J* = 6.3 Hz, 1H), 1.41-1.16 (m, 8H), 0.88-0.79 (m, 6H) ppm.

^{13}**C{^{1}H} NMR** (75 MHz, $CDCl_3$): δ = 199.6, 153.3, 149.7, 135.5, 132.6, 123.7, 43.4, 35.6, 33.3, 28.9, 26.5, 23.0, 14.1, 11.0 ppm.

IR: $\tilde{\nu}$ = 2959, 2927, 2873, 2859, 1687, 1584, 1572, 1463, 1417, 1379, 1284, 1224, 1196, 1116, 1043, 1026, 1011, 910, 803, 731, 703, 683, 647, 620, 606 cm^{-1}.

HRMS (EI): calcd. for $C_{14}H_{21}NO$ ($[M]^+$): m/z = 219.1623; found: m/z = 219.1621.

1-Cyclohexyl-3-ethylheptan-1-one (4h)

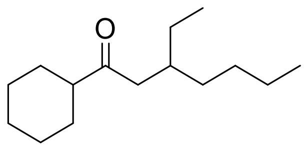

According to **GP 4**, *N*-methoxy-*N*-methylcyclohexanecarboxamide (514 mg, 3.0 mmol, 1.0 eq.) and TMEDA (383 mg, 3.3 mmol, 1.1 eq.) were added in *n*-hexane. Subsequently, (2-ethylhexyl)sodium (0.2 M, 16.5 mL, 3.3 mmol, 1.1 eq.) was added and stirred for one hour. The solvent was removed under reduced pressure. The crude product was purified by column chromatography to give the product (631 mg, 2.8 mmol, 94%) as a yellow liquid.

$\mathbf{R_f}$ (SiO_2, dichloromethane) = 0.91.

^{1}**H NMR** (300 MHz, $CDCl_3$): δ = 2.30-2.22 (m, 3H), 1.86-1.59 (m, 6H), 1.33-1.14 (m, 13H), 0.84-0.76 (m, 6H) ppm.

$^{13}C\{^1H\}$ **NMR** (75 MHz, $CDCl_3$): δ = 214.4, 51.2, 45.4, 34.8, 33.3, 28.9, 28.5, 26.5, 25.9, 25.8, 23.0, 14.1, 10.9 ppm.

IR: $\tilde{\nu}$ = 2957, 2927, 2854, 1705, 1450, 1407, 1377, 1145, 1061, 1007, 917, 894, 732, 647, 515 cm^{-1}.

HRMS (EI): calcd. for $C_{15}H_{28}O$ ($[M]^+$): m/z = 224.2140; found: m/z = 224.2140.

4. Preparation of Ketones with Secondary Sodium Compound

N,N-Dimethyl-4-ethylaniline (5k) and 4-ethyl-*N*-methylaniline (5j)

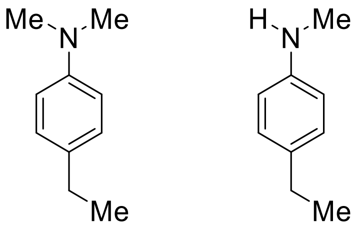

In an oven-dried 50 mL Schlenk flask, sodium hydride (60% in mineral oil, 1.1 g, 28 mmol, 3.1 eq.) was suspended in anhydrous *N,N*-dimethylformamide under a nitrogen atmosphere. At 0 °C, 4-ethylaniline (1.0 g, 9.0 mmol, 1.0 eq.) and then methyl iodide (1.30 g, 9.0 mmol, 1.0 eq.) were added slowly. The reaction mixture was stirred overnight at room temperature. Water was then added and the phases separated. The aqueous phase was washed with diethyl ether (3 x 100 mL) and the combined organic phases were extracted with brine (100 mL). The organic phase was dried over magnesium sulfate, filtered and the solvent removed under reduced pressure. The crude product was purified by column chromatography to give *N,N*-dimethyl-4-ethylaniline (378 mg, 2.53 mmol, 28%) and 4-ethyl-*N*-methylaniline (455 mg, 3.37 mmol, 37%) as orange liquids.[7]

N,N-Dimethyl-4-ethylaniline (5k)

$\mathbf{R_f}$ (SiO_2, *n*-pentane: diethyl ether = 10:1) = 0.56.

^1H **NMR** (300 MHz, $CDCl_3$): δ = 7.13 (d, *J* = 8.2 Hz, 2H), 6.76 (d, *J* = 8.3 Hz, 2H), 2.95 (s, 6H), 2.61 (q, *J* = 7.5 Hz, 2H), 1.25 (t, *J* = 7.9 Hz, 3H) ppm.

^{13}C **NMR** (75 MHz, $CDCl_3$): δ = 149.0, 132.8, 128.5, 113.3, 41.2, 27.9, 16.1 ppm.

The analytical data are in accordance with the literature.[8]

4-Ethyl-*N*-methylaniline (5j)

$\mathbf{R_f}$ (SiO_2, *n*-pentane: diethyl ether = 10:1) = 0.21.

^{1}H NMR (300 MHz, $CDCl_3$): δ = 7.07 (d, *J* = 8.2 Hz, 2H), 6.61 (d, *J* = 8.4 Hz, 2H), 3.53 (br. s, 1H), 2.85 (s, 3H), 2.59 (q, *J* = 7.5 Hz, 2H), 1.23 (t, *J* = 7.6 Hz, 3H) ppm.

$^{13}C\{^1H\}$ NMR (75 MHz, $CDCl_3$): δ = 147.4, 133.3, 128.6, 112.7, 31.2, 28.1, 16.2 ppm

The analytical data are in accordance with the literature.[9]

2-Ethylanisole (5m)

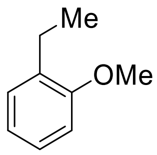

In an oven-dried 100 mL Schlenk flask, sodium hydride (60% in mineral oil, 0.78 g, 19.5 mmol, 1.3 eq.) was added to anhydrous tetrahydrofuran (15 mL) under a nitrogen atmosphere. The solution was then cooled to 0 °C and stirred for 15 minutes. Then 2-ethylphenol (1.83 g, 15.0 mmol, 1.0 eq.) was slowly added and stirred for one hour. Methyl iodide (2.13 g, 15 mmol, 1.0 eq.) was then added and stirred for 12 hours at room temperature. Water was then added to the reaction solution and the phases separated. The aqueous phase was extracted with diethyl ether (3 x 15 mL). The combined organic fractions were dried over magnesium sulfate and filtered. The solvent was removed under reduced pressure. The crude product was purified by column chromatography so that the product (1.26 g, 9.3 mmol, 62%) could be obtained as a colorless liquid.[10]

R_f (SiO_2, dichloromethane) = 0.80.

^{1}H NMR (300 MHz, $CDCl_3$): δ = 7.22 (d, *J* = 7.6 Hz, 2H), 6.96 (t, *J* = 7.4 Hz, 1H), 6.90 (d, *J* = 7.9 Hz, 1H), 3.87 (s, 3H), 2.72 (q, *J* = 7.5 Hz, 2H), 1.27 (t, *J* = 7.5 Hz, 3H) ppm.

$^{13}C\{^1H\}$ NMR (75 MHz, $CDCl_3$): δ = 157.4, 132.7, 129.0, 126.9, 120.5, 110.2, 55.3, 23.4, 14.3 ppm.

The analytical data are in accordance with the literature.[11]

3-Ethylanisole (5n)

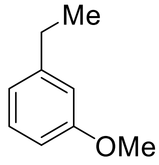

In an oven-dried 100 mL Schlenk flask, sodium hydride (60% in mineral oil, 0.78 g, 19.5 mmol, 1.3 eq.) was added to anhydrous tetrahydrofuran (15 mL) under a nitrogen atmosphere. The solution was then cooled to 0 °C and stirred for 15 minutes. Then 3-ethylphenol (1.83 g, 15.0 mmol, 1.0 eq.) was slowly added and stirred for one hour. Methyl iodide (2.13 g, 15 mmol, 1.0 eq.) was then added and stirred for 12 hours at room temperature. Water was then added to the reaction solution and the phases separated. The aqueous phase was extracted with diethyl ether (3 x 15 mL). The combined organic fractions were dried over magnesium sulfate and filtered. The solvent was removed under reduced pressure. The crude product was purified by column chromatography so that the product (1.45 g, 10.7 mmol, 71%) could be obtained as a colorless liquid.[10]

R_f (SiO_2, dichloromethane) = 0.80.

^{1}H NMR (300 MHz, $CDCl_3$): δ = 7.30 (t, *J* = 7.8 Hz, 1H), 6.94-6.78 (m, 3H), 3.88 (s, 3H), 2.73 (q, *J* = 7.6 Hz, 2H), 1.35 (t, *J* = 7.6 Hz, 3H) ppm.

^{13}C{^{1}H} NMR (75 MHz, $CDCl_3$): δ = 159.7, 146.0, 129.3, 120.4, 133.7, 110.9, 55.1, 29.0, 15.6 ppm.

The analytical data are in accordance with the literature.[11]

GP 5: General procedure for preparation of ketones with secondary sodium compound

According to **GP 3**, (2-ethylhexyl)sodium (1.10 eq., 2 mL/min) was injected into an oven-dried Schlenk flask filled with TMEDA (1.10 eq.) and the corresponding substrate (1.50 eq.) in anhydrous *n*-hexane. The reaction solution was stirred for 15 minutes at room temperature and then *N*-methoxy-*N*-methylbenzamide (1.0 eq.) was added. The reaction solution was stirred for one hour at room temperature. The solvent was removed under reduced pressure and the product was obtained after column chromatographic purification.

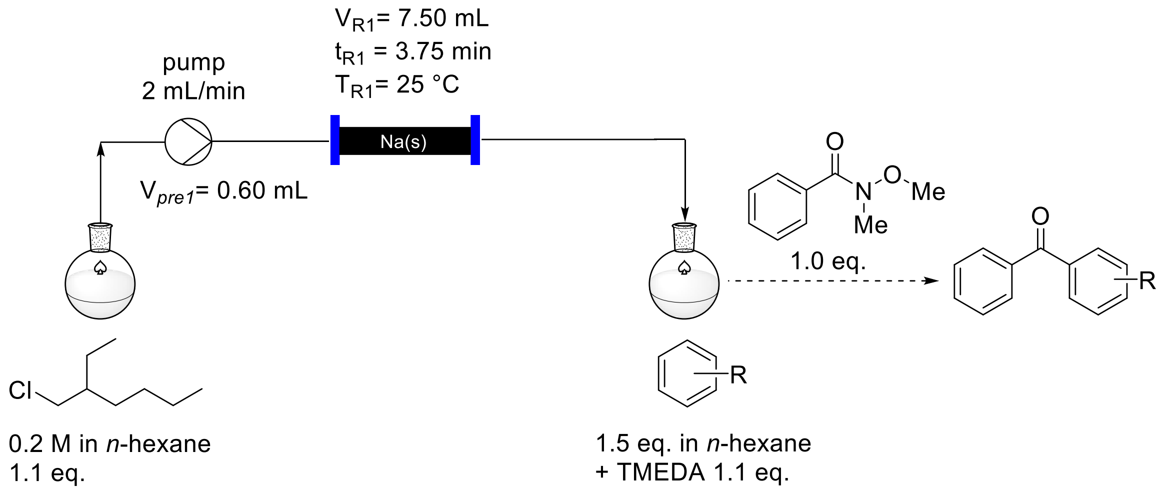

1,2-Diphenylpropan-1-one (7a)

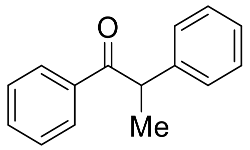

According to **GP 5**, (2-ethylhexyl)sodium (0.2 M, 16.5 mL, 3.3 mmol, 1.1 eq.) was injected in an oven-dried Schlenk flask filled with TMEDA (383 mg, 3.3 mmol, 1.1 eq.) and ethylbenzene (478 mg, 4.5 mmol, 1.50 eq.) in anhydrous *n*-hexane. The reaction solution was stirred for 15 minutes at room temperature and then *N*-methoxy-*N*-methylbenzamide (496 mmol, 3.0 mmol, 1.0 eq.) was added. The reaction solution was stirred for one hour at room temperature. The solvent was removed under reduced pressure. The crude product was purified by column chromatography (SiO_2, dichloromethane) to give the product (432 mg, 2.1 mmol, 68%) as a yellow liquid.

^{1}H NMR (300 MHz, CD_2Cl_2): δ = 7.95 (d, *J* = 8.6 Hz, 2H), 7.53-7.48 (m, 1H), 7.43-7.38 (m, 2H), 7.31-7.19 (m, 5H), 4.72 (q, *J* = 6.8 Hz, 1H), 1.51 (d, *J* = 6.8 Hz, 3H) ppm.

^{13}C{^{1}H} NMR (75 MHz, CD_2Cl_2): δ = 200.6, 142.1, 137.0, 133.3, 129.4, 129.2, 129.0, 128.3, 127.4, 48.3, 19.7 ppm.

The analytical data are in accordance with the literature.[14]

2-(4-Ethylphenyl)-1-phenylethan-1-one (7b)

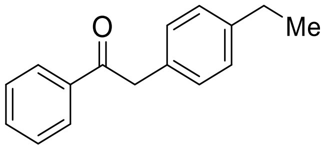

According to **GP 5**, (2-ethylhexyl)sodium (0.2 M, 16.5 mL, 3.3 mmol, 1.1 eq.) was injected in an oven-dried Schlenk flask filled with TMEDA (383 mg, 3.3 mmol, 1.1 eq.) and 4-ethyltoluene (541 mg, 4.5 mmol, 1.5 eq.) in anhydrous *n*-hexane. The reaction solution was stirred for 15 minutes at room temperature and then *N*-methoxy-*N*-methylbenzamide was added (496 mg, 3.0 mmol, 1.0 eq.). The reaction solution was stirred for one hour at room temperature. The solvent was removed under reduced pressure. The crude product was purified by column chromatography to give the product (571 mg, 2.6 mmol, 85%) as a colorless solid.

R_f (SiO_2, dichloromethane) = 0.78.

^{1}H NMR (300 MHz, $CDCl_3$): δ = 7.92-7.85 (m, 2H), 7.42-7.36 (m, 1H), 7.34-7.26 (m, 2H), 7.11-6.99 (m, 4H), 4.11 (s, 2H), 2.49 (q, *J* = 7.6 Hz, 2H), 1.09 (t, *J* = 7.6 Hz, 3H) ppm.

^{13}C{^{1}H} NMR (75 MHz, $CDCl_3$): δ = 197.8, 142.8, 136.6, 133.1, 131.7, 129.4, 128.6, 128.2, 45.1, 28.5, 15.6 ppm.

IR: $\tilde{\nu}$ = 3054, 3025, 2971, 2921, 2894, 1907, 1801, 1684, 1592, 1578, 1514, 1462, 1448, 1418, 1405, 1380, 1330, 1311, 1220, 1201, 1184, 1158, 1120, 1078, 1052, 1022, 994, 982, 942, 910, 862, 842, 825, 810, 795, 761, 732, 691, 652, 638, 615, 568, 528, 495 cm^{-1}.

HRMS (EI): calcd. for $C_{16}H_{16}O$ ($[M]^+$): m/z = 224.1201; found: m/z = 224.1202.

The analytical data are in accordance with the literature.[15]

Phenyl(1,2,3,4-tetrahydronaphthalen-1-yl)methanone (7d)

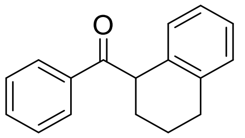

According to **GP 5**, (2-ethylhexyl)sodium (0.2 M, 16.5 mL, 3.3 mmol, 1.1 eq.) was injected in an oven-dried Schlenk flask filled with TMEDA (383 mg, 3.3 mmol, 1.1 eq.) and 1,2,3,4-tetrahydronaphthalene (595 mg, 4.5 mmol, 1.5 eq.) in anhydrous *n*-hexane. The reaction solution was stirred for 15 minutes at room temperature and then *N*-methoxy-*N*-methylbenzamide (496 mg, 3.0 mmol, 1.0 eq.) was added. The reaction solution was stirred for one hour at room temperature. The solvent was removed under reduced pressure. The

crude product was purified by column chromatography to give the product (608 mg, 2.6 mmol, 86%) as a colorless solid.

R$_f$ (SiO_2, dichloromethane) = 0.73.

^{1}H NMR (300 MHz, $CDCl_3$): δ = 8.07-8.04 (m, 2H), 7.64-7.49 (m, 3H), 7.21-7.09 (m, 3H), 6.96 (d, *J* = 7.5 Hz, 1H), 4.88 (t, *J* = 6.6 Hz, 1H), 3.00-2.80 (m, 2H), 2.27-2.06 (m, 2H), 2.04-1.91 (m, 1H), 1.88-1.74 (m, 1H) ppm.

^{13}C{^{1}H} NMR (75 MHz, $CDCl_3$): δ = 202.6, 137.8, 136.6, 134.8, 133.1, 129.5, 129.5, 128.8, 128.8, 126.7, 125.9, 47.4, 29.3, 27.7, 20.7 ppm.

The analytical data are in accordance with the literature.[16]

2-Benzoyl-3-methoxy-5,6,7,8-tetrahydronaphthaline (7e)

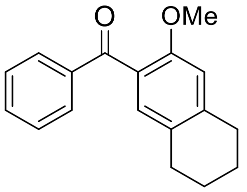

According to **GP 5**, (2-ethylhexyl)sodium (0.2 M, 16.5 mL, 3.3 mmol, 1.1 eq.) was injected in an oven-dried Schlenk flask filled with TMEDA (383 mg, 3.3 mmol, 1.1 eq.) and 6-methoxy-1,2,3,4-tetrahydronaphthalene (730 mg, 4.5 mmol, 1.5 eq.) in anhydrous *n*-hexane. The reaction solution was stirred for 15 minutes at room temperature and then *N*-methoxy-*N*-methylbenzamide (496 mg, 3.0 mmol, 1.0 eq.) was added. The reaction solution was stirred for one hour at room temperature. The solvent was removed under reduced pressure. The crude product was purified by column chromatography to give the product (647 mg, 2.4 mmol, 81%) as a yellow oil.

R$_f$ (SiO_2, dichloromethane) = 0.38.

^{1}H NMR (300 MHz, $CDCl_3$): δ = 7.84-7.78 (m, 2H), 7.51 (t, *J* = 7.3 Hz, 1H), 7.40 (t, *J* = 7.5 Hz, 2H), 7.09 (s, 1H), 6.67 (s, 1H), 3.65 (s, 3H), 2.83-2.79 (m, 2H), 2.72-2.68 (m, 2H), 1.80 (q, *J* = 3.2 Hz, 4H) ppm.

^{13}C{^{1}H} NMR (75 MHz, $CDCl_3$): δ = 196.5, 155.2, 141.5, 138.2, 132.6, 130.4, 129.7, 129.1, 128.0, 126.3, 111.8, 55.5, 29.9, 28.4, 23.2, 22.9 ppm.

IR: $\tilde{\nu}$ = 3058, 2925, 2855, 2835, 1660, 1608, 1597, 1577, 1498, 1462, 1447, 1408, 1354, 1337, 1317, 1270, 1247, 1230, 1195, 1175, 1162, 1097, 1071, 1021, 1001, 962, 904, 885, 864, 847, 817, 805, 752, 732, 702, 690, 681, 645, 615, 535 cm^{-1}.

HRMS (EI): calcd. for $C_{18}H_{18}O_2$ ($[M]^+$): m/z = 266.130; found: m/z = 266.1296.

1-(4-Methoxyphenyl)-2-phenylpropan-1-one (7f)

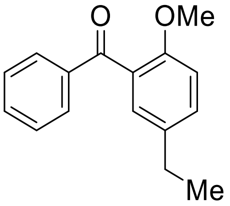

According to **GP 5**, (2-ethylhexyl)sodium (0.2 M, 16.5 mL, 3.3 mmol, 1.1 eq.) was injected into an oven-dried Schlenk flask filled with TMEDA (383 mg, 3.3 mmol, 1.1 eq.) and 1-ethyl-4-methoxybenzene (613 mg, 4.5 mmol, 1.5 eq.) in anhydrous *n*-hexane. The reaction solution was stirred for 15 minutes at room temperature and then *N*-methoxy-*N*-methylbenzamide (496 mg, 3.0 mmol, 1.0 eq.) was added. The reaction solution was stirred for one hour at room temperature. The solvent was removed under reduced pressure. The crude product was purified by column chromatography to give the product (558 mg, 2.32 mmol, 77%) as a yellow liquid.

R_f (SiO_2, dichloromethane) = 0.54.

^{1}H NMR (500 MHz, $CDCl_3$): δ = 7.94 (d, *J* = 7.2 Hz, 2H), 7.61 (t, *J* = 7.4 Hz, 1H), 7.50 (t, *J* = 7.7 Hz, 2H), 7.39 (dd, *J* = 8.5, 2.3 Hz, 1H), 7.32 (d, *J* = 2.3 Hz, 1H), 7.01 (d, *J* = 8.5 Hz, 1H), 3.74 (s, 3H), 2.73 (q, *J* = 7.6 Hz, 2H), 1.33 (t, *J* = 7.6 Hz, 3H) ppm.

^{13}C{^{1}H} NMR (125 MHz, $CDCl_3$): δ = 196.7, 155.6, 138.1, 136.4, 132.9, 131.3, 129.8, 128.9, 128.3, 113.9, 111.8, 55.8, 27.9, 15.7 ppm.

IR: $\tilde{\nu}$ = 3060, 2963, 2932, 2872, 2836, 1660, 1607, 1596, 1582, 1494, 1462, 1449, 1414, 1376, 1314, 1293, 1263, 1239, 1206, 1176, 1154, 1116, 1073, 1060, 1024, 1001, 991, 950, 897, 839, 817, 806, 784, 731, 703, 691, 646, 626, 616, 551 cm^{-1}.

HRMS (EI): calcd. for $C_{16}H_{16}O_2$ ($[M]^+$): m/z = 240.1150; found: m/z = 220.1144.

***N*-(4-Ethylphenyl)benzamide (7i)**

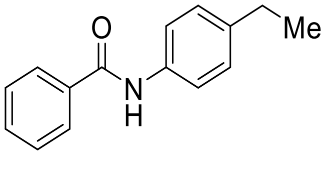

According to **GP 5**, (2-ethylhexyl)sodium (0.2 M, 16.5 mL, 3.3 mmol, 1.1 eq.) was injected in an oven-dried Schlenk flask filled with TMEDA (383 mg, 3.3 mmol, 1.1 eq.) and 4-ethylaniline

(545 mg, 4.5 mmol, 1.5 eq.) in anhydrous *n*-hexane. The reaction solution was stirred for 15 minutes at room temperature and then *N*-methoxy-*N*-methylbenzamide (496 mg, 3.0 mmol, 1.0 eq.) was added. The reaction solution was stirred for one hour at room temperature. The solvent was removed under reduced pressure. The crude product was purified by column chromatography to give the product (536 mg, 2.4 mmol, 79%) as a yellow solid.

R$_f$ (SiO_2, dichloromethane) = 0.39.

^{1}H NMR (300 MHz, $CDCl_3$): δ = 7.94 (br. s, 1H), 7.89-7.81 (m, 2H), 7.60-7.39 (m, 5H), 7.18 (d, *J* = 8.4 Hz, 2H), 2.64 (q, *J* = 7.6 Hz, 2H), 1.23 (t, *J* = 7.6 Hz, 3H) ppm.

^{13}C{^{1}H} NMR (75 MHz, $CDCl_3$): δ = 165.9, 140.8, 135.6, 135.2, 131.8, 128.8, 128.5, 127.1, 120.5, 28.5, 15.8 ppm.

The analytical data are in accordance with the literature.[17]

N-(4-Ethylphenyl)-*N*-methylbenzamide (7j)

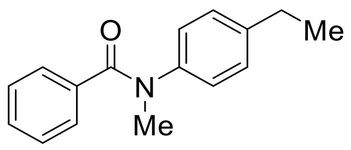

According to **GP 5**, (2-ethylhexyl)sodium (0.2 M, 16.5 mL, 3.3 mmol, 1.1 eq.) was injected into an oven-dried Schlenk flask filled with TMEDA (383 mg, 3.3 mmol, 1.1 eq.) and 4-ethyl-*N*-methylaniline (608 mg, 4.5 mmol, 1.5 eq.) in anhydrous *n*-hexane. The reaction solution was stirred for 15 minutes at room temperature and then *N*-methoxy-*N*-methylbenzamide (496 mg, 3.0 mmol, 1.0 eq.) was added. The reaction solution was stirred for one hour at room temperature. The solvent was removed under reduced pressure. The crude product was purified by column chromatography to give the product (557 mg, 2.3 mmol, 78%) as an orange oil.

R$_f$ (SiO_2, diethyl ether:*n*-pentane = 2:1) = 0.54.

^{1}H NMR (300 MHz, $CDCl_3$): δ = 7.34-7.26 (m, 2H), 7.25-7.08 (m, 3H), 7.03 (d, *J* = 8.3 Hz, 2H), 6.93 (d, *J* = 8.3 Hz, 2H), 3.47 (s, 3H), 2.55 (q, *J* = 7.6 Hz, 2H), 1.16 (t, *J* = 7.6 Hz, 3H) ppm.

^{13}C{^{1}H} NMR (75 MHz, $CDCl_3$): δ = 170.7, 142.6, 142.4, 136.0, 129.5, 128.7, 128.5, 127.7, 126.7, 38.5, 28.2, 15.4 ppm.

IR: $\tilde{\nu}$ = 3030, 2965, 2933, 2874, 2244, 1635, 1605, 1575, 1511, 1494, 1447, 1425, 1362, 1301, 1284, 1178, 1104, 1074, 1050, 1031, 1020, 1010, 908, 878, 837, 791, 725, 695, 680, 645, 614, 577, 532 cm^{-1}.

The analytical data are in accordance with the literature.[18]

(2-(Dimethylamino)-5-ethylphenyl)(phenyl)methanone (7k)

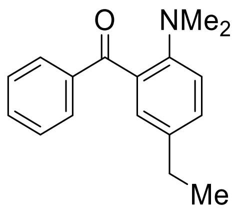

After **GP 3**, (2-ethylhexyl)sodium (0.2 M, 16.5 mL, 3.3 mmol, 1.1 eq.) was injected in an oven-dried Schlenk flask filled with TMEDA (383 mg, 3.3 mmol, 1.1 eq.) and *N*,*N*-dimethyl-4-ethylaniline (672 mg, 4.5 mmol, 1.5 eq.) in anhydrous *n*-hexane. The reaction solution was stirred for 15 minutes at room temperature and then *N*-methoxy-*N*-methylbenzamide was added (496 mg, 3.0 mmol, 1.0 eq.). The reaction solution was stirred for one hour at room temperature. The solvent was removed under reduced pressure. The crude product was purified by column chromatography to give the product (564 mg, 2.2 mmol, 74%) as a yellow liquid.

R_f (SiO_2, *n*-pentane:diethyl ether = 10:1) = 0.26.

^{1}H NMR (300 MHz, $CDCl_3$): δ = 7.89-7.86 (m, 2H), 7.58 (t, *J* = 8.0 Hz, 1H), 7.45 (t, *J* = 7.5 Hz, 2H), 7.31 (dd, *J* = 8.4, 2.2 Hz, 1H), 7.20 (d, *J* = 2.0 Hz, 1H), 7.01 (d, *J* = 8.4 Hz, 1H), 2.69 (s, 6H), 2.64 (q, *J* = 7.6 Hz, 2H), 1.23 (t, *J* = 7.6 Hz, 3H) ppm.

^{13}C{^{1}H} NMR (75 MHz, $CDCl_3$): δ = 198.7, 149.8, 137.7, 135.6, 132.7, 130.9, 130.3, 130.0, 129.6, 128.1, 117.1, 43.9, 27.9, 15.7 ppm.

IR: $\tilde{\nu}$ = 3025, 2964, 2933, 2871, 2790, 2245, 1652, 1607, 1595, 1580, 1562, 1500, 1448, 1411, 1375, 1314, 1291, 1261, 1207, 1174, 1162, 1134, 1104, 1060, 1027, 1001, 991, 957, 940, 907, 830, 802, 728, 718, 700, 688, 645, 615, 564 cm^{-1}.

HRMS (EI): calcd. for $C_{17}H_{19}NO$ ($[M]^+$): m/z = 253.1467; found: m/z = 253.1456.

2,5-Dimethoxybenzophenone (7l)

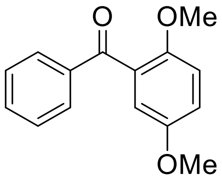

According to **GP 5**, (2-ethylhexyl)sodium (0.2 M, 16.5 mL, 3.3 mmol, 1.1 eq.) was injected into an oven-dried Schlenk flask filled with TMEDA (383 mg, 3.3 mmol, 1.1 eq.) and 1,4-dimethoxybenzene (622 mg, 4.5 mmol, 1.5 eq.) in anhydrous *n*-hexane. The reaction solution was stirred for 15 minutes at room temperature and then *N*-methoxy-*N*-methylbenzamide (496 mg, 3.0 mmol, 1.0 eq.) was added. The reaction solution was stirred for one hour at room temperature. The solvent was removed under reduced pressure. The crude product was purified by column chromatography to give the product (571 mg, 2.4 mmol, 78%) as a yellow oil.

R_f (SiO_2, dichloromethane) = 0.51.

^{1}H NMR (300 MHz, $CDCl_3$): δ = 7.87-7.76 (m, 2H), 7.57-7.51 (m, 1H), 7.47-7.36 (m, 2H), 7.00 (dt, *J* = 9.0, 2.5 Hz, 1H), 6.97-6.86 (m, 2H), 3.76 (d, *J* = 3.4 Hz, 3H), 3.64 (d, *J* = 3.6 Hz, 3H) ppm.

^{13}C{^{1}H} NMR (75 MHz, $CDCl_3$): δ = 196.2, 153.4, 151.4, 137.6, 133.1, 129.8, 129.4, 128.3, 117.3, 114.5, 113.0, 56.3, 55.8 ppm.

The analytical data are in accordance with the literature.[19]

(3-Ethyl-2-methoxyphenyl)(phenyl)methanone (7m)

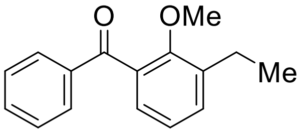

According to **GP 5**, (2-ethylhexyl)sodium (0.2 M, 16.5 mL, 3.3 mmol, 1.1 eq.) was injected into an oven-dried Schlenk flask filled with TMEDA (383 mg, 3.3 mmol, 1.1 eq.) and 1-ethyl-2-methoxybenzene (613 mg, 4.5 mmol, 1.5 eq.) in anhydrous *n*-hexane. The reaction solution was stirred for 15 minutes at room temperature and then *N*-methoxy-*N*-methylbenzamide (496 mg, 3.0 mmol, 1.0 eq.) was added. The reaction solution was stirred for one hour at room temperature. The solvent was removed under reduced pressure. The crude product was purified by column chromatography to give the product (514 mg, 2.1 mmol, 71%) as a yellow liquid.

R$_f$ (SiO_2, dichloromethane) = 0.59.

^{1}H NMR (500 MHz, $CDCl_3$): δ = 7.94-7.91 (m, 2H), 7.63 (tt, *J* = 6.7, 1.2 Hz, 1H), 7.53-7.42 (m, 3H), 7.27 (dd, *J* = 7.6, 1.8 Hz, 1H), 7.19 (t, *J* = 7.5 Hz, 1H), 3.69 (s, 3H), 2.79 (q, *J* = 7.5 Hz, 2H), 1.33 (t, *J* = 7.6 Hz, 3H) ppm.

^{13}C{^{1}H} NMR (125 MHz, $CDCl_3$): δ = 197.0, 156.3, 137.7, 137.4, 133.5, 132.7, 131.9, 130.0, 128.4, 127.4, 123.6, 62.5, 22.7, 14.9 ppm.

IR: $\tilde{\nu}$ = 3063, 2965, 2935, 2873, 2828, 1664, 1597, 1581, 1461, 1448, 1421, 1374, 1315, 1275, 1224, 1190, 1174, 1157, 1125, 1095, 1065, 1051, 1002, 950, 835, 808, 764, 708, 688, 674, 647, 597, 565, 524 cm^{-1}.

HRMS (EI): calcd. for $C_{16}H_{16}O_2$ ($[M]^+$): m/z = 240.1150; found: m/z = 240.1144.

(4-Ethyl-2-methoxyphenyl)(phenyl)methanone (7n)

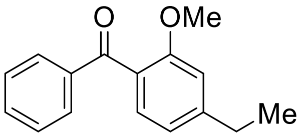

According to **GP 5**, (2-ethylhexyl)sodium (0.2 M, 16.5 mL, 3.3 mmol, 1.1 eq.) was injected in an oven-dried heated Schlenk flask filled with TMEDA (383 mg, 3.3 mmol, 1.1 eq.) and 3-ethylanisole (613 mg, 4.5 mmol, 1.5 eq.) in anhydrous *n*-hexane. The reaction solution was stirred for 15 minutes at room temperature and then *N*-methoxy-*N*-methylbenzamide (496 mg, 3.0 mmol, 1.0 eq.) was added. The reaction solution was stirred for one hour at room temperature. The solvent was removed under reduced pressure. The crude product was purified by column chromatography to give the product (598 mg, 2.5 mmol, 83%) as a yellow oil.

R$_f$ (SiO_2, dichloromethane) = 0.38.

^{1}H NMR (300 MHz, $CDCl_3$): δ = 7.81 (d, *J* = 7.1 Hz, 2H), 7.50 (t, *J* = 7.3 Hz, 1H), 7.39 (t, *J* = 7.5 Hz, 2H), 7.30 (d, *J* = 7.7 Hz, 1H), 6.86 (d, *J* = 8.0 Hz, 1H), 6.82 (s, 1H), 3.68 (s, 3H), 2.69 (q, *J* = 7.6 Hz, 2H), 1.28 (t, *J* = 7.6 Hz, 3H) ppm.

^{13}C{^{1}H} NMR (75 MHz, $CDCl_3$): δ = 196.3, 157.6, 149.1, 138.1, 132.6, 129.9, 129.7, 128.0, 126.1, 119.8, 111.1, 55.4, 29.2, 15.3 ppm.

IR: $\tilde{\nu}$ = 3064, 2967, 2934, 2873, 2250, 1660, 1607, 1581, 1570, 1497, 1462, 1448, 1412, 1215, 1291, 1267, 1251, 1170, 1148, 1120, 1072, 1060, 1034, 1001, 931, 907, 855, 827, 800, 727, 702, 647, 617, 600, 538, 485 cm^{-1}.

HRMS (EI): calcd. for $C_{16}H_{16}O_2$ ($[M]^+$): m/z = 240.1150; found: m/z = 240.1142.

3-Isopropyl-2-methoxybenzophenone (7o)

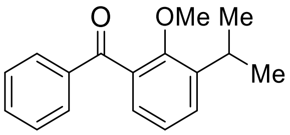

According to **GP 5**, (2-ethylhexyl)sodium (0.2 M, 16.5 mL, 3.3 mmol, 1.1 eq.) was injected in an oven-dried Schlenk flask filled with TMEDA (383 mg, 3.3 mmol, 1.1 eq.) and 2-isopropyl-anisole (676 mg, 4.5 mmol, 1.5 eq.) in anhydrous *n*-hexane. The reaction solution was stirred for 15 minutes at room temperature and then *N*-methoxy-*N*-methylbenzamide (496 mg, 3.0 mmol, 1.0 eq.) was added. The reaction solution was stirred for one hour at room temperature. The solvent was removed under reduced pressure. The crude product was purified by column chromatography to give the product (582 mg, 2.3 mmol, 76%) as a yellow oil.

$\mathbf{R_f}$ (SiO_2, dichloromethane) = 0.60.

^{1}H NMR (300 MHz, $CDCl_3$): δ = 7.88-7.85 (m, 2H), 7.57 (t, *J* = 7.4 Hz, 1H), 7.50-7.39 (m, 3H), 7.24-7.11 (m, 2H), 3.63 (s, 3H), 3.39 (p, *J* = 6.9 Hz, 1H), 1.27 (d, *J* = 6.9 Hz, 6H) ppm.

^{13}C{^{1}H} NMR (75 MHz, $CDCl_3$): δ = 197.1, 155.6, 142.4, 137.5, 133.3, 132.8, 130.1, 129.2, 128.4, 127.2, 123.9, 63.0, 26.4, 23.7 ppm.

IR: $\tilde{\nu}$ = 3064, 2963, 2870, 2828, 2251, 1667, 1597, 1588, 1580, 1460, 1448, 1421, 1384, 1362, 1337, 1317, 1288, 1254, 1222, 1191, 1177, 1161, 1148, 1094, 1072, 1050, 1001, 972, 910, 891, 848, 821, 804, 765, 731, 710, 688, 675, 647, 602, 572, 548 cm^{-1}.

HRMS (EI): calcd. for $C_{17}H_{18}O_2$ ($[M]^+$): m/z = 254.1307; found: m/z = 254.1301.

The analytical data are in accordance with the literature.[20]

3-Isopropyl-2-methoxybenzophenone (7p)

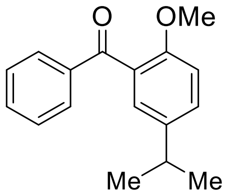

According to **GP 5**, (2-ethylhexyl)sodium (0.2 M, 16.5 mL, 3.3 mmol, 1.1 eq.) was injected in an oven-dried Schlenk flask filled with TMEDA (383 mg, 3.3 mmol, 1.1 eq.) and 4-isopropylanisole (676 mg, 4.5 mmol, 1.5 eq.) in anhydrous *n*-hexane. The reaction solution was stirred for 15 minutes at room temperature and then *N*-methoxy-*N*-methylbenzamide (496 mg, 3.0 mmol, 1.0 eq.) was added. The reaction solution was stirred for one hour at room temperature. The solvent was removed under reduced pressure. The crude product was purified by column chromatography to give the product (578 mg, 2.3 mmol, 76%) as a colorless oil.

R_f (SiO_2, dichloromethane) = 0.45.

^{1}H NMR (300 MHz, $CDCl_3$): δ = 7.83 (d, *J* = 7.2 Hz, 2H), 7.54 (t, *J* = 7.3 Hz, 1H), 7.42 (t, *J* = 7.5 Hz, 2H), 7.33 (dd, *J* = 8.5, 2.3 Hz, 1H), 7.24 (d, *J* = 2.3 Hz, 1H), 6.92 (d, *J* = 8.5 Hz, 1H), 3.68 (s, 3H), 2.90 (p, *J* = 6.9 Hz, 1H), 1.24 (d, *J* = 6.9 Hz, 6H) ppm.

^{13}C{^{1}H} NMR (75 MHz, $CDCl_3$): δ = 196.9, 155.5, 141.0, 137.9, 132.9, 129.9, 129.8, 128.6, 128.2, 127.6, 111.5, 55.7, 33.3, 24.1 ppm.

IR: $\tilde{\nu}$ = 3060, 2958, 2870, 2837, 2250, 1660, 1607, 1595, 1578, 1495, 1461, 1448, 1415, 1382, 1362, 1315, 1295, 1261, 1242, 1210, 1177, 1160, 1115, 1072, 1058, 1024, 1001, 971, 908, 888, 827, 817, 805, 747, 728, 704, 688, 647, 631, 615, 567 cm^{-1}.

HRMS (EI): calcd. for $C_{17}H_{18}O_2$ ($[M]^+$): m/z = 254.1307; found: m/z = 254.1296.

3-(4-Methoxyphenyl)-1-phenylprop-2-yn-1-one (7q)

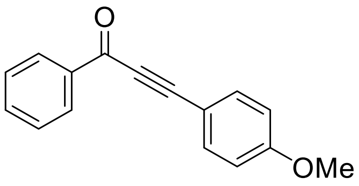

According to **GP 5**, (2-ethylhexyl)sodium (0.2 M, 16.5 mL, 3.3 mmol, 1.1 eq.) was injected in an oven-dried Schlenk flask filled with TMEDA (383 mg, 3.3 mmol, 1.1 eq.) and 4-ethynylanisole (595 mg, 4.5 mmol, 1.5 eq.) in anhydrous *n*-hexane. The reaction solution was stirred for 15 minutes at room temperature and then *N*-methoxy-*N*-methylbenzamide (496 mg,

3.0 mmol, 1.0 eq.) was added. The reaction solution was stirred for one hour at room temperature. The solvent was removed under reduced pressure. The crude product was purified by column chromatography to give the product (389 mg, 1.65 mmol, 55%) as a yellow oil.

R$_f$ (SiO_2, dichloromethane) = 0.69.

^{1}H NMR (300 MHz, $CDCl_3$): δ = 8.21-8.18 (m, 2H), 7.63-7.57 (m, 3H), 7.49 (t, *J* = 7.4 Hz, 2H), 6.92-6.89 (m, 2H), 3.81 (s, 3H) ppm.

^{13}C{^{1}H} NMR (75 MHz, $CDCl_3$): δ = 178.0, 161.8, 137.0, 135.2, 134.0, 129.5, 128.6, 114.4, 111.8, 94.4, 86.9, 55.4 ppm.

The analytical data are in accordance with the literature.[21]

5. Preparation of Ketones from Benzoic Acid Derivatives

5.1 Optimization of the Reaction Conditions

The optimization was carried out using various benzoates (lithium, sodium and potassium) and benzoic acid. A calibration series was recorded in advance using 3-ethyl-1-phenylheptan-1-one with mesitylene as an internal standard. The yields were determined via GC.

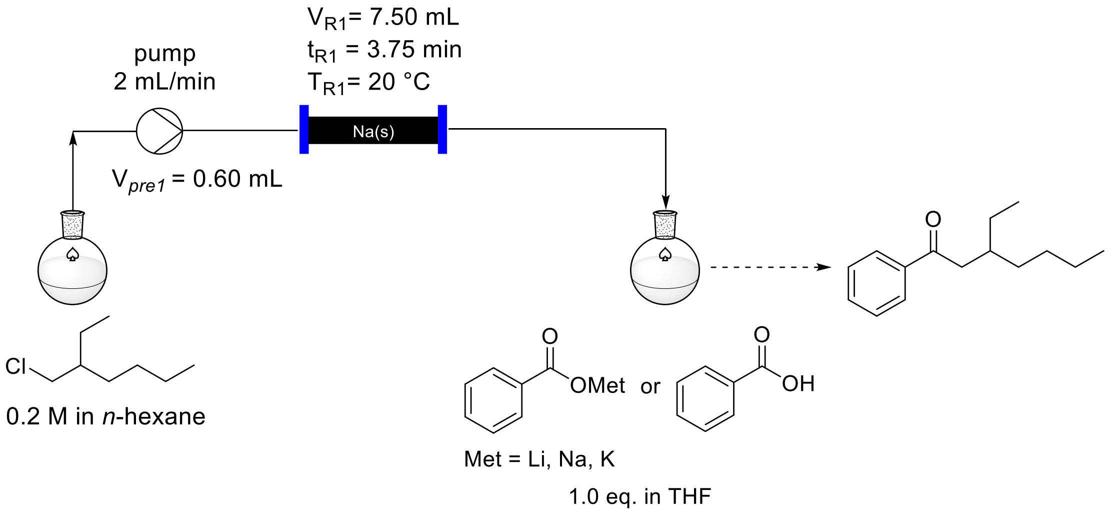

Table 1: Influence of the use of TMEDA on the reaction of lithium benzoate with (2-ethylhexyl)sodium.

Entry	Use of TMEDA	Yield [%]
**1**	without	10
**2**	1.2 eq.	11

Reaction conditions: lithium benzoate (3.0 mmol, 1.0 eq.), (2-ethylhexyl)sodium (3.6 mmol, 1.2 eq.), TMEDA (3.6 mmol, 1.2 eq.) in anhydrous tetrahydrofuran (10 mL), r.t., 16 h.

Table 2: Influence of the counter ion of benzoates and the addition of Lewis acids on the reaction with (2-ethylhexy)sodium.

Entry	Benzoate	Lewis acid	Yield [%]
**1**	Lithium benzoate	without	10
**2**	Sodium benzoate	without	0
**3**	Potassium benzoate	without	0
**4**	Sodium benzoate	$BF_3{\cdot}Et_2O$	8
**5**	Potassium benzoate	$BF_3{\cdot}Et_2O$	6
**6**	Sodium benzoate	LiCl	15
**7**	Potassium benzoate	LiCl	9

Reaction conditions: Alkali benzoate (1.0 mmol, 1.0 eq.), (2-ethylhexyl)sodium (1.5 mmol, 1.5 eq.), Lewis acid (1.0 mmol, 1.0 eq.) in anhydrous tetrahydrofuran, r.t., 16 h.

Table 3: Influence of the amount of (2-ethylhexy)sodium on the reaction with sodium benzoate and LiCl as Lewis acid.

Entry	Amount of (2-ethylhexyl)sodium	Yield [%]
**1**	1.5 eq.	8
**2**	2.0 eq.	8
**3**	2.5 eq.	10

Reaction conditions: Sodium benzoate (1.0 mmol, 1.0 eq.), (2-ethylhexyl)sodium (entry 1-3), lithium chloride (1.0 mmol, 1.0 eq.) in anhydrous tetrahydrofuran, r.t., 16 h.

Table 4: Influence of ultrasound on the reaction of sodium benzoate and (2-ethylhexyl)sodium.

Entry	Ultrasound duration [min]	Ultrasound during/after the reaction at 14	Yield [%]
**1**	15	after	12
**2**	30	after	22
**3**	30	during	20

Reaction conditions: Sodium benzoate (1.0 mmol, 1.0 eq.), lithium chloride (1.0 mmol, 1.0 eq.), (2-ethylhexyl)sodium (1.5 mmol, 1.5 eq.), ultrasonic bath for the described time at 20 °C, r.t., 16 h.

Table 6: Temperature dependence of the reaction of benzoic acid with (2-ethylhexyl)sodium

Entry	Temperature [°C]	Yield [%]
**1**	0	22
**2**	20	29
**3**	40	27
**4**	60	18

Reaction conditions: Benzoic acid (1.0 mmol, 1.0 eq.), lithium chloride (1.0 mmol, 1.0 eq.), (2-ethylhexyl)sodium (3.0 mmol, 3.0 eq.), 30 minutes ultrasonic bath, then 1h at the described temperature (entry 1-4), then r.t. 16 h.

Table 7: Influence of the amount of lithium chloride on the reaction of benzoic acid with (2-ethylhexyl)sodium

Entry	Scale [mmol]	Lewis acid	Amount of Lewis acid	Yield [%]
**1**	1.0	Lithium chloride	0.1	19
**2**	1.0	Lithium chloride	1.0	39
**3**	1.0	Lithium chloride	2.0	67
**4**	1.0	Lithium chloride	4.0	70
**5**	1.0	Lithium chloride	6.0	83
**6**	3.0	Lithium chloride	10.0	65
**7**	1.0	Sodium chloride	1.0	0

Reaction conditions: Benzoic acid (1.0 mmol, 1.0 eq.), Lewis acid (described in entry 1-7), (2-ethylhexyl)sodium (3.0 mmol, 3.0 eq.), 30 minutes ultrasonic bath, then r.t., 16 h.

5.2 Synthesized Ketones from Benzoic Acid Derivatives

GP 6: General procedure for preparation of ketones from benzoic acid derivatives

According to **GP 3**, (2-ethylhexyl)sodium (3.0 eq., 2 mL/min) was injected into an oven-dried Schlenk flask filled with LiCl (6.0 eq.) and the corresponding substrate (1.0 eq.) in anhydrous tetrahydrofuran. The reaction solution was treated in an ultrasonic bath for 30 minutes and then stirred overnight at room temperature. The solvent was removed under reduced pressure and the product was obtained after column chromatographic purification.

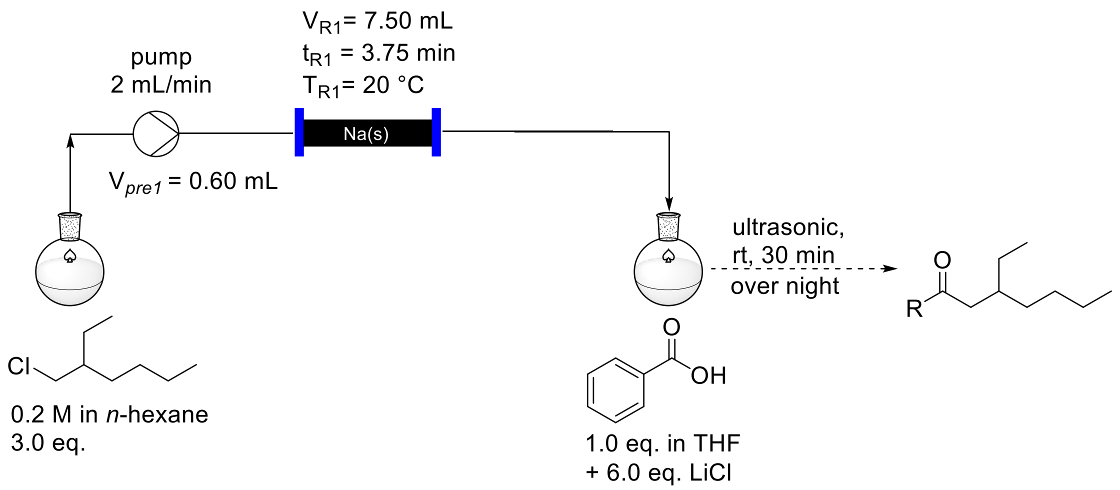

3-Ethyl-1-phenylheptan-1-one (9a)

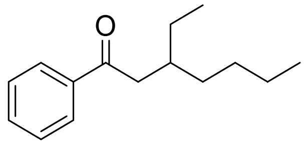

According to **GP 6**, benzoic acid (122 mg, 1.0 mmol, 1.0 eq.) and LiCl (254 mg, 6.0 mmol, 6.0 eq.) were added in tetrahydrofuran. Subsequently, (2-ethylhexyl)sodium (0.2 M, 10 mL, 3.0 mmol, 3.0 eq.) was added. The crude product was purified by column chromatography to give the product (166 mg, 0.76 mmol, 76%) as a yellow liquid.

H NMR (300 MHz, $CDCl_3$): δ = 7.97-7.94 (m, 2H), 7.58-7.43 (m, 3H), 2.88-2.86 (m, 2H), 2.08-2.00 (m, 2H), 1.43-1.21 (m, 8H), 0.91-0.86 (m, 6H) ppm.

3-Ethyl-1-naphthalen-2-ylheptan-1-one (9b)

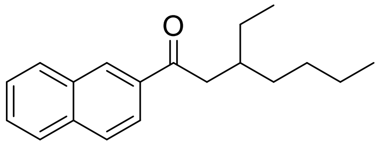

According to **GP 6**, 2-naphthoic acid (172 mg, 1.0 mmol, 1.0 eq.) and LiCl (254 mg, 6.0 mmol, 6.0 eq.) were added in tetrahydrofuran. Subsequently, (2-ethylhexyl)sodium (0.2 M, 10 mL, 3.0 mmol, 3.0 eq.) was added. The crude product was purified by column chromatography to give the product (180 mg, 0.67 mmol, 67%) as a colorless oil.

R$_f$ (SiO_2, dichloromethane) = 0.82.

^{1}H NMR (500 MHz, $CDCl_3$): δ = 8.47-8.46 (m, 1H), 8.03 (dd, *J* = 8.6, 1.8 Hz, 1H), 7.97 (d, *J* = 8.0 Hz, 1H), 7.89 (t, *J* = 8.6 Hz, 2H), 3.01 (dd, *J* = 6.7, 1.0 Hz, 2H), 2.11 (hept, *J* = 6.4 Hz, 1H), 1.49-1.23 (m, 9H), 0.94-0.88 (m, 5H) ppm.

^{13}C{^{1}H} NMR (125 MHz, $CDCl_3$): δ = 200.9, 135.6, 135.0, 132.7, 129.8, 129.7, 128.5, 128.4, 127.9, 126.8, 124.2, 43.3, 36.1, 33.5, 29.1, 26.7, 23.1, 14.2, 11.1 ppm.

IR: $\tilde{\nu}$ = 3059, 2959, 2929, 2857, 2360, 2337, 1680, 1629, 1596, 1467, 1374, 1277, 1262, 1182, 1174, 1120, 1020, 943, 862, 820, 746, 669 cm^{-1}.

HRMS (EI): calcd. for $C_{19}H_{24}O$ ($[M]^+$): m/z = 268.1827; found: m/z = 269.1899.

3-Ethyl-1-(3-methoxyphenyl)heptan-1-one (9c)

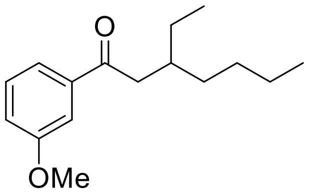

According to **GP 6**, 3-methoxybenzoic acid (152 mg, 1.0 mmol, 1.0 eq.) and LiCl (254 mg, 6.0 mmol, 6.0 eq.) were added in tetrahydrofuran. Subsequently, (2-ethylhexyl)sodium (0.2 M, 10 mL, 3.0 mmol, 3.0 eq.) was added. The crude product was purified by column chromatography to give the product (161 mg, 0.65 mmol, 65%) as a colorless oil.

R$_f$ (SiO_2, dichloromethane) = 0.74.

^{1}H NMR (500 MHz, $CDCl_3$): δ = 7.53-7.52 (m, 1H), 7.48 (m, 1H), 7.35 (t, *J* = 7.9 Hz, 1H), 7.10-7.08 (m, 1H), 3.85 (s, 3H), 2.85 (dd, *J* = 6.7, 1.6 Hz, 2H), 2.03 (hept, *J* = 6.3 Hz, 1H), 1.43-1.25 (m, 9H), 0.90-0.86 (m, 5H) ppm.

$^{13}C\{^1H\}$ **NMR** (125 MHz, $CDCl_3$): δ = 200.7, 159.9, 139.0, 129.6, 120.9, 119.4, 112.5, 55.5, 43.3, 35.9, 33.4, 29.0, 26.6, 23.1, 14.2, 11.0 ppm.

IR: $\tilde{\nu}$ = 2957, 2927, 2873, 2859, 1683, 1597, 1582, 1486, 1463, 1429, 1379, 1330, 1286, 1254, 1192, 1169, 1049, 1032, 994, 874, 863, 784, 750, 726, 686, 617, 567 cm^{-1}.

HRMS (EI): calcd. for $C_{16}H_{24}O_2$ ($[M]^+$): m/z = 248.1776; found: m/z = 249.1847.

3-Ethyl-1-(3-methylphenyl)heptan-1-one (9d)

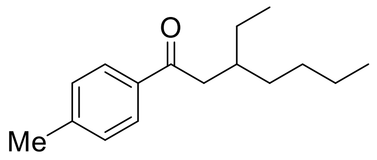

According to **GP 6**, *p*-toluic acid (136 mg, 1.0 mmol, 1.0 eq.) and LiCl (254 mg, 6.0 mmol, 6.0 eq.) were added in tetrahydrofuran. Subsequently, (2-ethylhexyl)sodium (0.2 M, 10 mL, 3.0 mmol, 3.0 eq.) was added. The crude product was purified by column chromatography (SiO_2, dichloromethane) to give the product (166 mg, 0.70 mmol, 71%) as a colorless oil.

H NMR (300 MHz, $CDCl_3$): δ = 7.78 (d, *J* = 8.2 Hz, 2H), 7.16 (d, *J* = 7.8 Hz, 2H), 2.76 (d, *J* = 6.7 Hz, 2H), 2.31 (s, 3H), 2.00-1.91 (m, 1H), 1.36-1.15 (m, 8H), 0.80 (t, *J* = 7.4 Hz, 6H) ppm.

3-Ethyl-1-(4-propan-2-ylphenyl)heptan-1-one (9e)

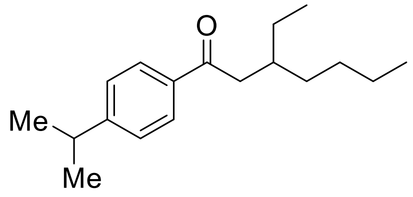

According to **GP 6**, 4-isopropylbenzoic acid (164 mg, 1.0 mmol, 1.0 eq.) and LiCl (254 mg, 6.0 mmol, 6.0 eq.) were added in tetrahydrofuran. Subsequently, (2-ethylhexyl)sodium (0.2 M, 10 mL, 3.0 mmol, 3.0 eq.) was added. The crude product was purified by column chromatography to give the product (157 mg, 0.60 mmol, 60%) as a colorless oil.

R_f (SiO_2, dichloromethane) = 0.79.

^1H **NMR** (300 MHz, $CDCl_3$): δ = 7.89 (d, *J* = 8.2 Hz, 2H), 7.31 (d, *J* = 8.3 Hz, 2H), 2.96 (p, *J* = 6.9 Hz, 1H), 2.84 (d, *J* = 6.7 Hz, 2H), 2.04 (dt, *J* = 12.4, 6.0 Hz, 1H), 1.36-1.14 (m, 14H), 0.95-0.81 (m, 6H) ppm.

$^{13}C\{^1H\}$ NMR (75 MHz, $CDCl_3$): δ = 200.6, 154.4, 135.5, 128.5, 126.8, 43.1, 35.9, 34.4, 33.4, 29.0, 26.6, 23.8, 23.1, 14.3, 11.1 ppm.

IR: $\tilde{\nu}$ = 2960, 2928, 2873, 1682, 1607, 1462, 1414, 1380, 1364, 1281, 1260, 1218, 1184, 1098, 1055, 1012, 821, 805, 728, 581 cm^{-1}.

HRMS (EI): calcd. for $C_{18}H_{28}O$ ($[M]^+$): m/z = 261.2213; found: m/z = 261.2211.

3-Ethyl-1-[4-(trifluoromethyl)phenyl]heptan-1-one (9f)

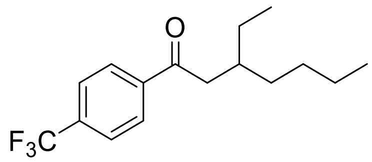

According to **GP 6**, 4-trifluoromethylbenzoic acid (190 mg, 1.0 mmol, 1.0 eq.) and LiCl (254 mg, 6.0 mmol, 6.0 eq.) were added in tetrahydrofuran. Subsequently, (2-ethylhexyl)sodium (0.2 M, 10 mL, 3.0 mmol, 3.0 eq.) was added. The crude product was purified by column chromatography (SiO_2, dichloromethane) to give the product (162 mg, 0.57 mmol, 57%) as a yellow liquid.

H NMR (300 MHz, $CDCl_3$): δ = 8.05 (d, *J* = 8.2 Hz, 2H), 7.72 (d, *J* = 8.2 Hz, 2H), 2.89 (d, *J* = 6.6 Hz, 1H), 2.08-1.97 (m, 2H), 1.47-1.23 (m, 8H), 0.94-0.84 (m, 6H) ppm.

1-Cyclohexyl-3-ethylheptan-1-one (9g)

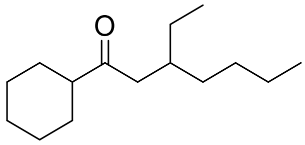

According to **GP 6**, cyclohexanecarboxylic acid (128 mg, 1.0 mmol, 1.0 eq.) and LiCl (254 mg, 6.0 mmol, 6.0 eq.) were added in tetrahydrofuran. Subsequently, (2-ethylhexyl)sodium (0.2 M, 10 mL, 3.0 mmol, 3.0 eq.) was added. The crude product was purified by column chromatography (SiO_2, dichloromethane) to give the product (153 mg, 0.68 mmol, 68%) as a yellow liquid.

^1H NMR (300 MHz, $CDCl_3$): δ = 2.36-2.25 (m, 3H), 1.89-1.61 (m, 6H), 1.37-1.13 (m, 13H), 0.89-0.77 (m, 6H) ppm.

3-Ethyl-1-pyridin-2-ylheptan-1-one (9h)

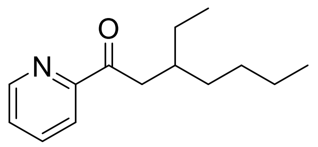

According to **GP 6**, picolinic acid (123 mg, 1.0 mmol, 1.0 eq.) and LiCl (254 mg, 6.0 mmol, 6.0 eq.) were added in tetrahydrofuran. Subsequently, (2-ethylhexyl)sodium (0.2 M, 10 mL, 3.0 mmol, 3.0 eq.) was added. The crude product was purified by column chromatography to give the product (72 mg, 0.33 mmol, 33%) as a colorless oil.

R$_f$ (SiO_2, dichloromethane) = 0.65.

^{1}H NMR (500 MHz, $CDCl_3$): δ = 8.67 (ddd, *J* = 4.8, 1.8, 0.9 Hz, 1H), 8.03 (dt, *J* = 7.9, 1.1 Hz, 1H), 7.82 (td, *J* = 7.7, 1.7 Hz, 1H), 7.45 (ddd, *J* = 7.5, 4.7, 1.3 Hz, 1H), 3.15 (dd, *J* = 6.7, 1.5 Hz, 2H), 2.06 (hept, *J* = 6.3 Hz, 1H), 1.43-1.26 (m, 9H), 0.90-0.85 (m, 5H) ppm.

^{13}C{^{1}H} NMR (125 MHz, $CDCl_3$): δ = 202.5, 154.0, 149.0, 137.0, 127.0, 121.9, 42.0, 35.4, 33.5, 29.1, 26.7, 23.1, 14.2, 11.1 ppm.

IR: $\tilde{\nu}$ = 2959, 2927, 2873, 2859, 2360, 2340, 1694, 1684, 1569, 1464, 1436, 1399, 1379, 1309, 1286, 1242, 1220, 1214, 1089, 1019, 994, 799, 766, 740, 680, 619, 603 cm^{-1}.

HRMS (EI): calcd. for $C_{14}H_{21}NO$ ($[M]^+$): m/z = 219.1623; found: m/z = 220.1695.

6. Reaction under the Optimized Conditions of Benzoic Acid with *n*-Butyllithium

1-Phenylpentan-1-one (10)

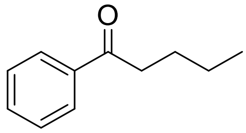

In an oven-dried 50 ml Schlenk flask, benzoic acid (122 mg, 1.0 mmol, 1.0 eq.) and LiCl (254 mg, 6.0 mmol, 6.0 eq.) was charged in anhydrous tetrahydrofuran under a nitrogen atmosphere. Subsequently, *n*-butyllithium (2.5 M, 1.2 mL, 3.0 mmol, 3.0 eq. was slowly added to the reaction solution. The reaction solution was treated in an ultrasonic bath for 30 minutes and then stirred overnight at room temperature. The solvent was removed under reduced pressure. The crude product was purified by column chromatography so that the product (58 mg, 0.4 mmol, 36%) could be obtained as a colorless liquid.

R_f (SiO_2, dichlormethane) = 0.71.

^{1}H NMR (300 MHz, $CDCl_3$): δ = 7.97-7.95 (m, 2H), 7.57-7.53 (m, 1H), 7.47-7.44 (m, 2H), 2.98-2.95 (m, 2H), 1.73 (p, *J* = 7.5 Hz, 2H), 1.46-1.37 (m, 2H), 0.96 (t, *J* = 7.3 Hz, 3H) ppm.

^{13}C{^{1}H} NMR (75 MHz, $CDCl_3$): δ = 200.7, 137.4, 133.0, 128.7, 128.21, 38.5, 26.9, 22.7, 14.1 ppm.

The analytical data are in accordance with the literature.[22]

7. ^{1}H and ^{13}C NMR Spectra of the Substrate and all Products

3-(Chloromethyl)heptane (1)

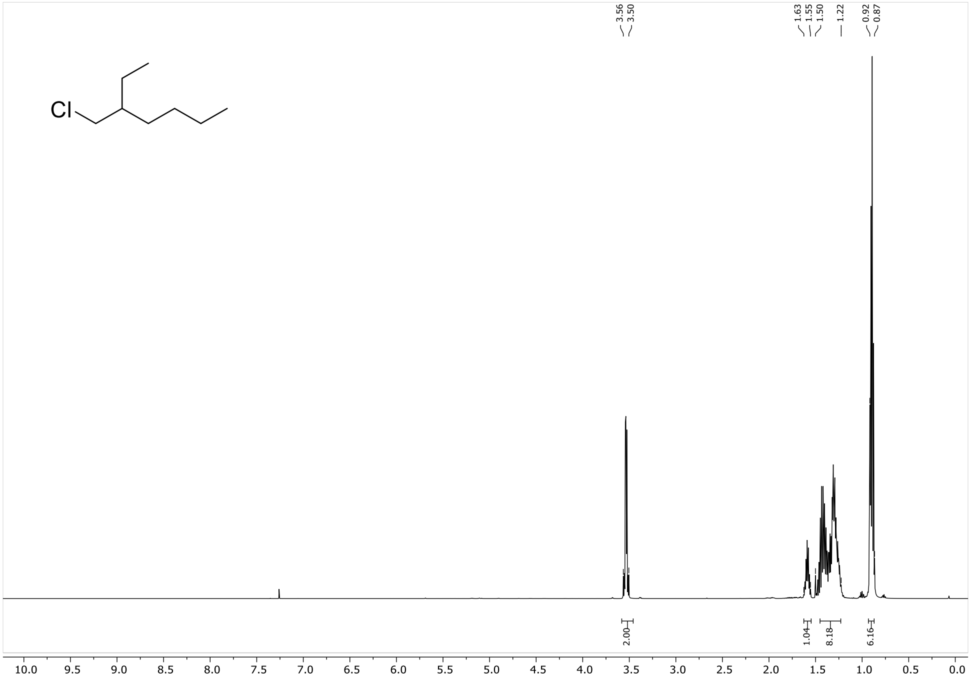

^{1}H NMR (500 MHz, $CDCl_3$)

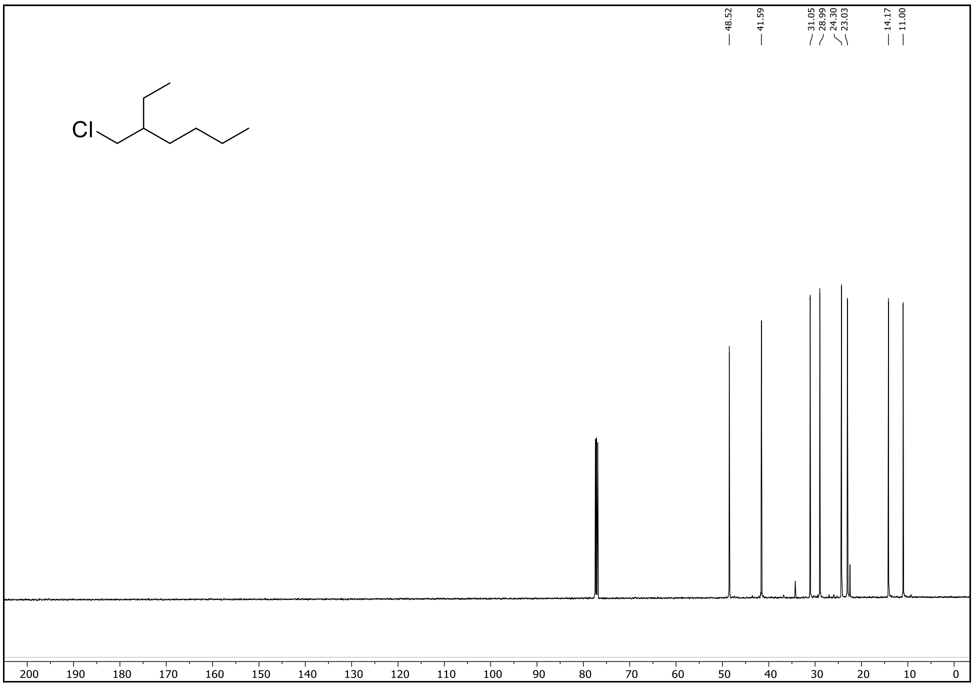

^{13}C{^{1}H} NMR (125 MHz, $CDCl_3$)

N-Methoxy-*N*-methyl-4-trifluoromethylbenzamide (3b)

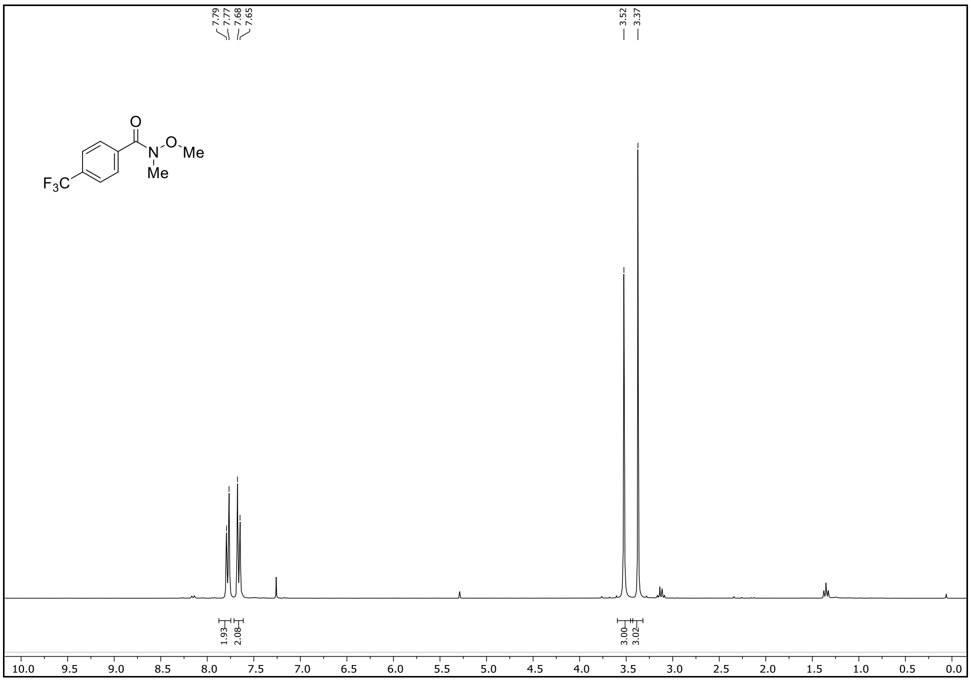

^{1}H NMR (300 MHz, $CDCl_3$)

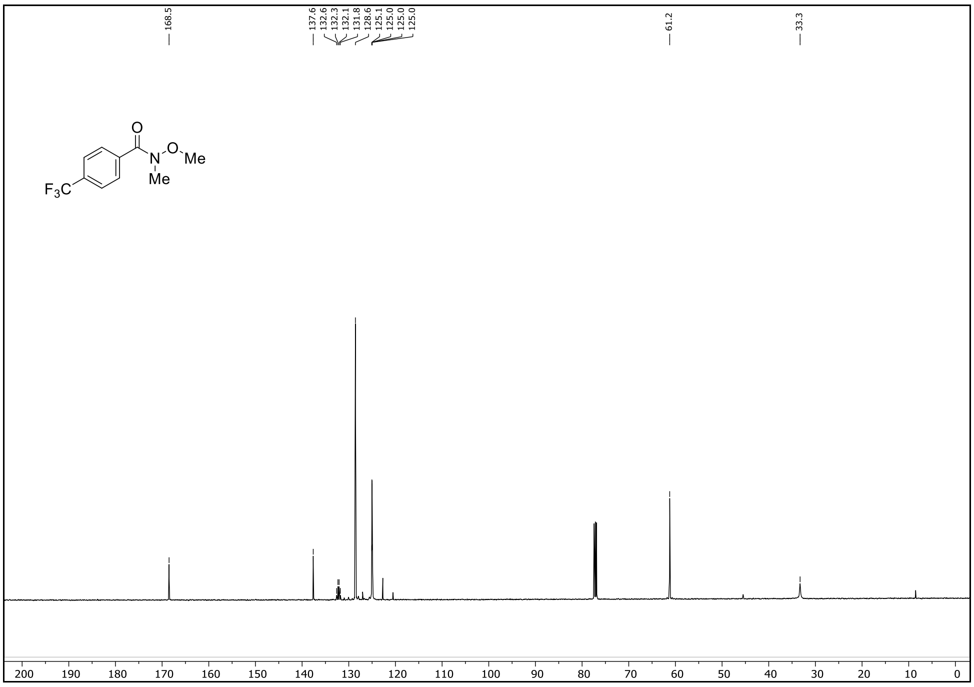

$^{13}C\{^1H\}$ NMR (125 MHz, $CDCl_3$)

***N*-Methoxy-*N*,4-dimethylbenzamide (3c)**

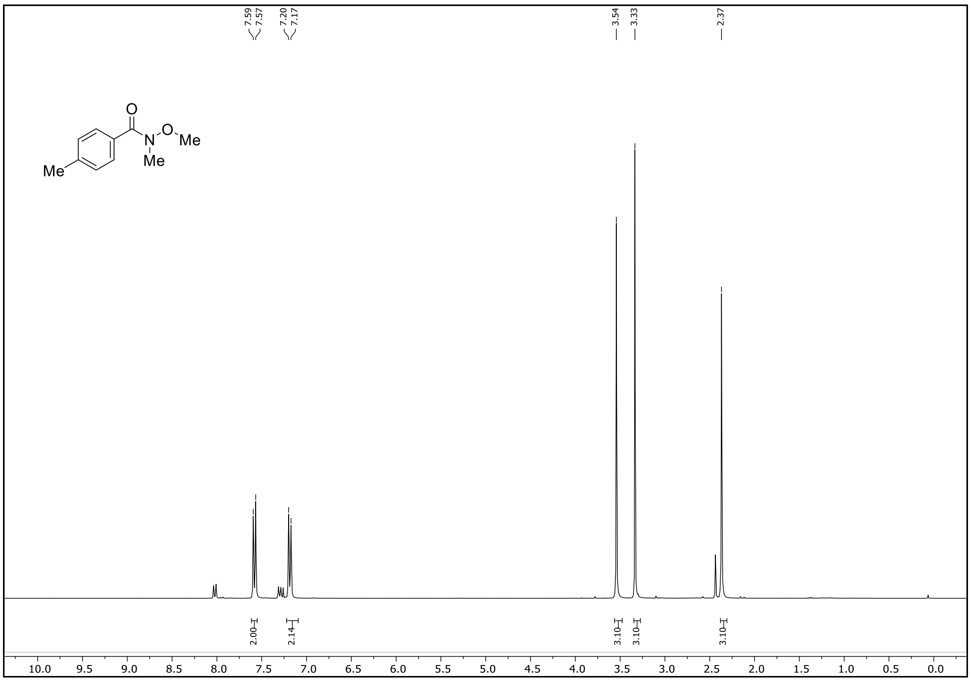

^{1}H NMR (300 MHz, $CDCl_3$)

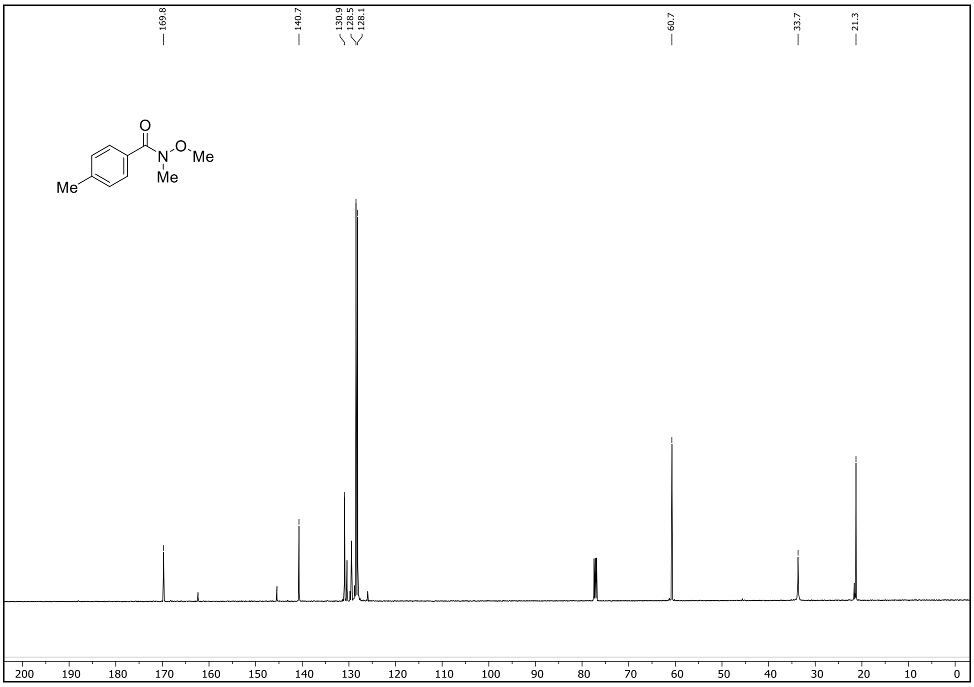

^{13}C{^{1}H} NMR (125 MHz, $CDCl_3$)

4,*N*-Dimethoxy-*N*-methylbenzamide (3d)

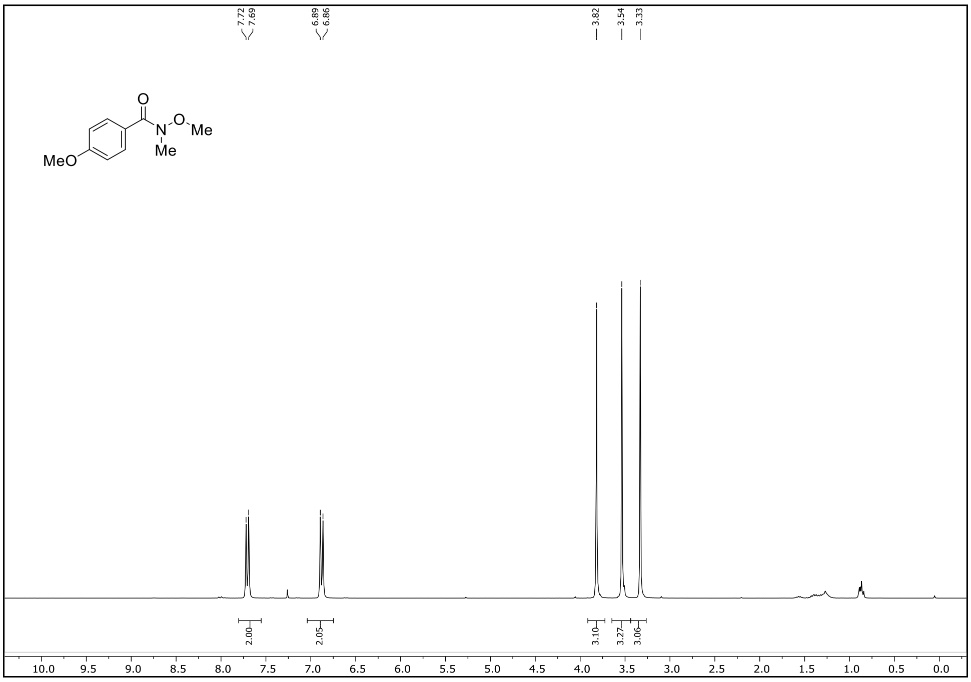

^{1}H NMR (300 MHz, $CDCl_3$)

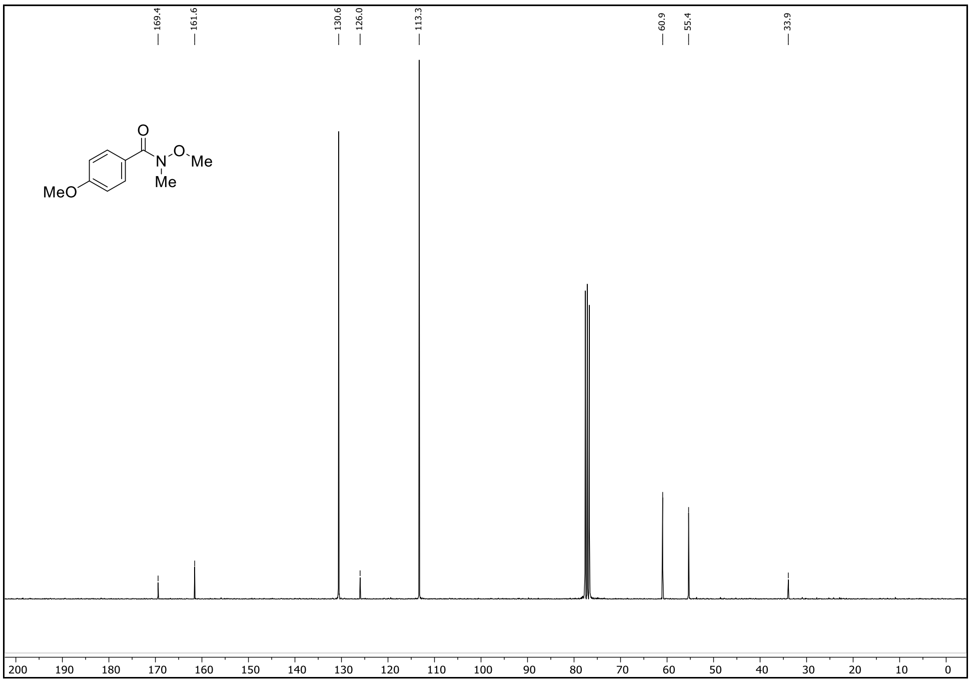

$^{13}C\{^1H\}$ NMR (75 MHz, $CDCl_3$)

N-Methoxy-N-methylthiophene-2-carboxamid (3f)

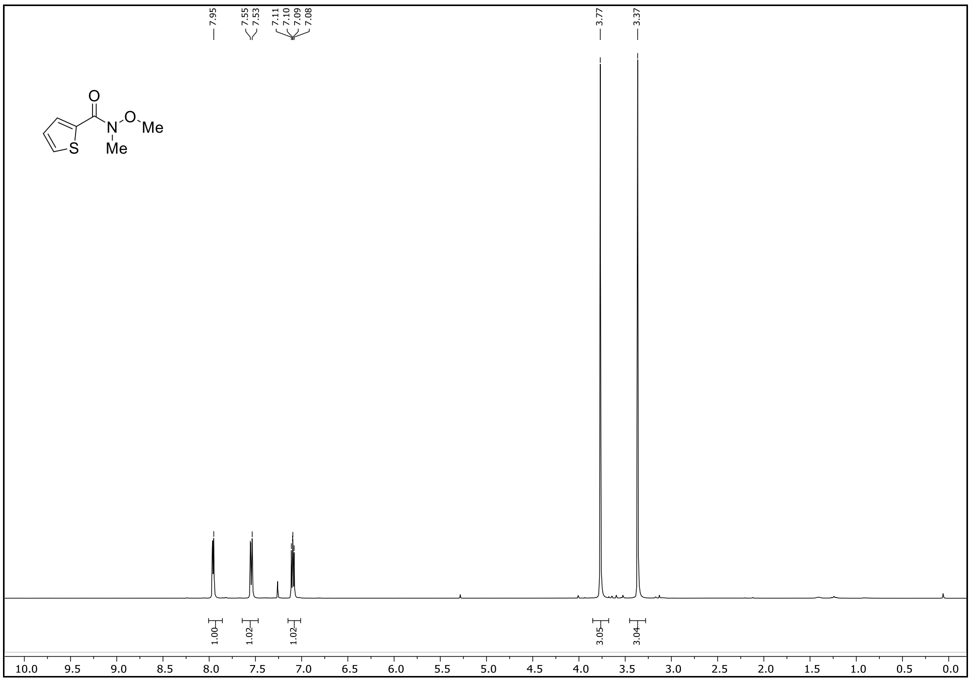

^{1}H NMR (300 MHz, $CDCl_3$)

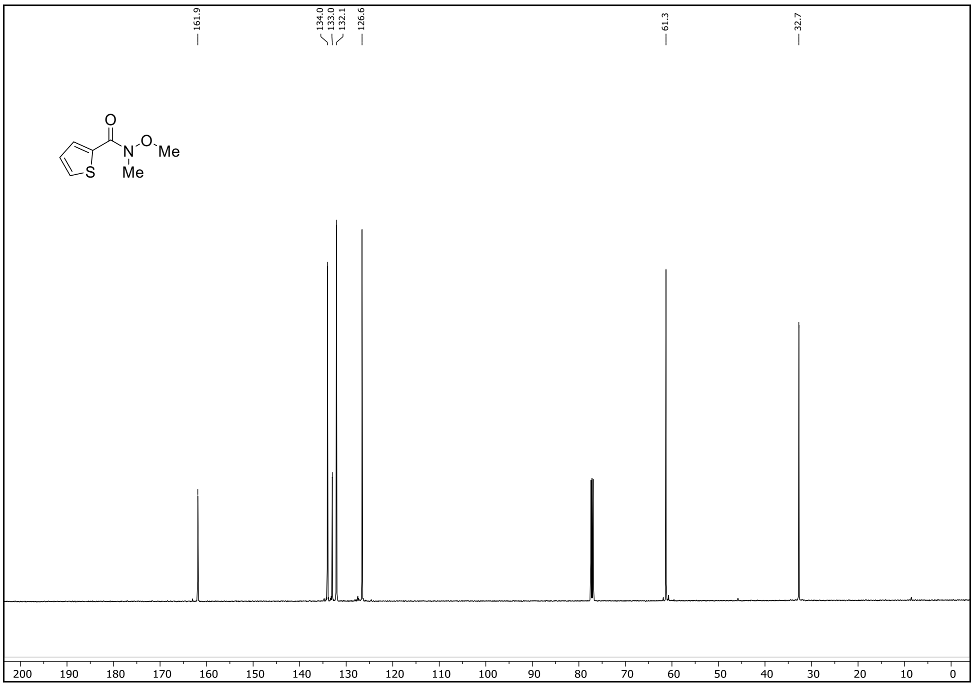

^{13}C{^{1}H} NMR (125 MHz, $CDCl_3$)

_N_-Methoxy-_N_-methylcyclohexanecarboxamide (3h)

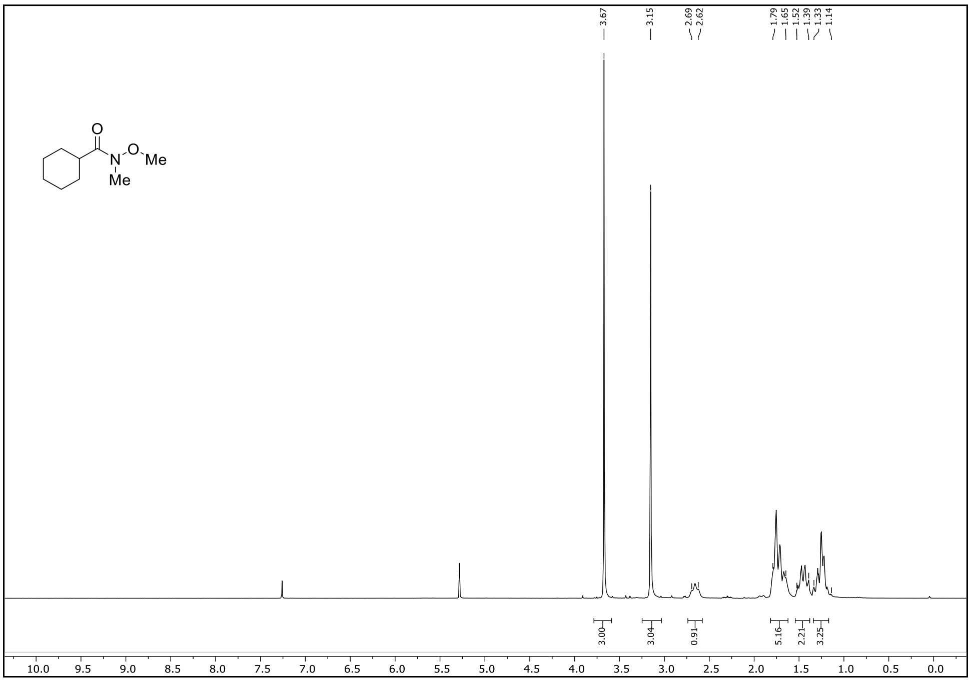

^{1}H NMR (300 MHz, $CDCl_3$)

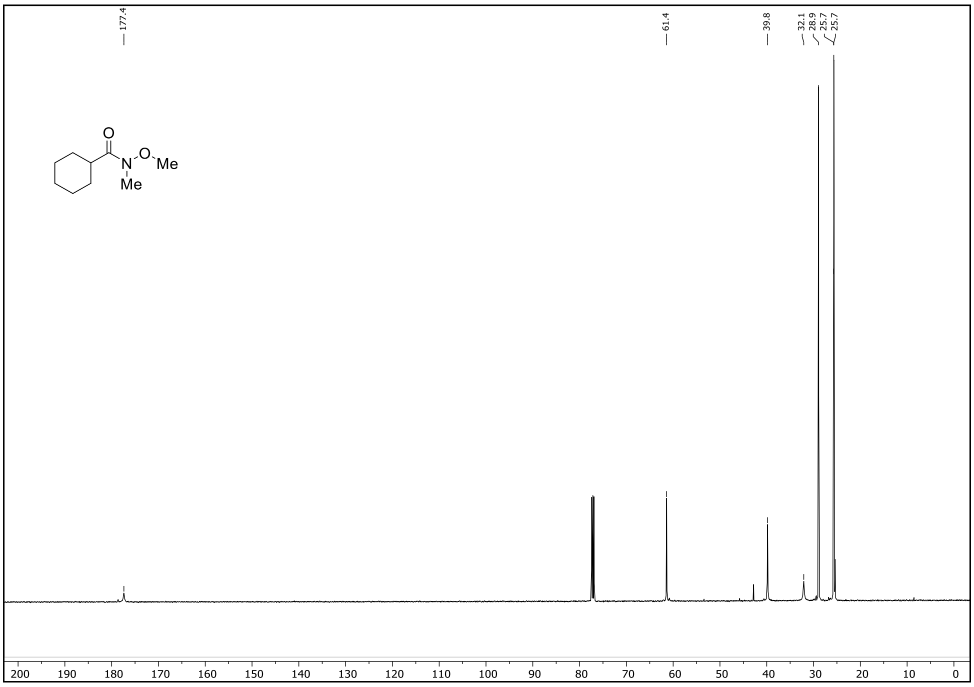

$^{13}C\{^1H\}$ NMR (125 MHz, $CDCl_3$)

_N_-Methoxy-_N_-methyloctanamide (3i)

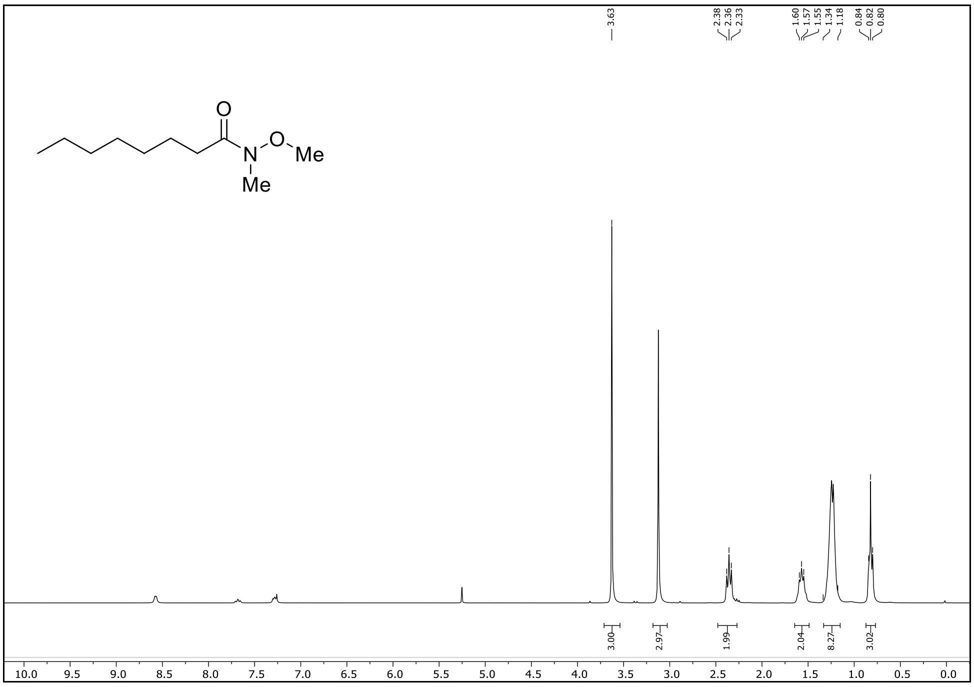

^{1}H NMR (300 MHz, $CDCl_3$)

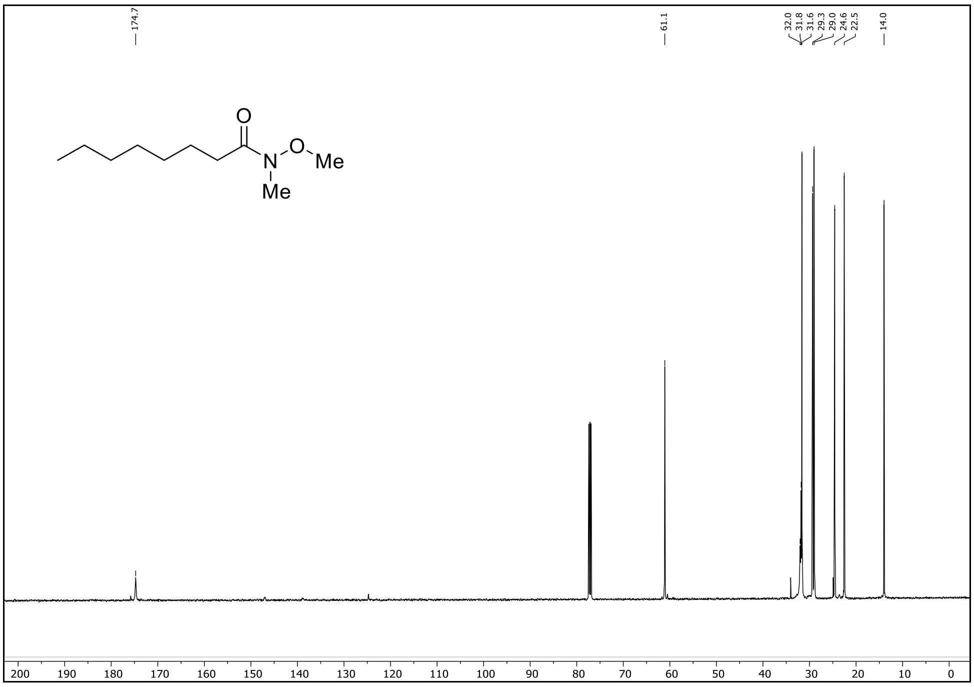

^{13}C{^{1}H} NMR (125 MHz, $CDCl_3$)

3-Ethyl-1-phenylheptan-1-one (4a)

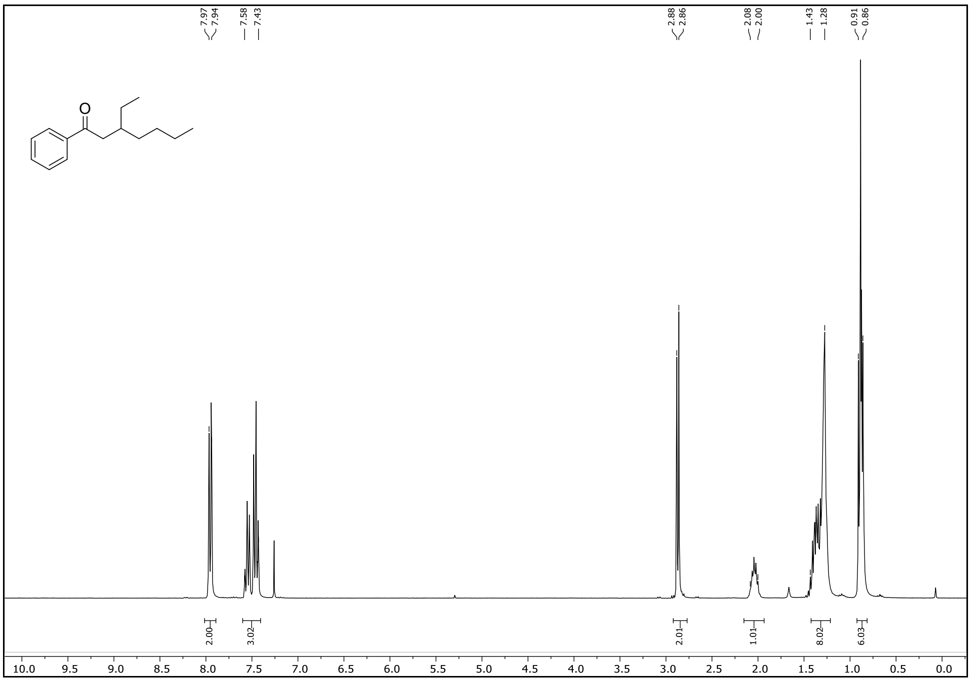

^{1}H NMR (300 MHz, $CDCl_3$)

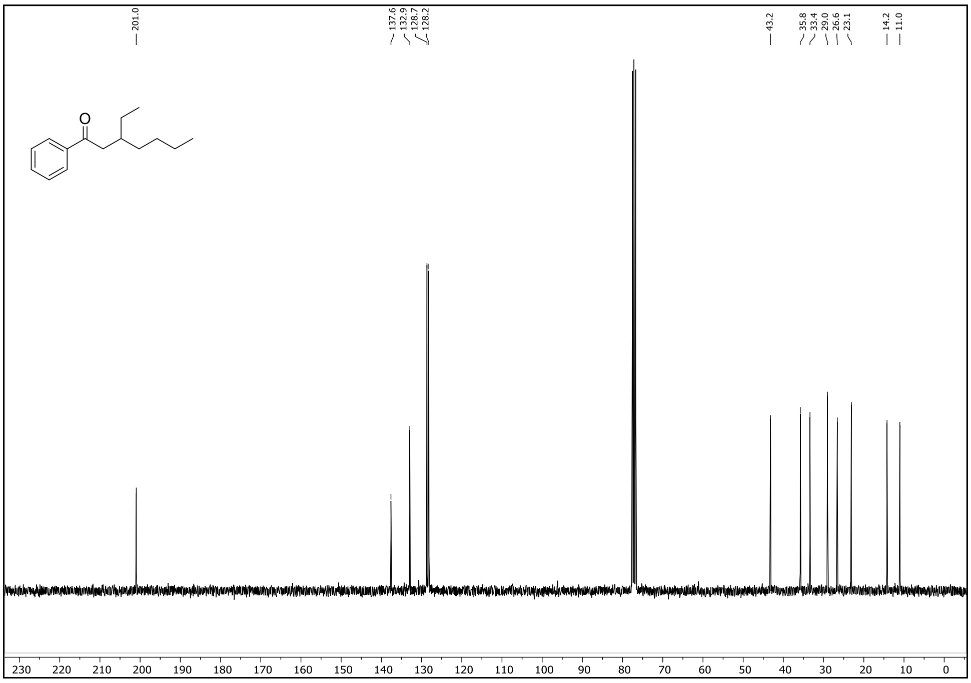

$^{13}C\{^1H\}$ NMR (75 MHz, $CDCl_3$)

3-Ethyl-1-[4-(trifluoromethyl)phenyl]heptan-1-one (4b)

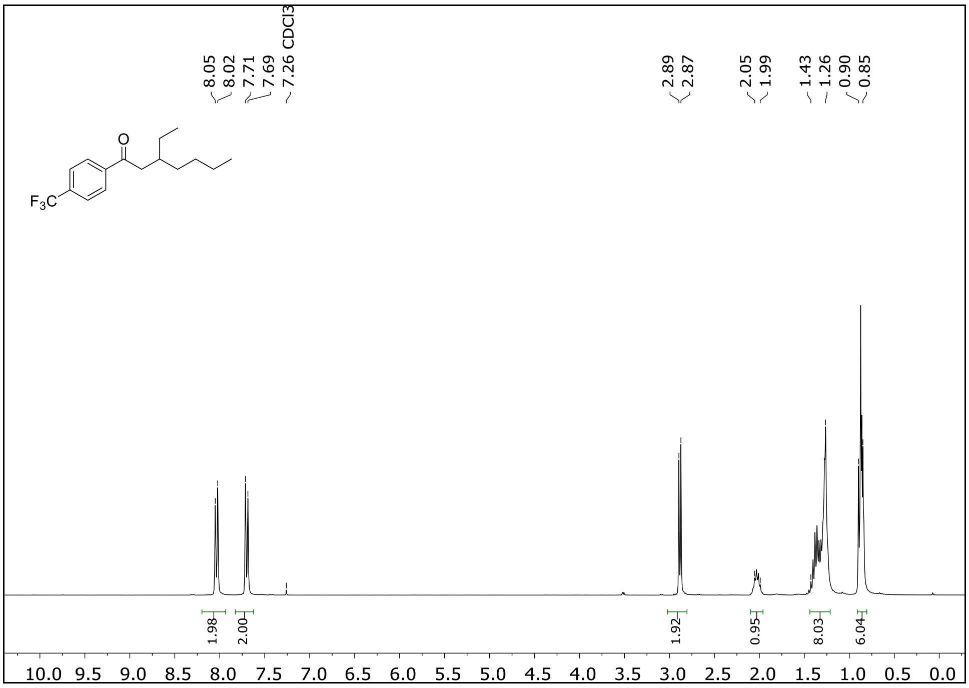

^{1}H NMR (300 MHz, $CDCl_3$)

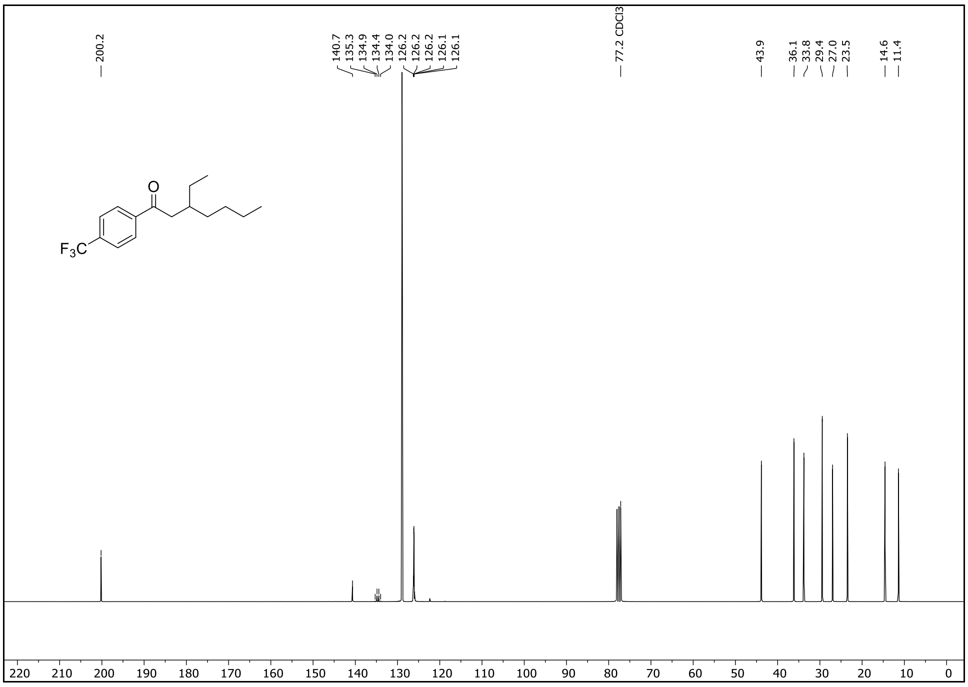

$^{13}C\{^{1}H\}$ NMR (75 MHz, $CDCl_3$)

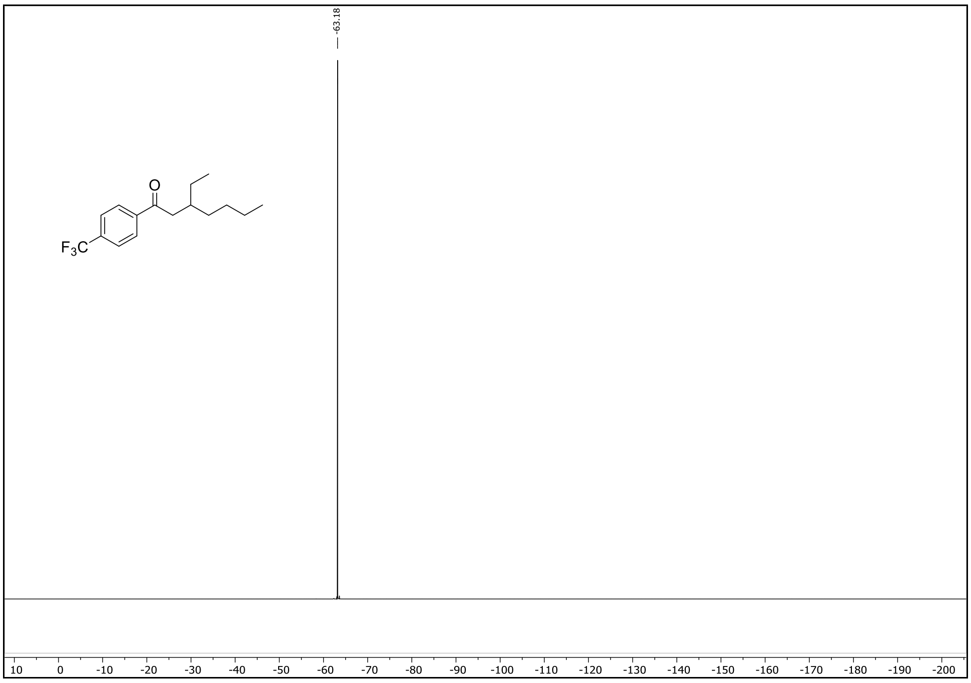

^{19}F NMR (470 MHz, $CDCl_3$)

3-Ethyl-1-(*p*-tolyl)heptan-1-one (4c)

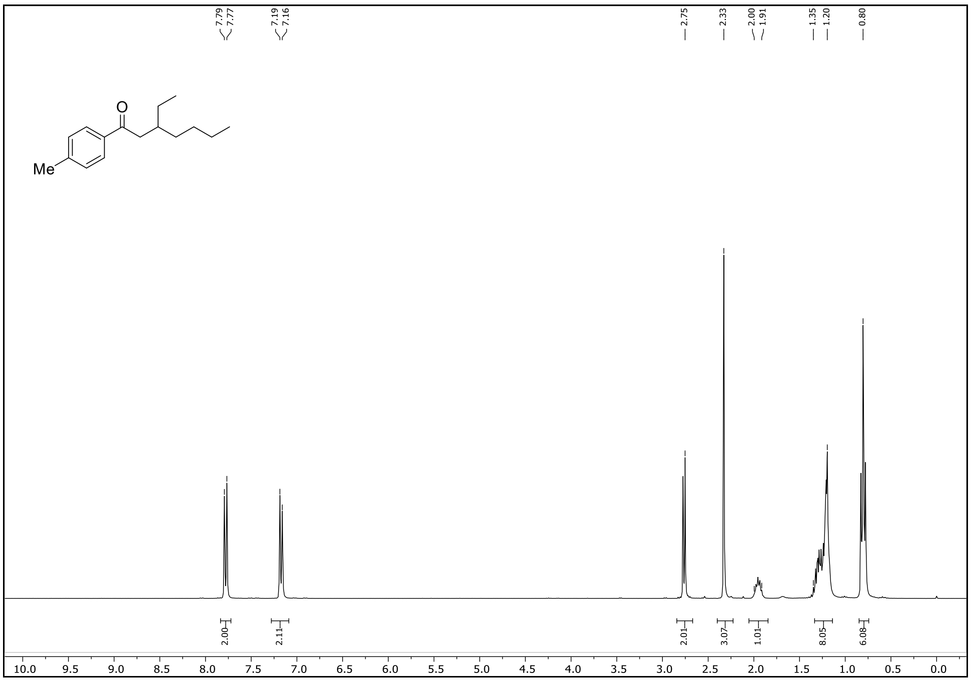

^{1}H NMR (300 MHz, $CDCl_3$)

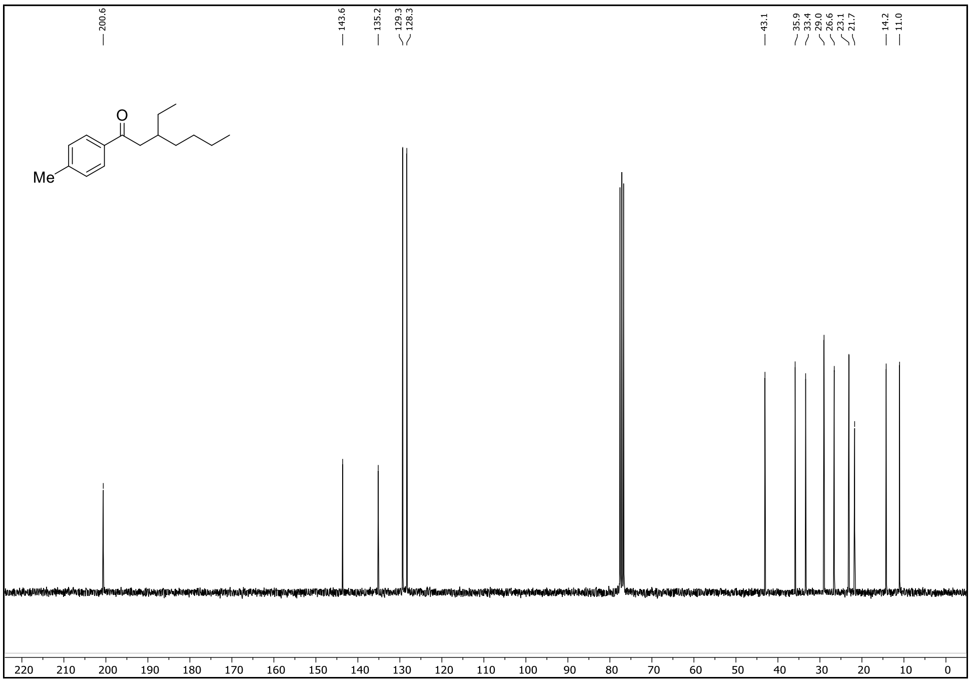

$^{13}C\{^{1}H\}$ NMR (75 MHz, $CDCl_3$)

3-Ethyl-1-(4-methoxyphenyl)heptan-1-one (4d)

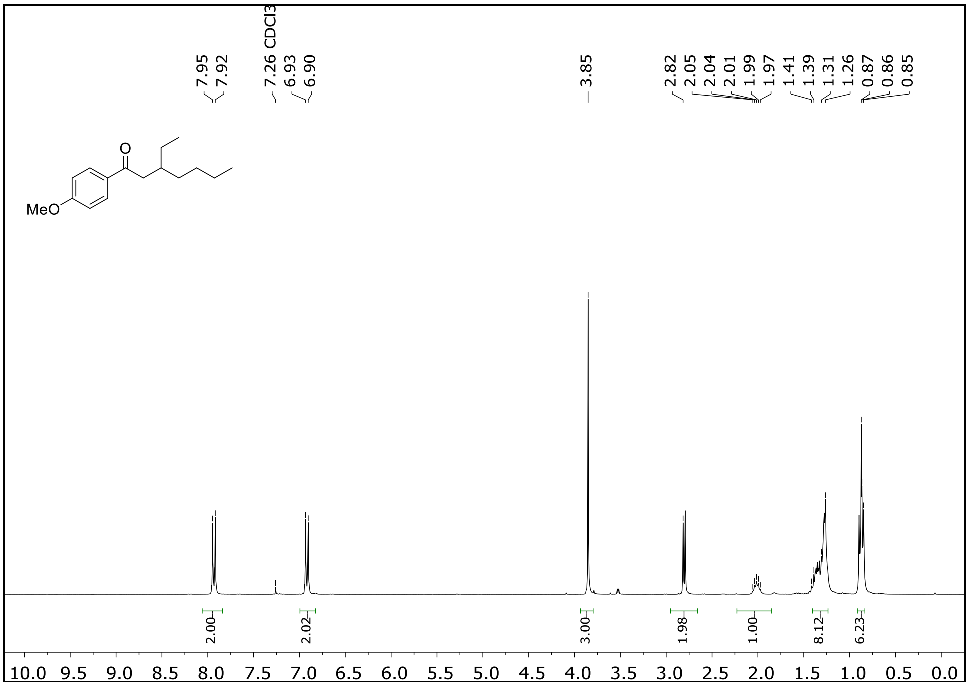

^{1}H NMR (300 MHz, $CDCl_3$)

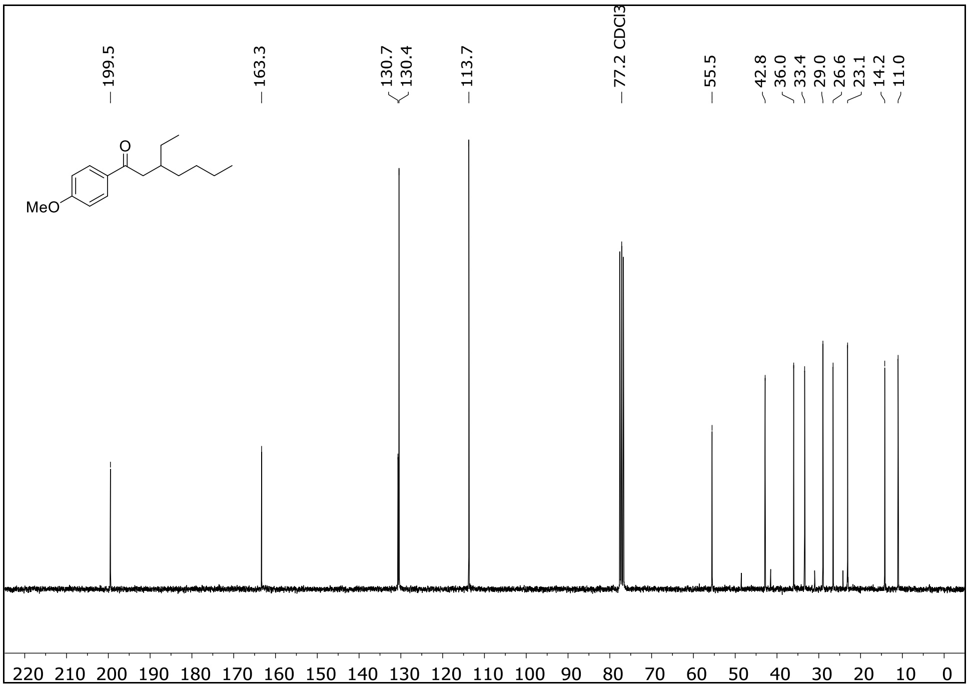

$^{13}C\{^{1}H\}$ NMR (75 MHz, $CDCl_3$)

4-(3-Ethylheptanoyl)benzonitrile (4e)

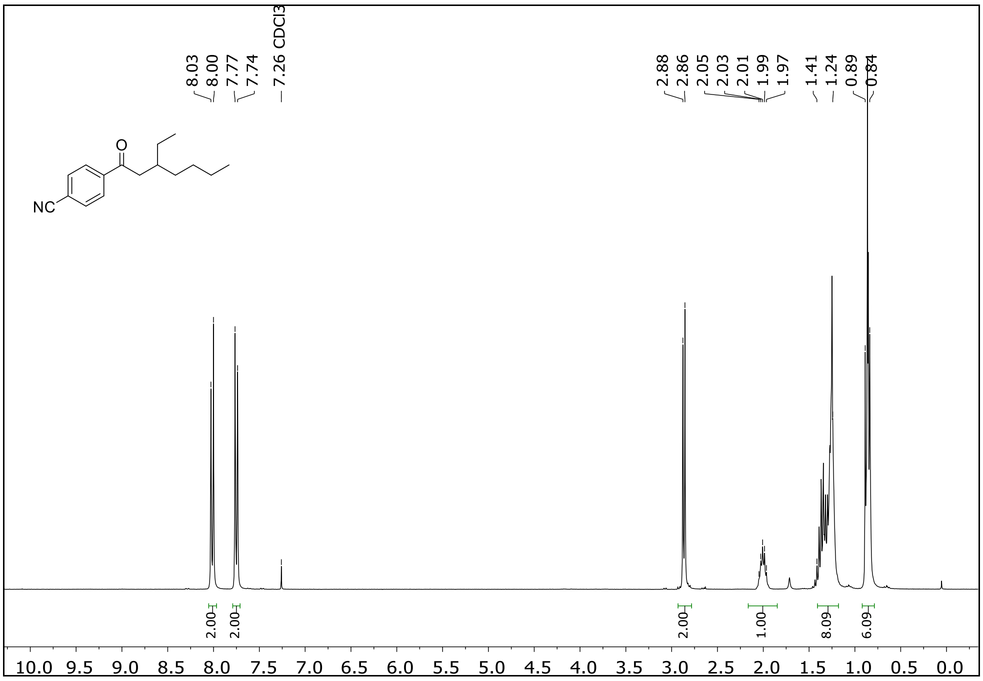

^{1}H NMR (300 MHz, $CDCl_3$)

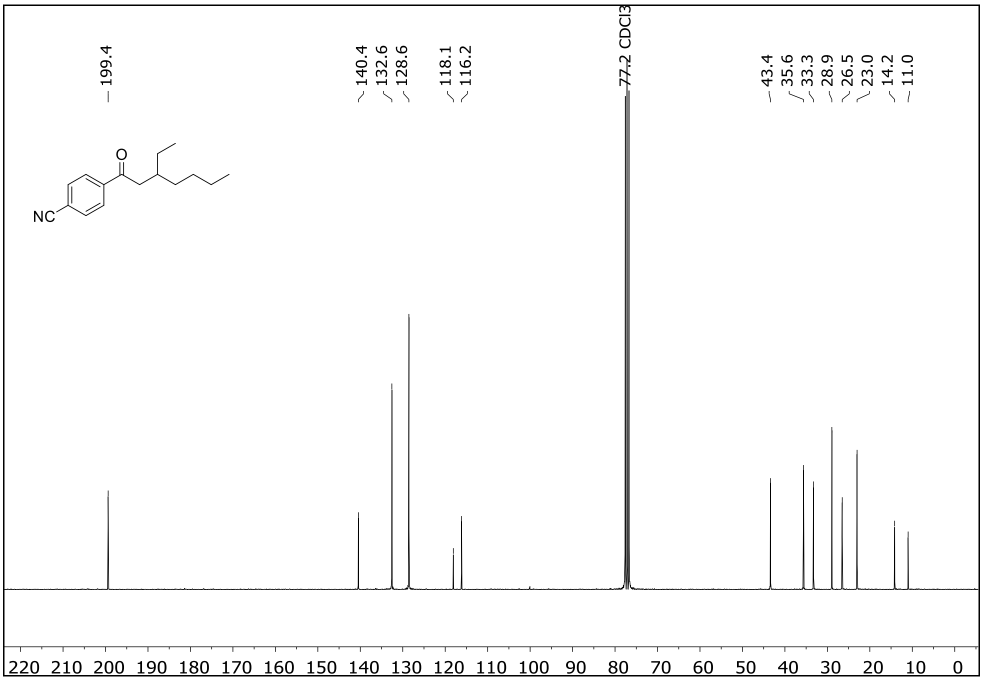

$^{13}C\{^{1}H\}$ NMR (75 MHz, $CDCl_3$)

3-Ethyl-1-thiophen-2-ylheptan-1-one (4f)

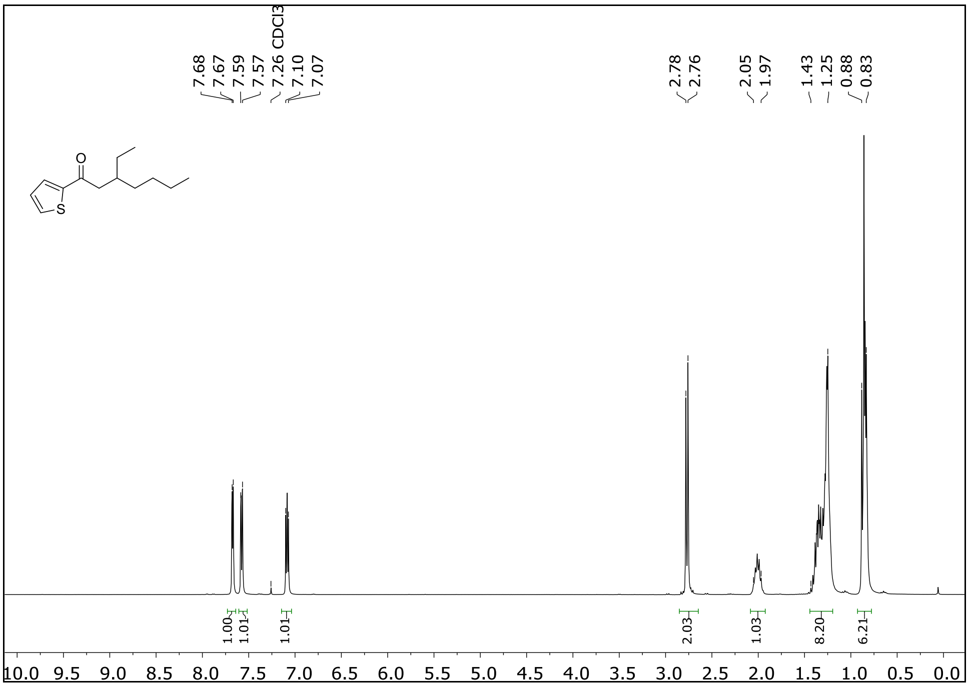

^{1}H NMR (300 MHz, $CDCl_3$)

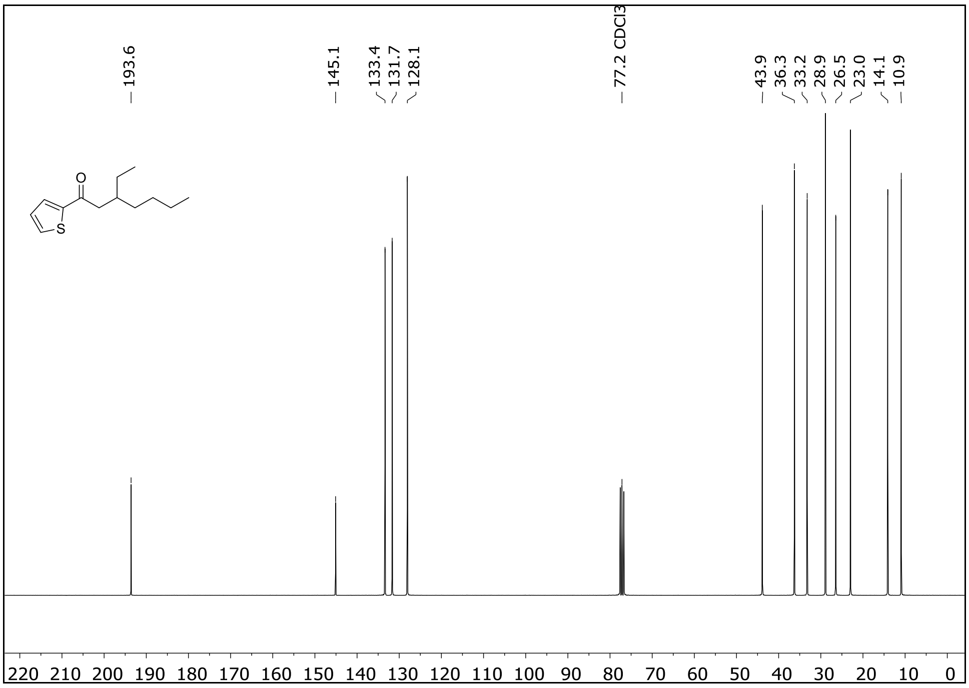

$^{13}C\{^1H\}$ NMR (75 MHz, $CDCl_3$)

3-Ethyl-1-pyridin-3-ylheptan-1-one (4g)

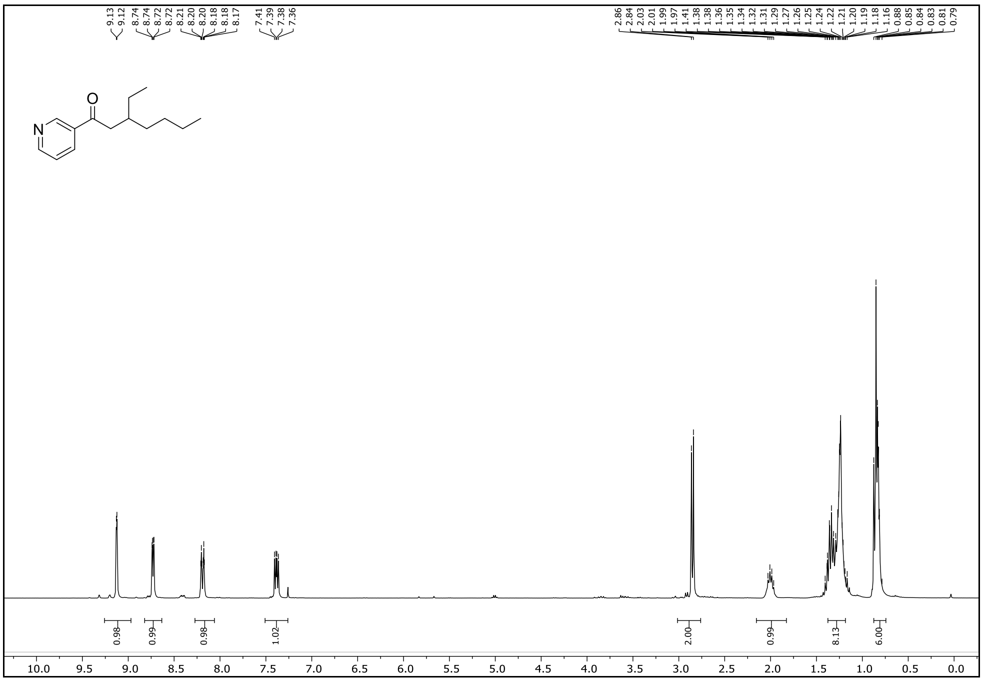

^1H NMR (300 MHz, $CDCl_3$)

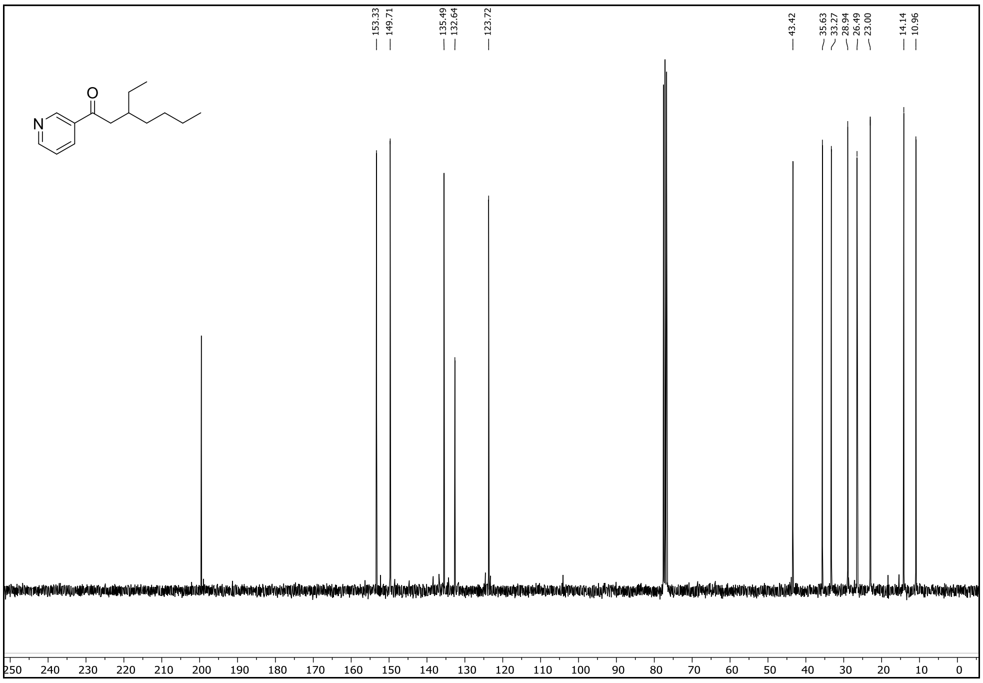

$^{13}C\{^1H\}$ NMR (75 MHz, $CDCl_3$)

1-Cyclohexyl-3-ethylheptan-1-one (4h)

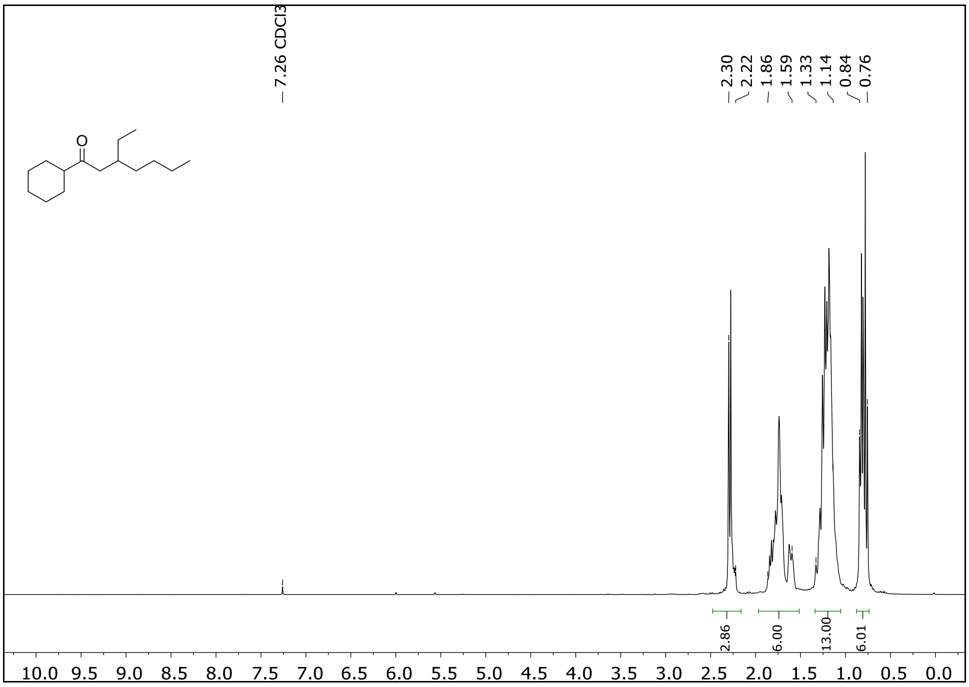

^1H NMR (300 MHz, $CDCl_3$)

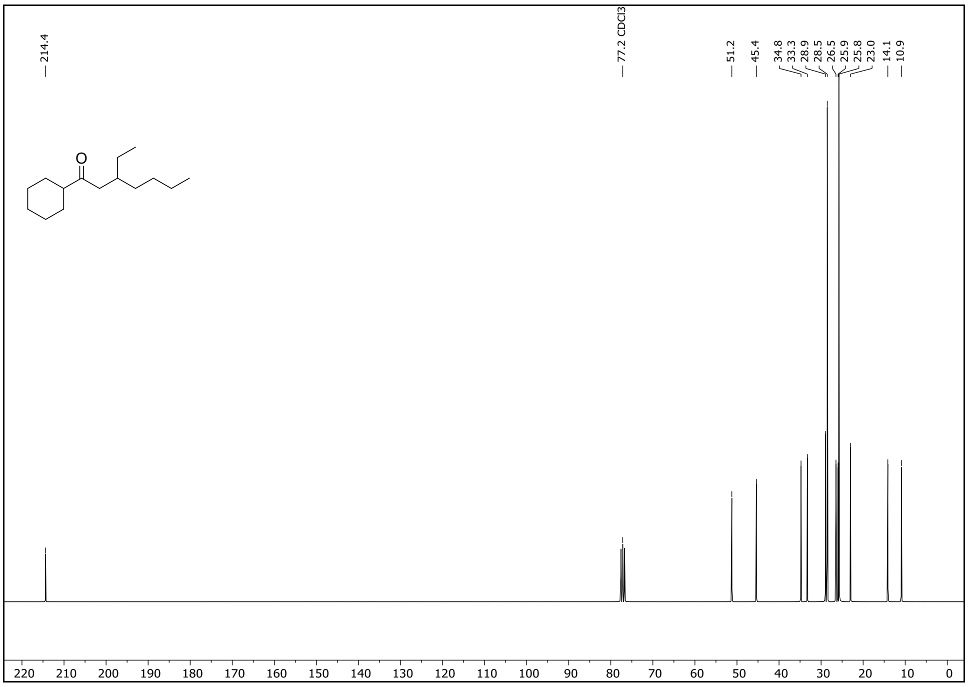

$^{13}C\{^1H\}$ NMR (75 MHz, $CDCl_3$)

***N,N*-Dimethyl-4-ethylaniline (5k)**

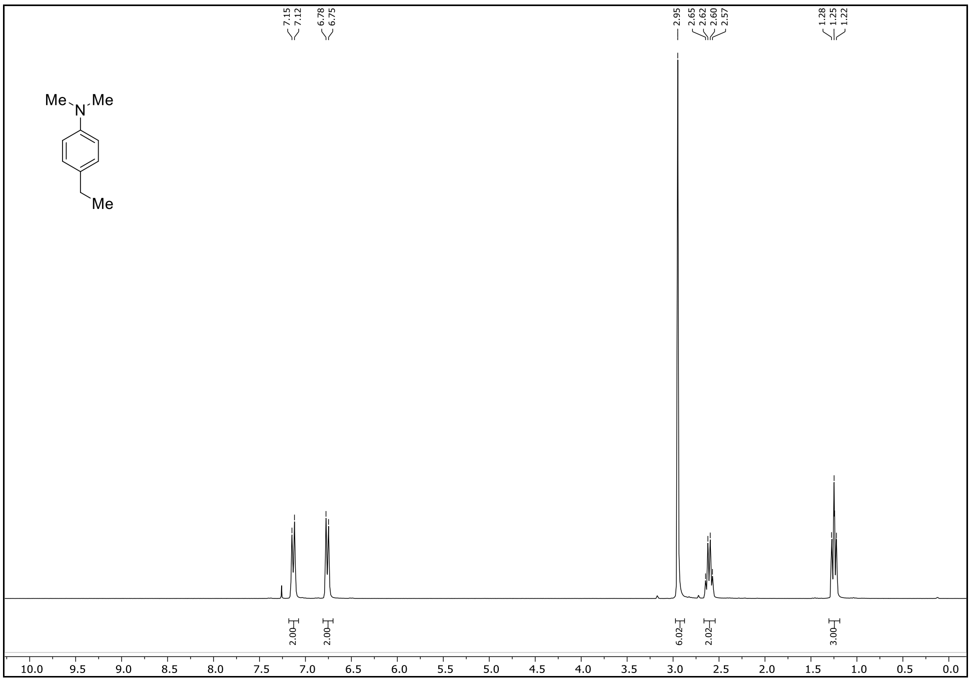

^1H NMR (300 MHz, $CDCl_3$)

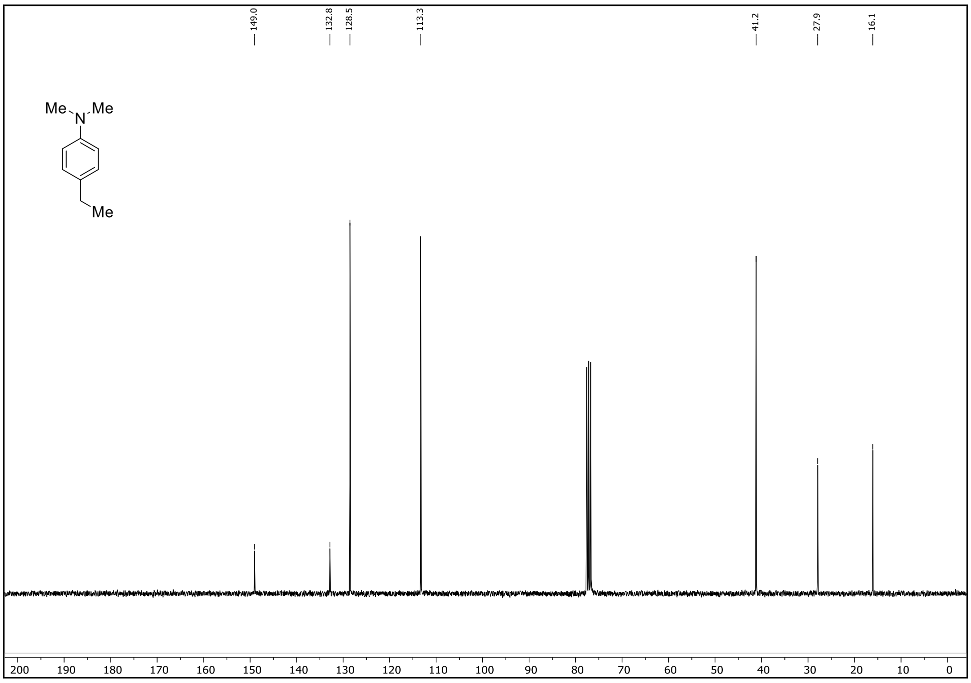

$^{13}C\{^{1}H\}$ NMR (75 MHz, $CDCl_3$)

4-Ethyl-*N*-methylaniline (5j)

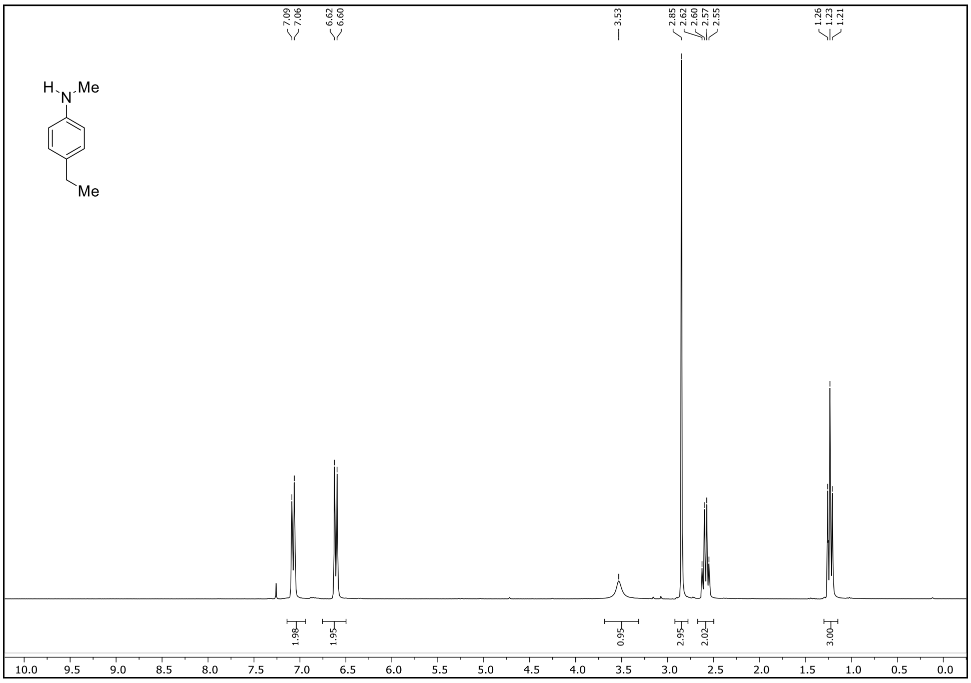

^{1}H NMR (300 MHz, $CDCl_3$)

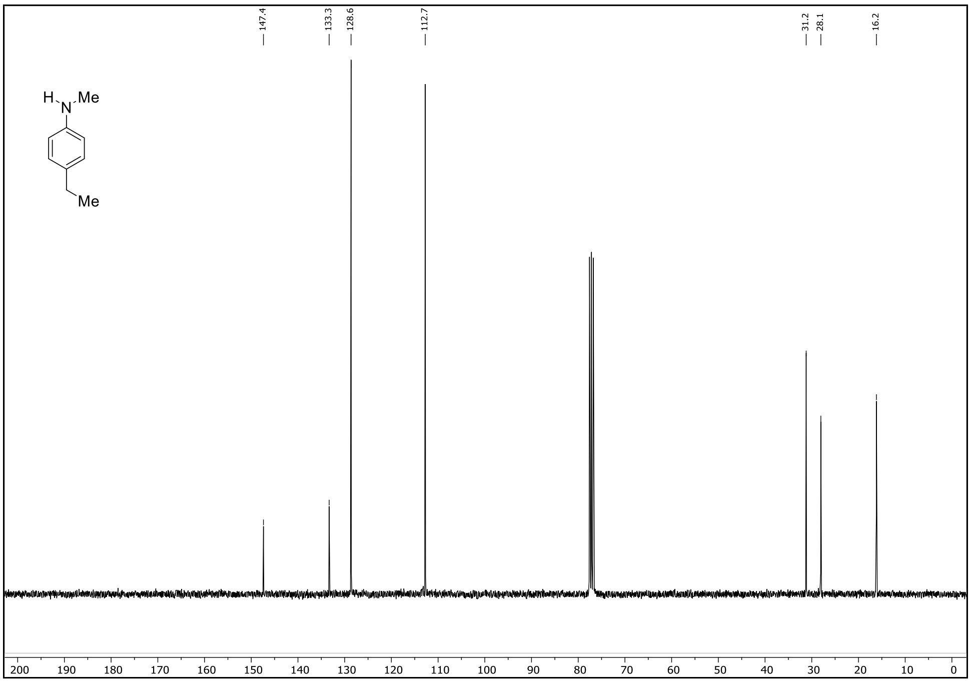

$^{13}C\{^{1}H\}$ NMR (75 MHz, $CDCl_3$)

2-Ethylanisole (5m)

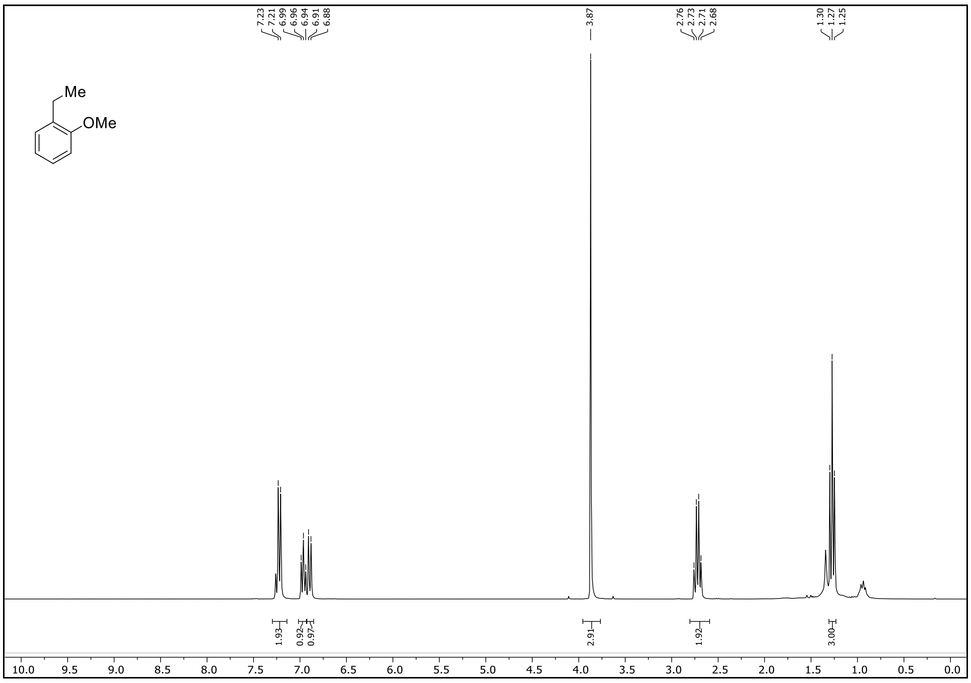

^{1}H NMR (300 MHz, $CDCl_3$)

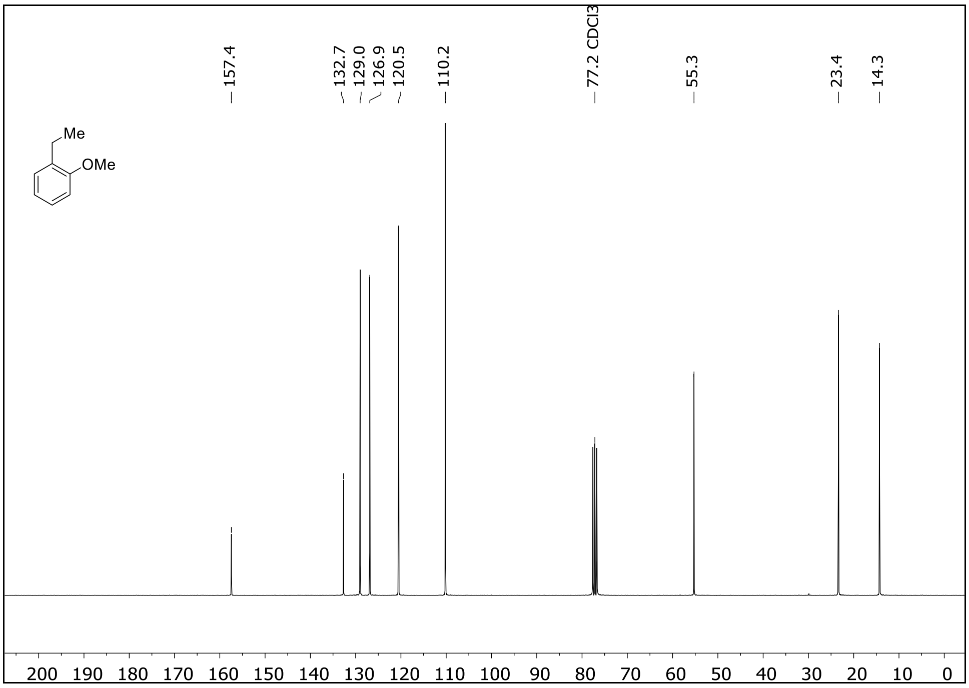

$^{13}C\{^{1}H\}$ NMR (75 MHz, $CDCl_3$)

3-Ethylanisole (5n)

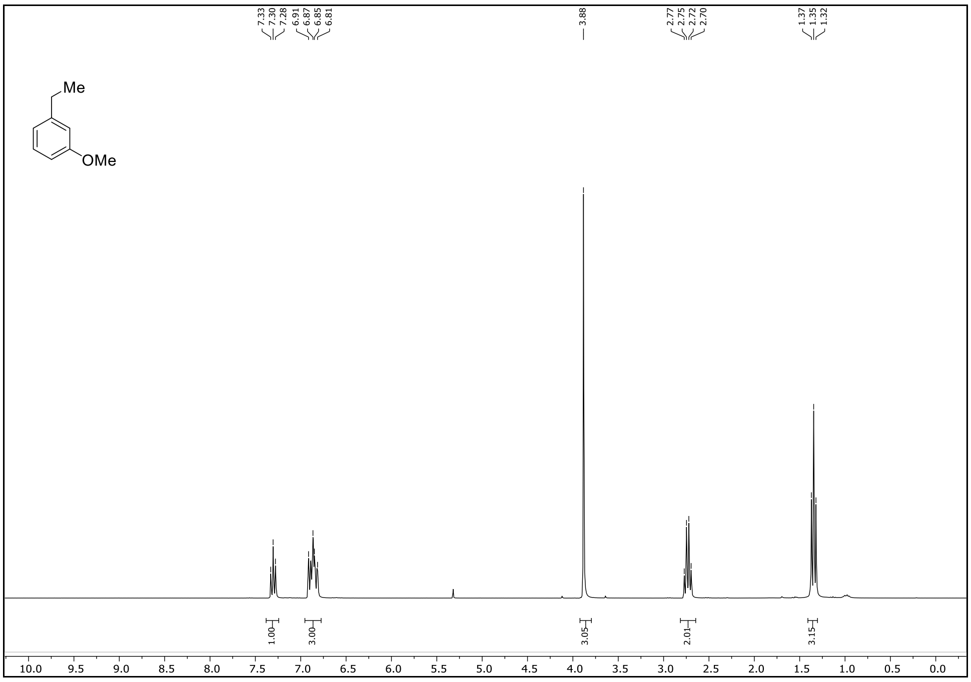

^{1}H NMR (300 MHz, $CDCl_3$)

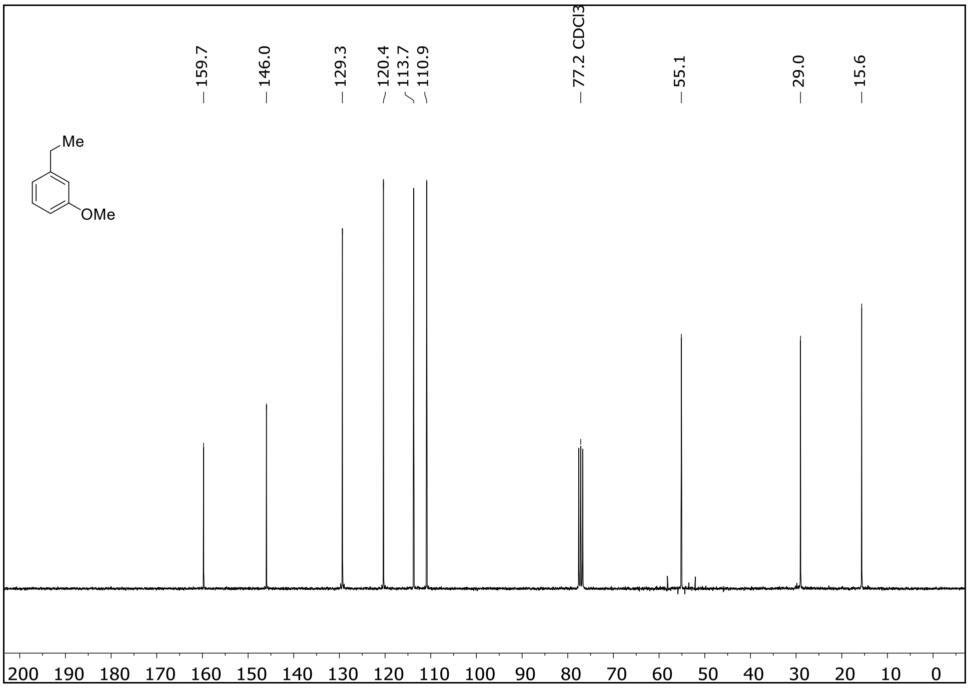

$^{13}C\{^1H\}$ NMR (75 MHz, $CDCl_3$)

1,2-Diphenylpropan-1-one (7a)

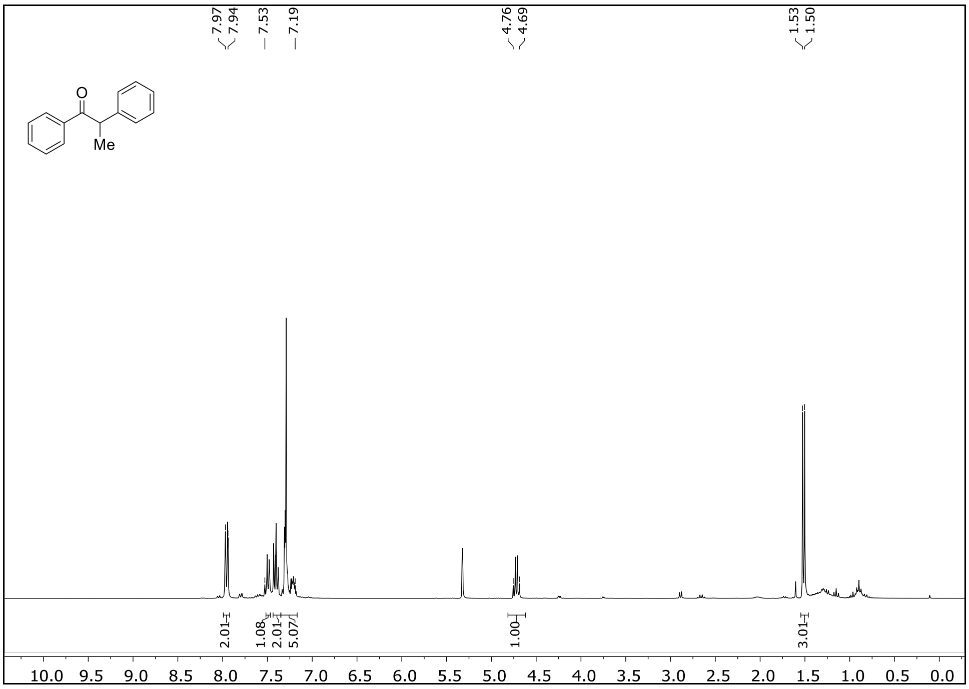

^1H NMR (300 MHz, CD_2Cl_2)

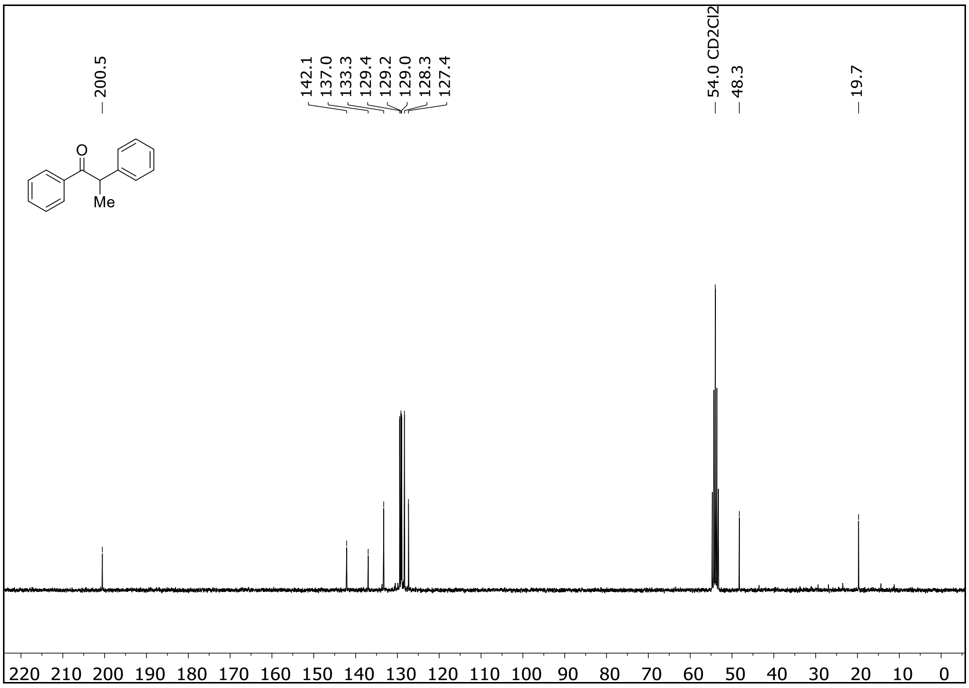

$^{13}C\{^{1}H\}$ NMR (75 MHz, CD_2Cl_2)

2-(4-Ethylphenyl)-1-phenylethan-1-one (7b)

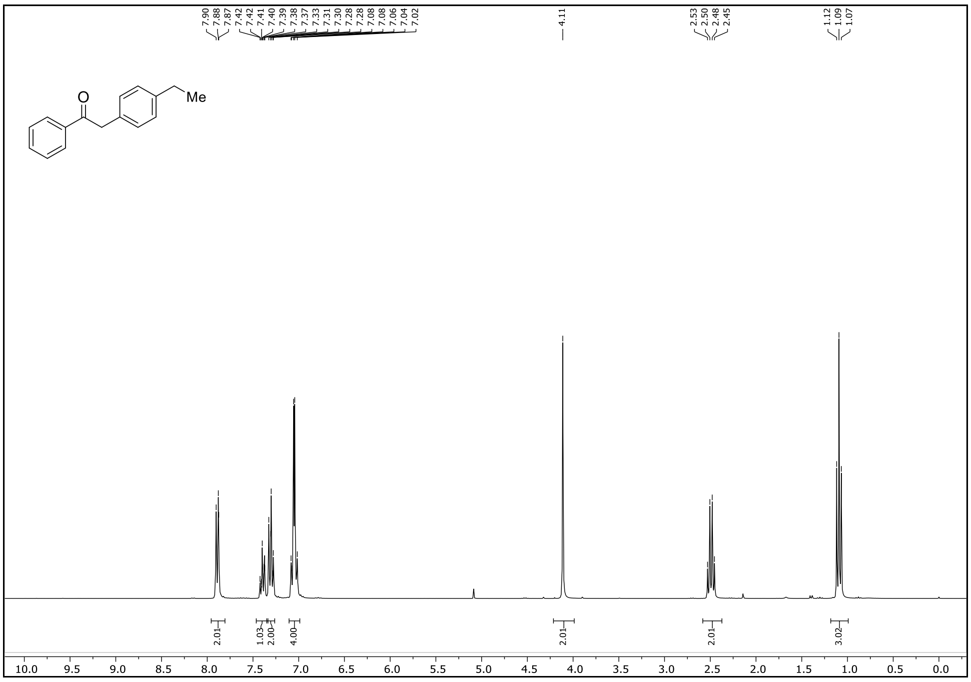

^{1}H NMR (300 MHz, $CDCl_3$)

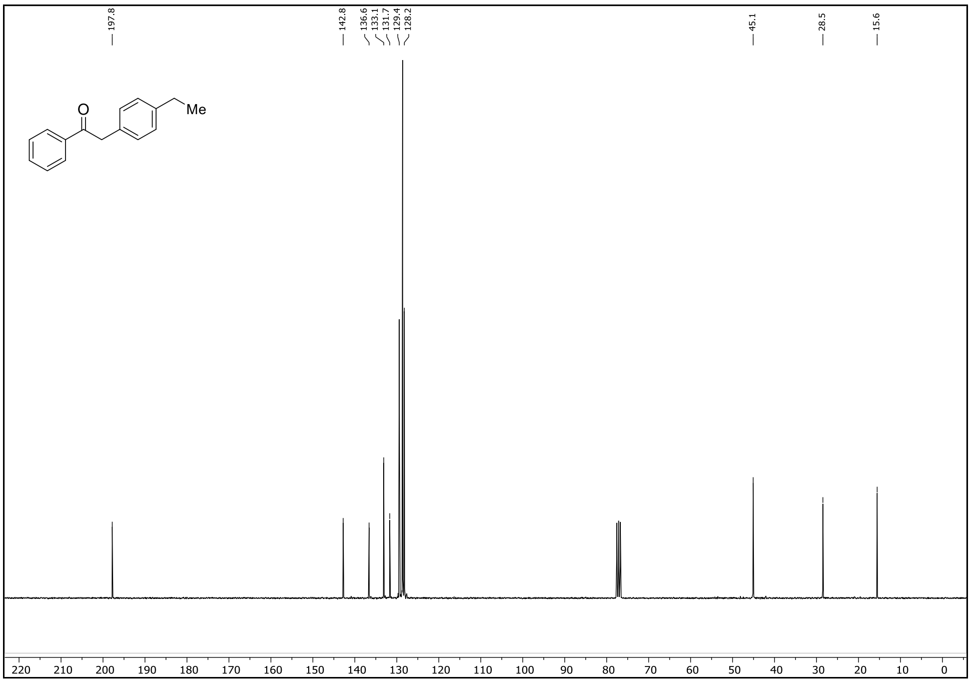

$^{13}C\{^{1}H\}$ NMR (75 MHz, $CDCl_3$)

Phenyl(1,2,3,4-tetrahydronaphthalen-1-yl)methanone (7d)

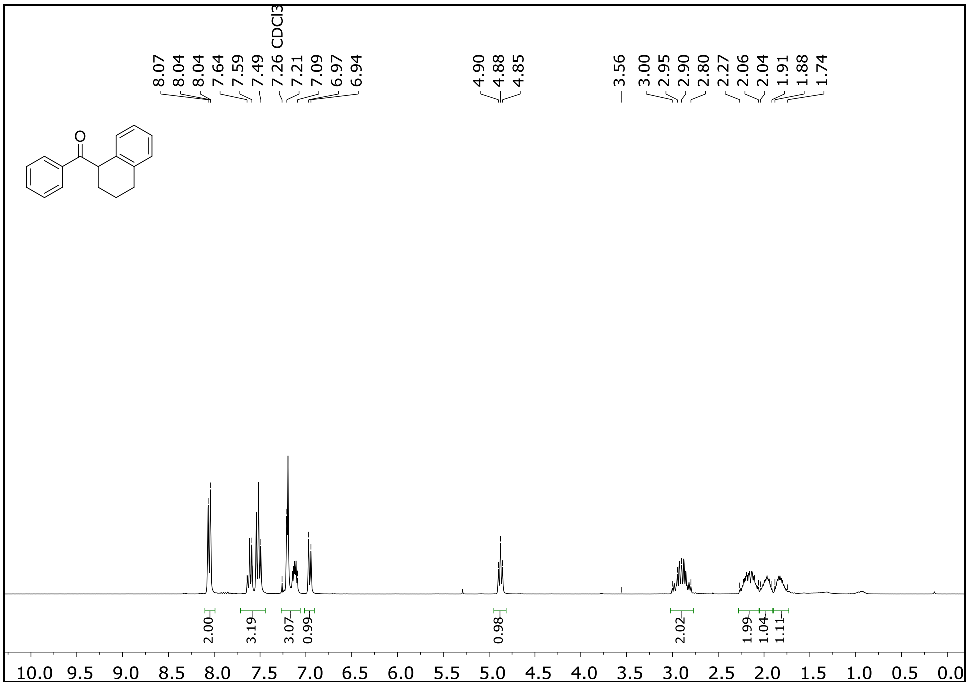

^{1}H NMR (300 MHz, $CDCl_3$)

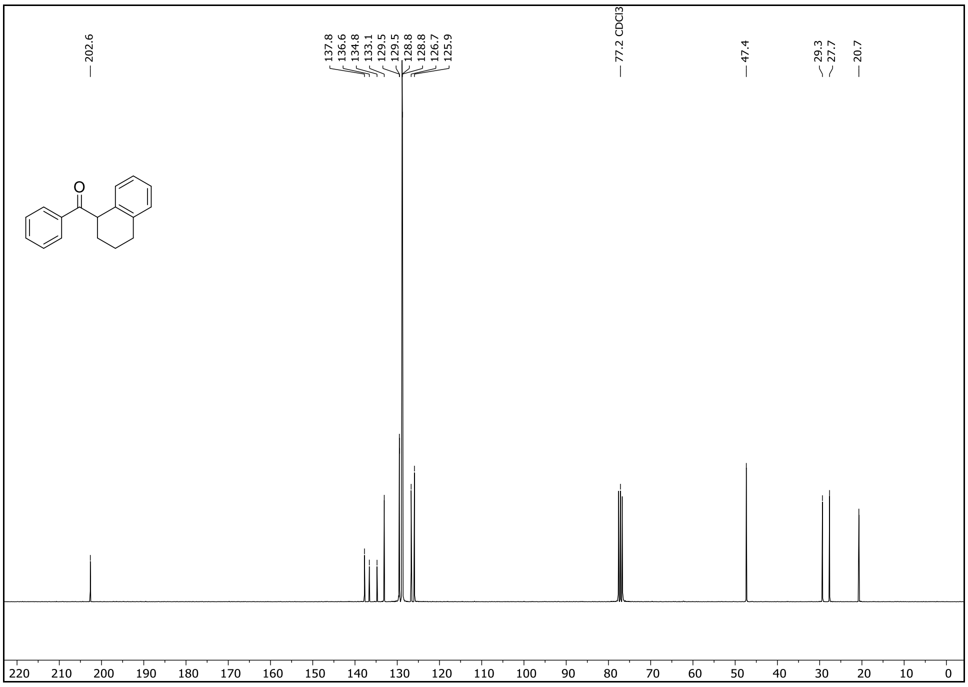

$^{13}C\{^{1}H\}$ NMR (75 MHz, $CDCl_3$)

2-Benzoyl-3-methoxy-5,6,7,8-tetrahydronaphthaline (7e)

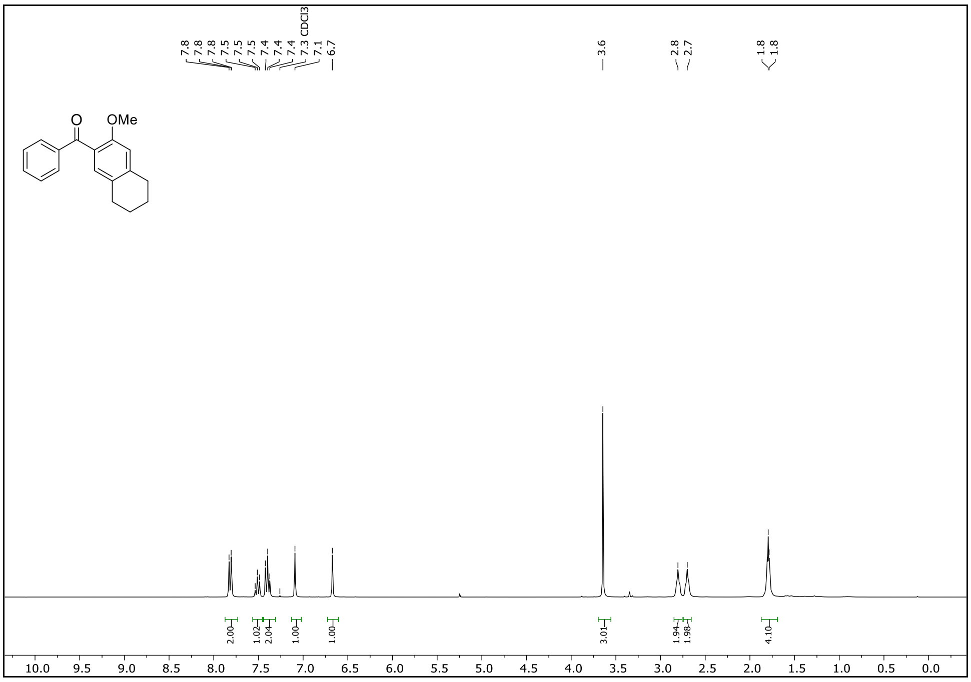

^{1}H NMR (300 MHz, $CDCl_3$)

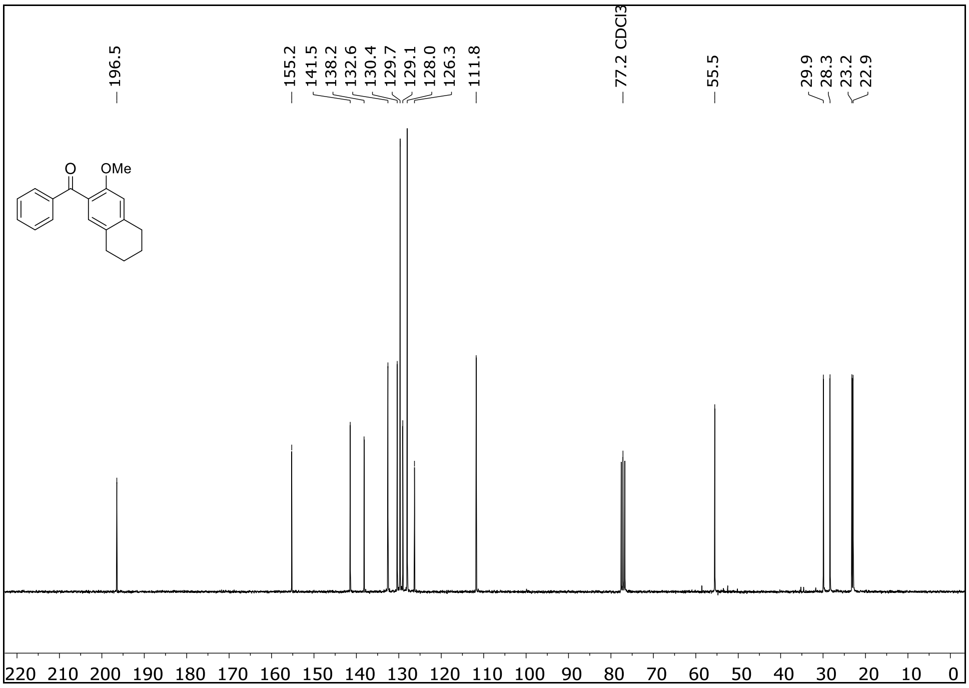

$^{13}C\{^{1}H\}$ NMR (75 MHz, $CDCl_3$)

1-(4-Methoxyphenyl)-2-phenylpropan-1-one (7f)

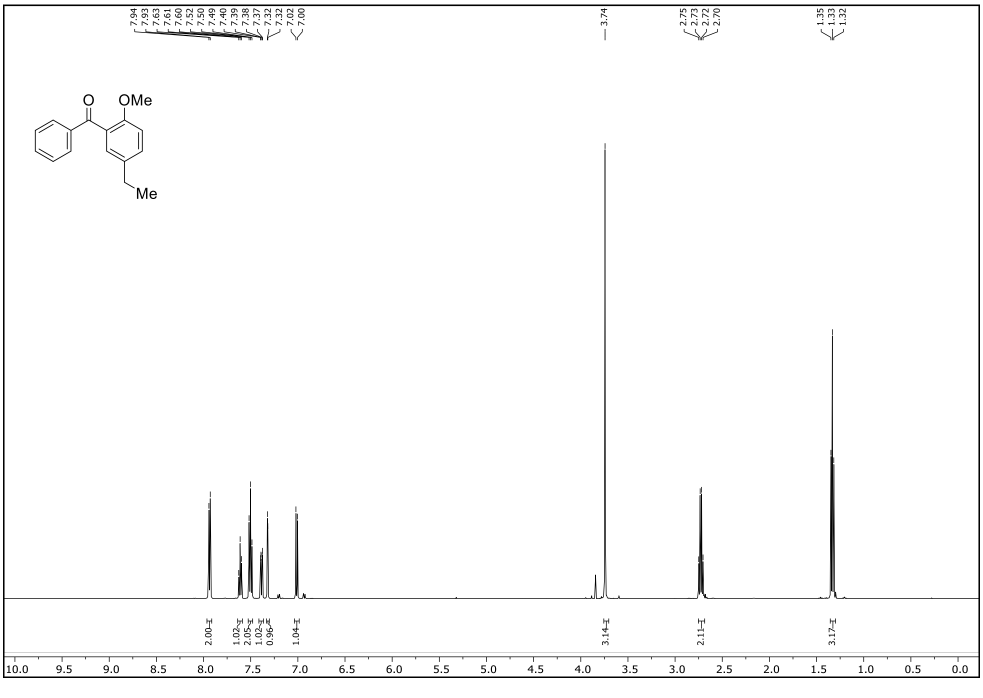

^{1}H NMR (300 MHz, $CDCl_3$)

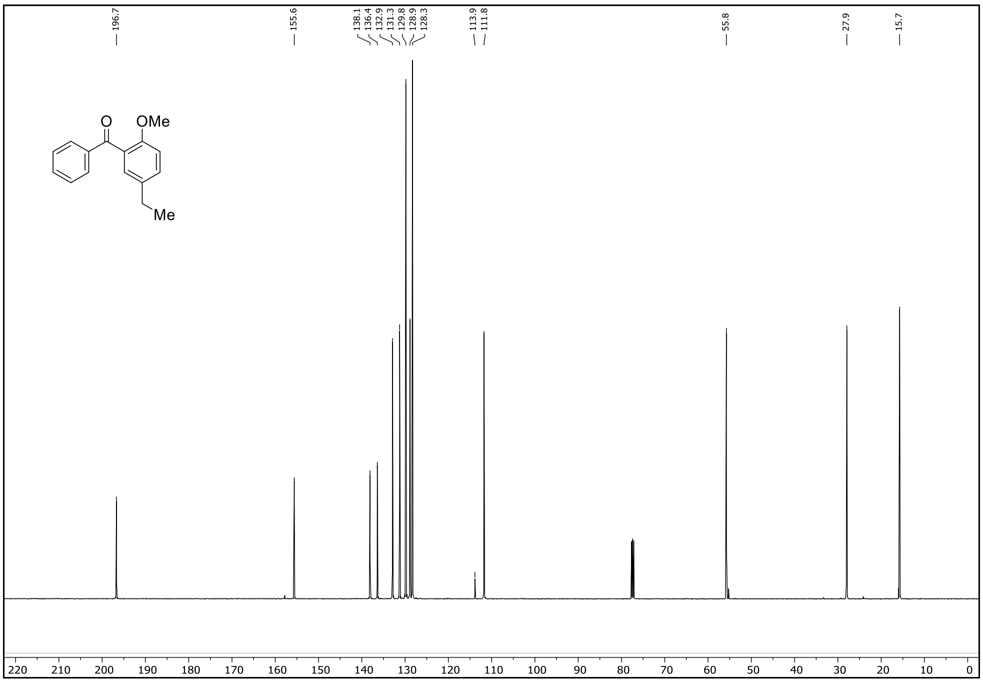

$^{13}C\{^1H\}$ NMR (75 MHz, $CDCl_3$)

***N*-(4-Ethylphenyl)benzamide (7i)**

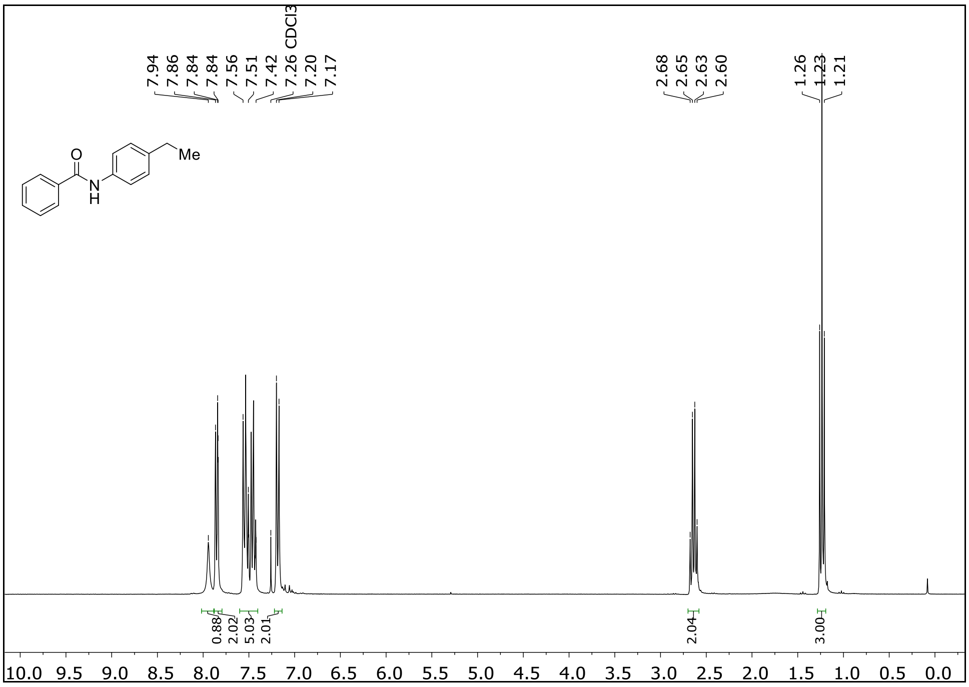

^1H NMR (300 MHz, $CDCl_3$)

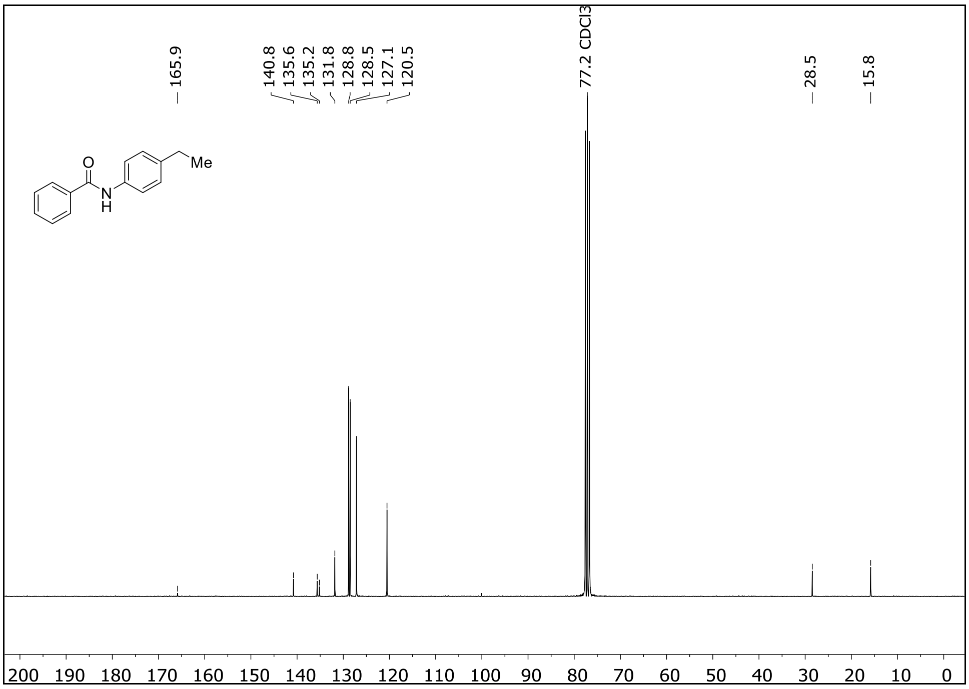

$^{13}C\{^{1}H\}$ **NMR** (75 MHz, $CDCl_3$)

***N*-(4-Ethylphenyl)-*N*-methylbenzamide (7j)**

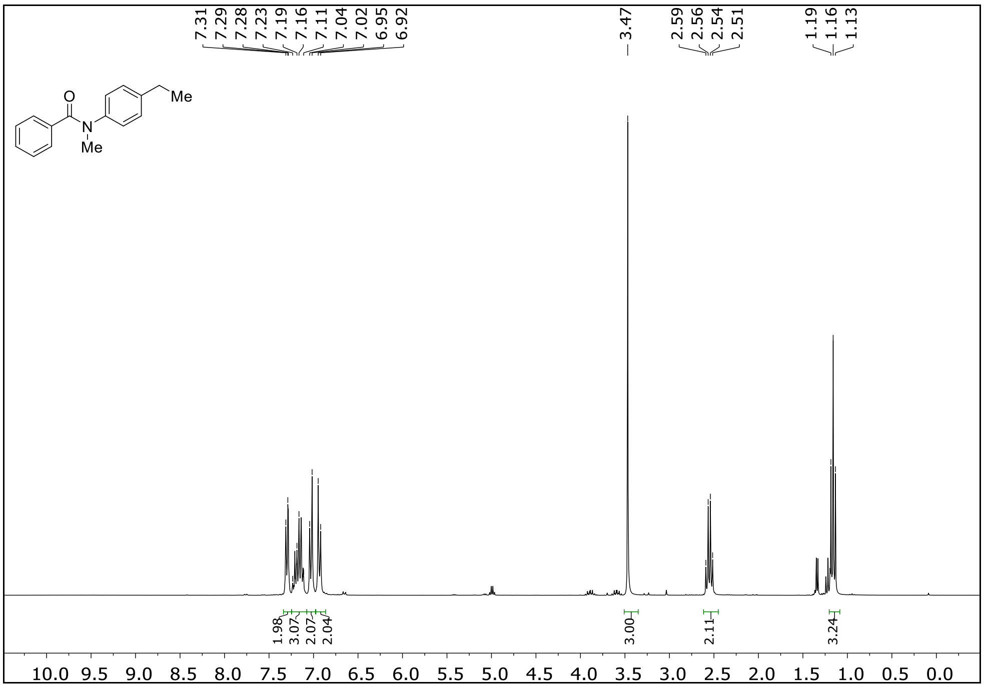

^{1}H **NMR** (300 MHz, $CDCl_3$)

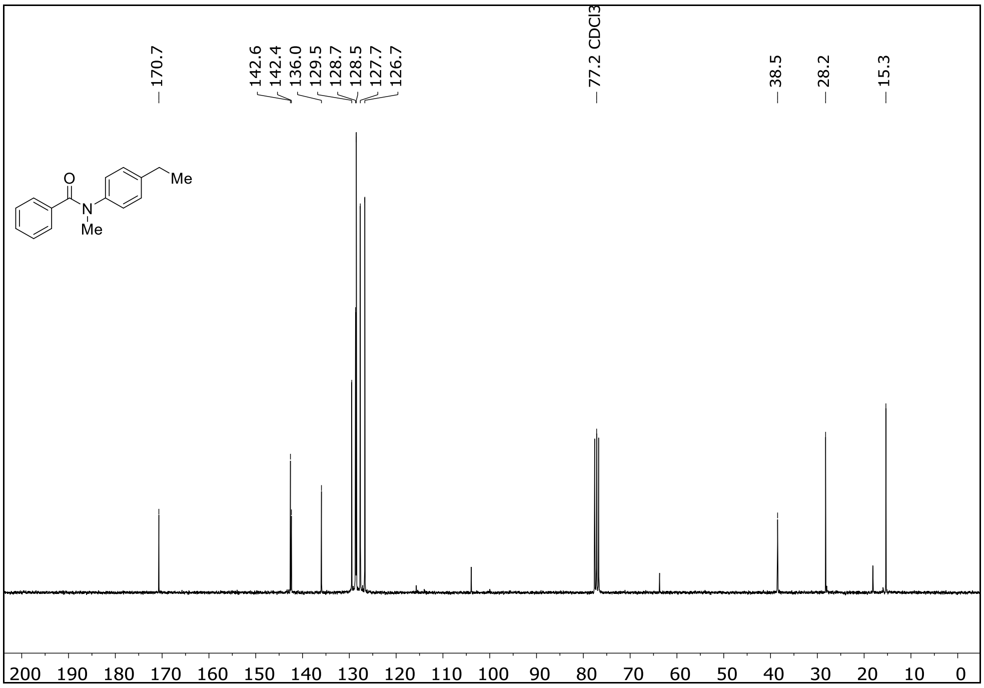

$^{13}C\{^1H\}$ NMR (75 MHz, $CDCl_3$)

(2-(Dimethylamino)-5-ethylphenyl)(phenyl)methanon (7k)

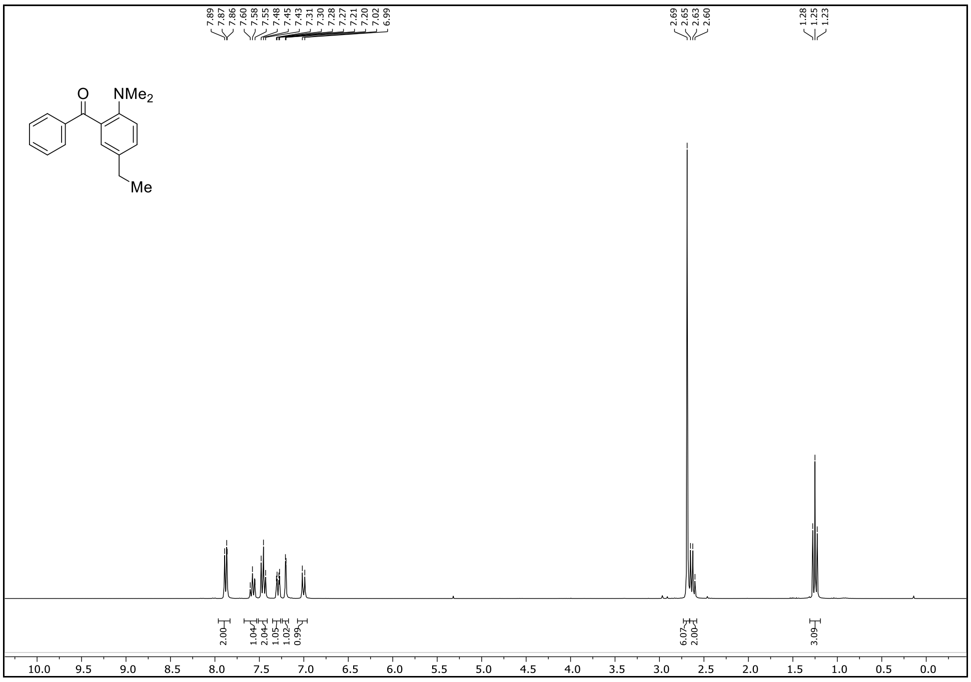

^1H NMR (300 MHz, $CDCl_3$)

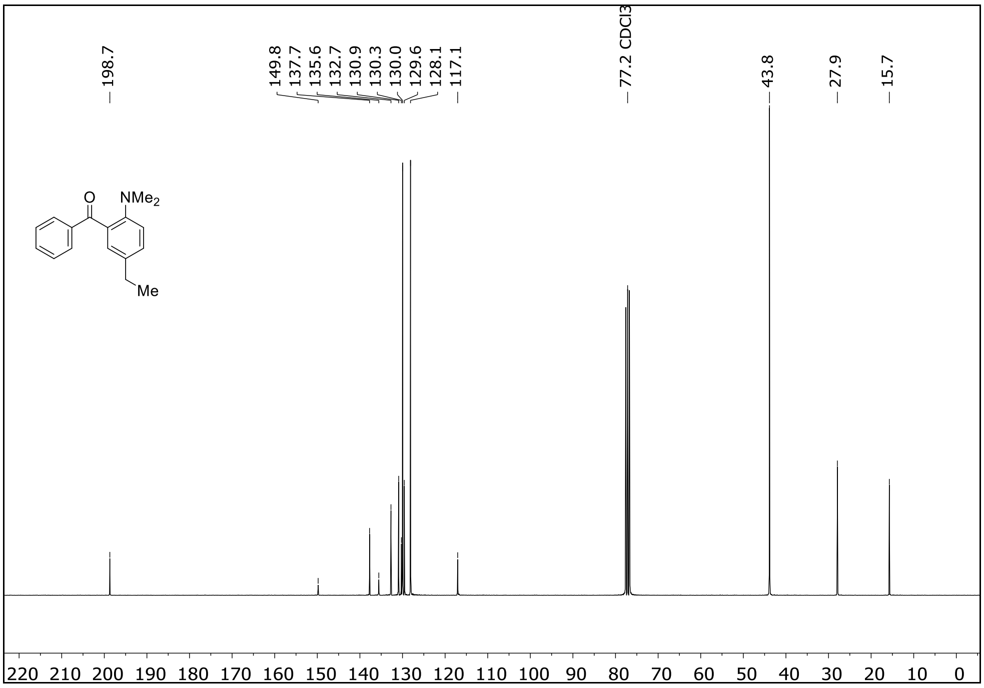

$^{13}C\{^{1}H\}$ NMR (75 MHz, $CDCl_3$)

2,5-Dimethoxybenzophenone (7l)

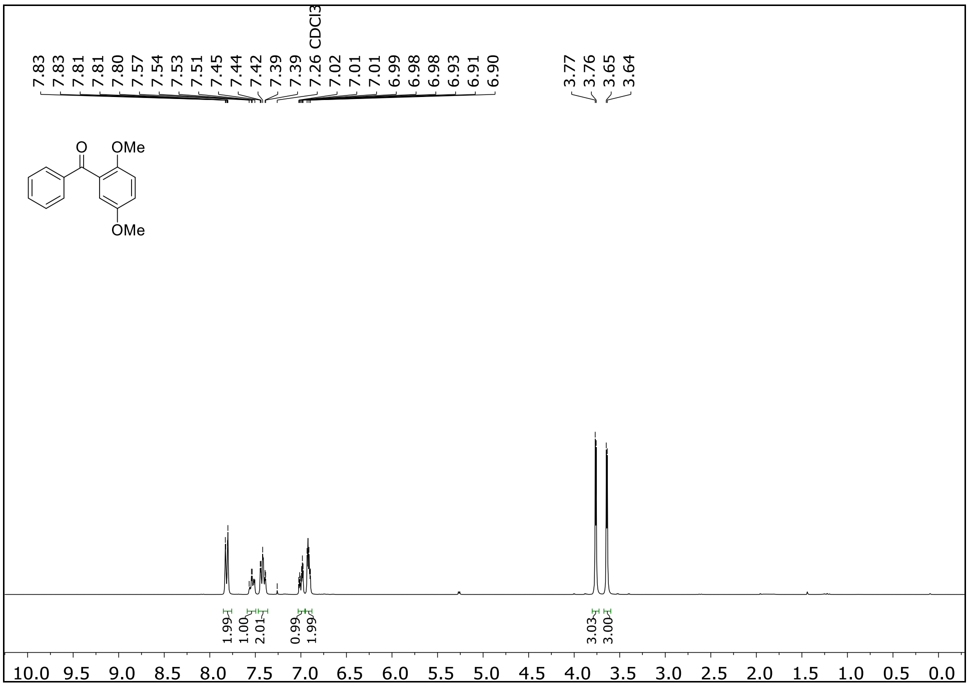

^{1}H NMR (300 MHz, $CDCl_3$)

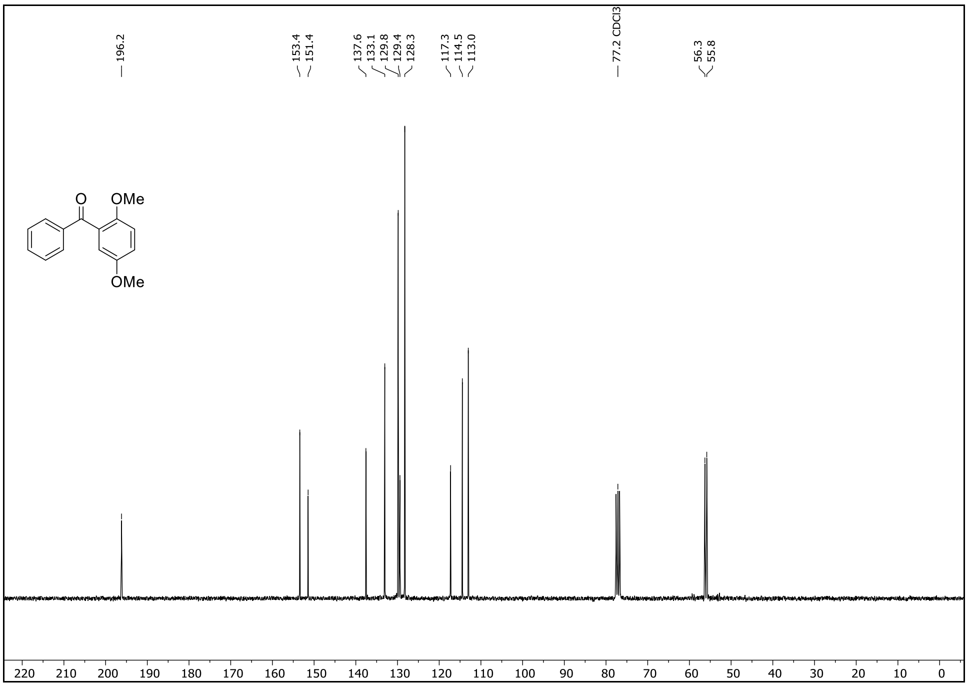

$^{13}C\{^1H\}$ NMR (75 MHz, $CDCl_3$)

(3-Ethyl-2-methoxyphenyl)(phenyl)methanone (7m)

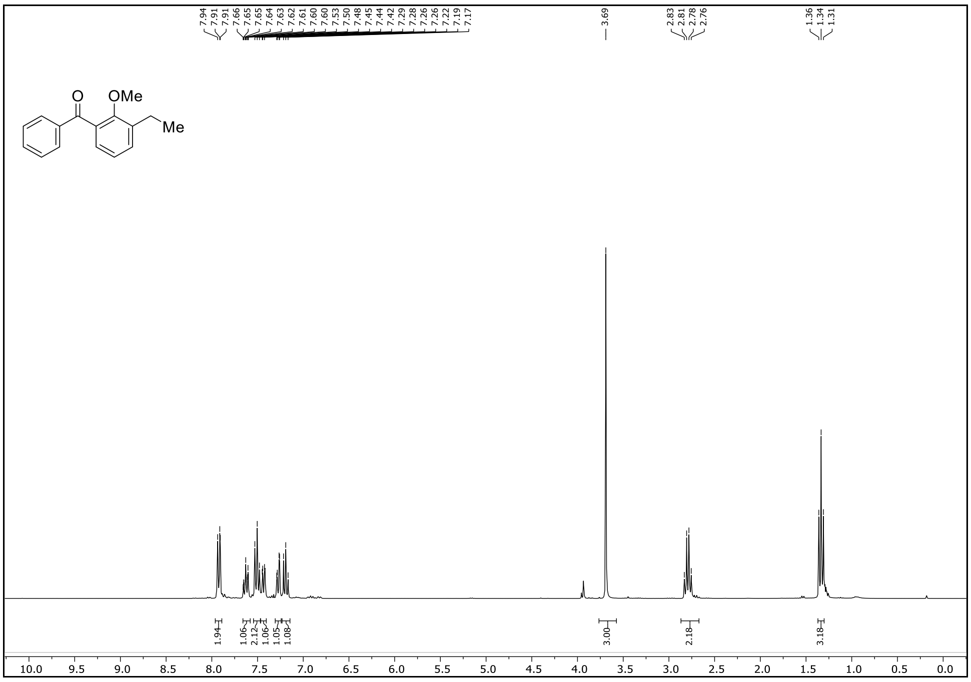

^1H NMR (300 MHz, $CDCl_3$)

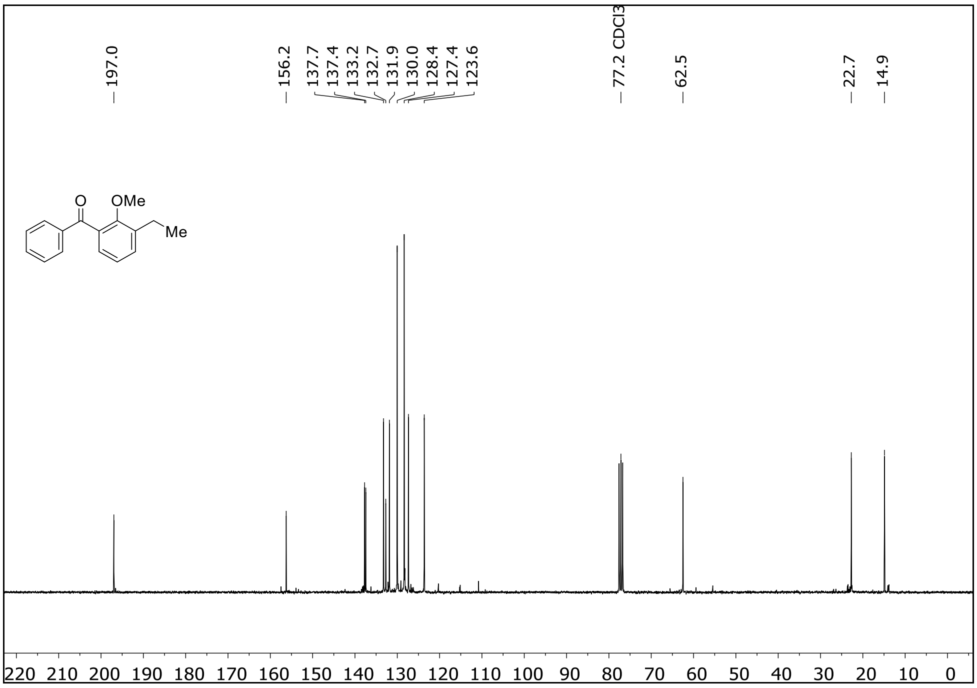

$^{13}C\{^1H\}$ NMR (75 MHz, $CDCl_3$)

(4-Ethyl-2-methoxyphenyl)(phenyl)methanone (7n)

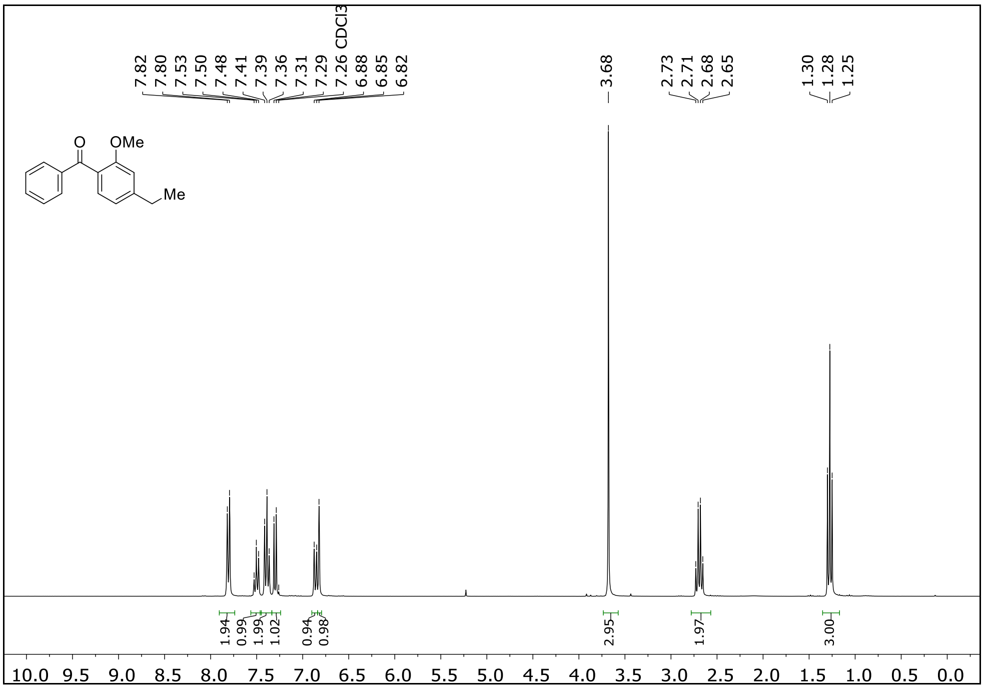

^1H NMR (300 MHz, $CDCl_3$)

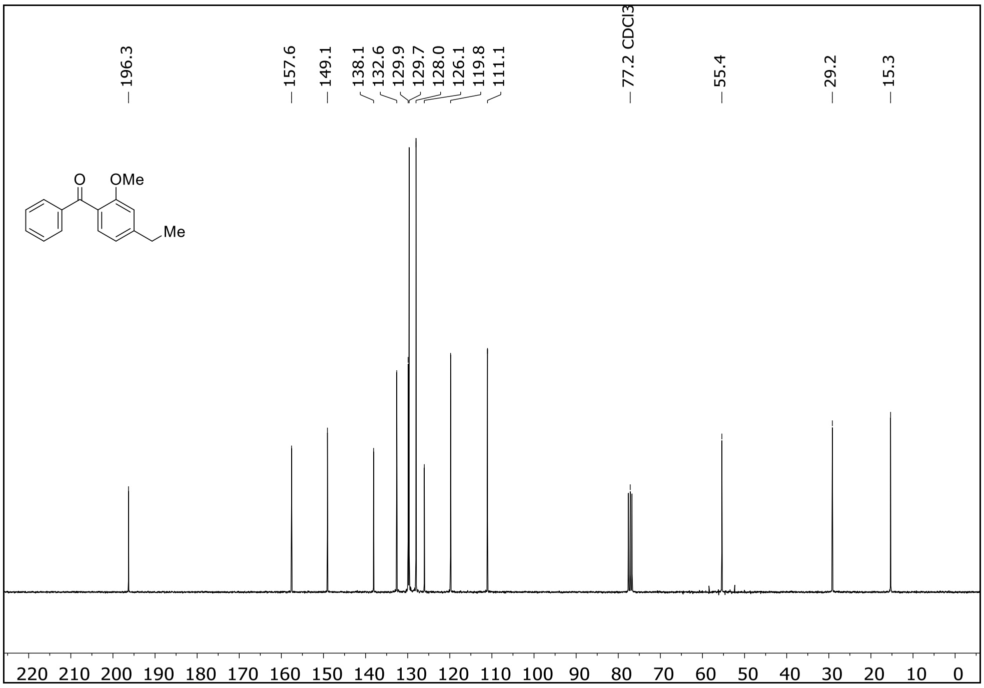

$^{13}C\{^1H\}$ NMR (75 MHz, $CDCl_3$)

3-Isopropyl-2-methoxybenzophenone (7o)

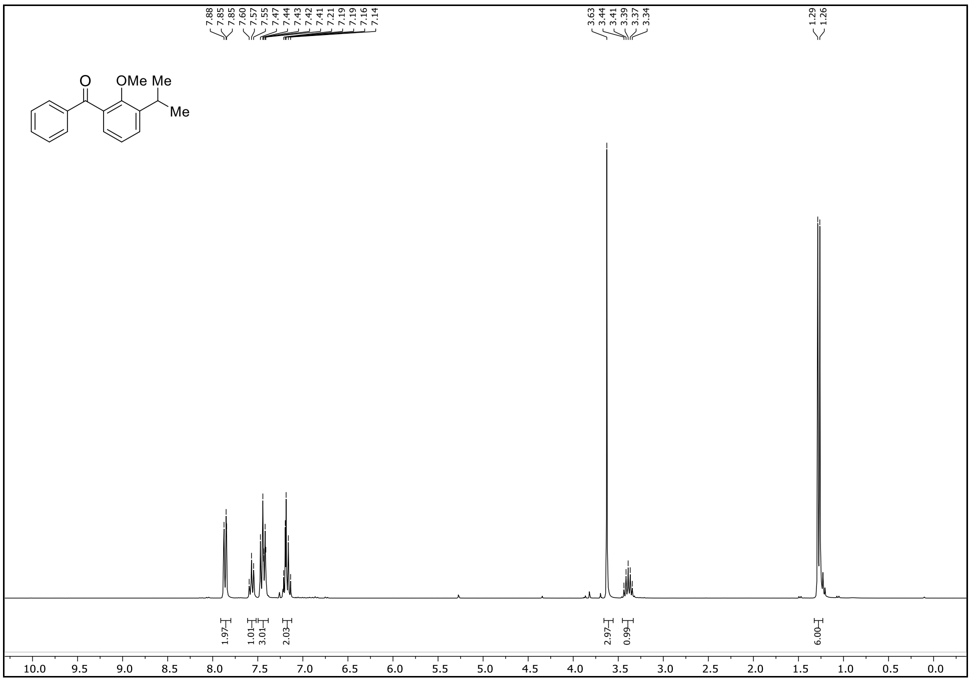

^1H NMR (300 MHz, $CDCl_3$)

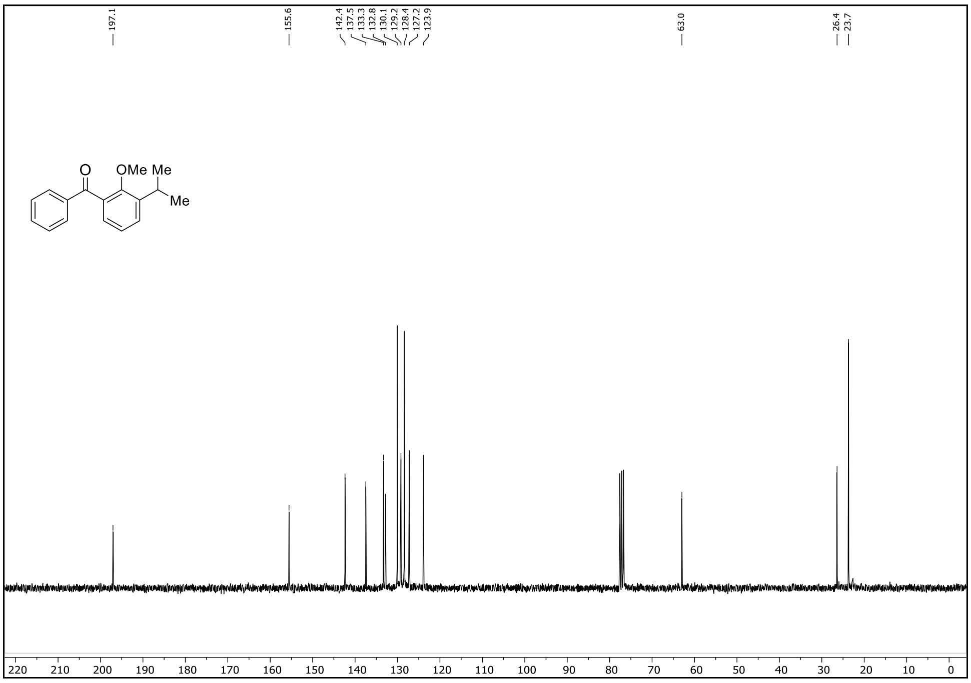

$^{13}C\{^{1}H\}$ NMR (75 MHz, $CDCl_3$)

3-Isopropyl-2-methoxybenzophenone (7p)

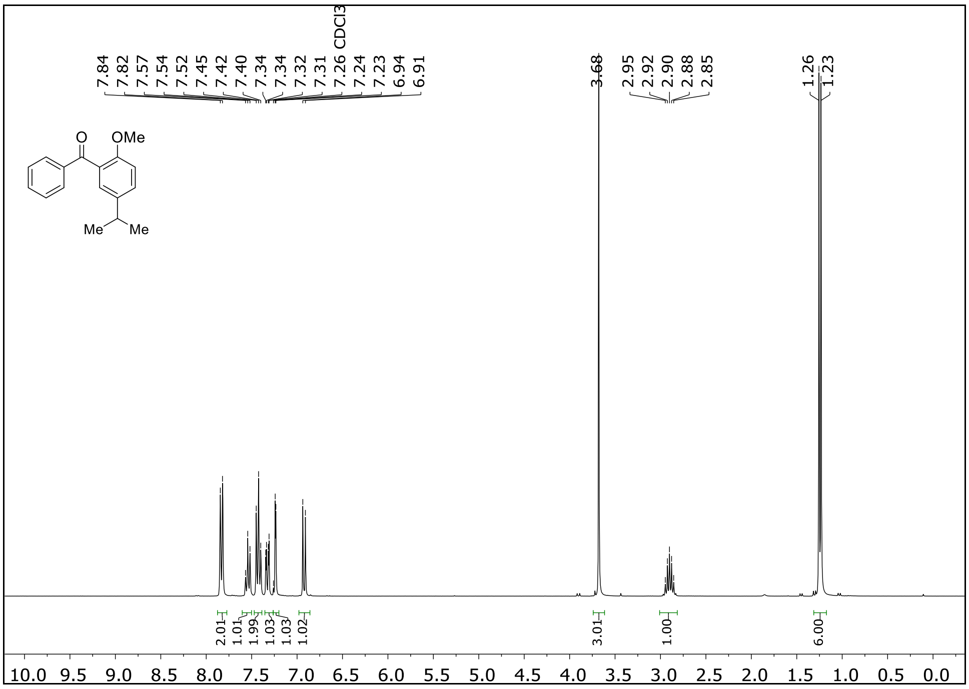

^{1}H NMR (300 MHz, $CDCl_3$)

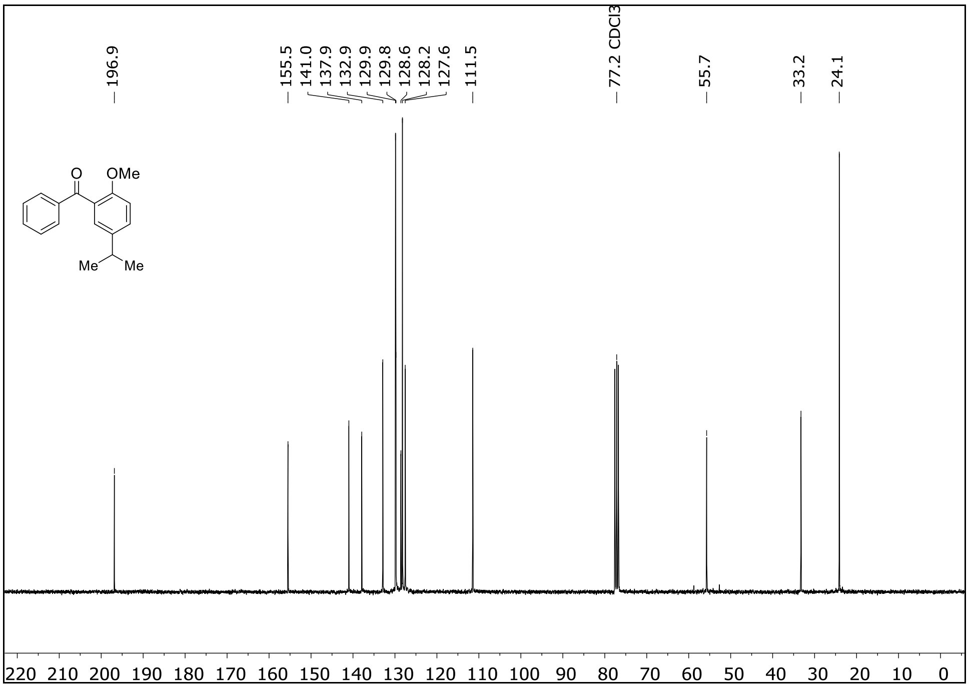

$^{13}C\{^1H\}$ NMR (75 MHz, $CDCl_3$)

3-(4-Methoxyphenyl)-1-phenylprop-2-yn-1-one (7q)

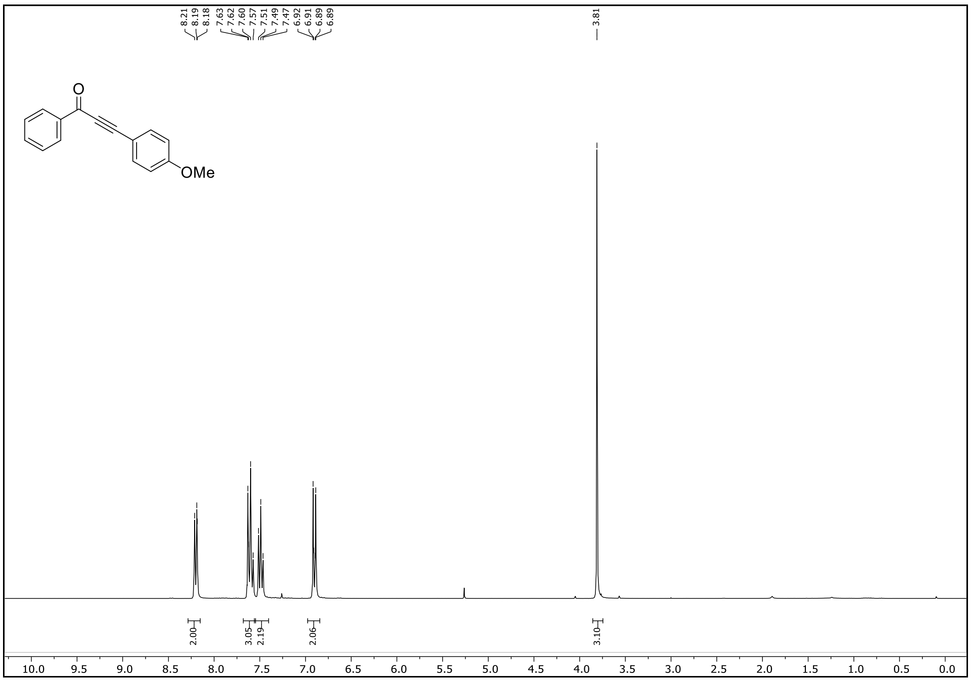

^1H NMR (300 MHz, $CDCl_3$)

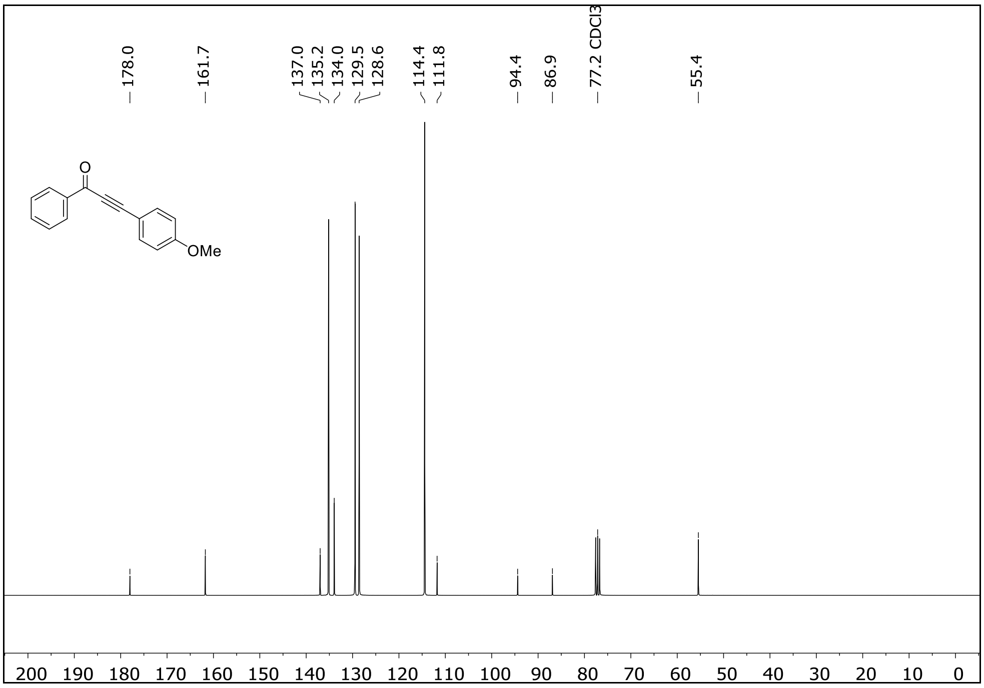

$^{13}C\{^1H\}$ NMR (75 MHz, $CDCl_3$)

3-Ethyl-1-phenylheptan-1-one (9a)

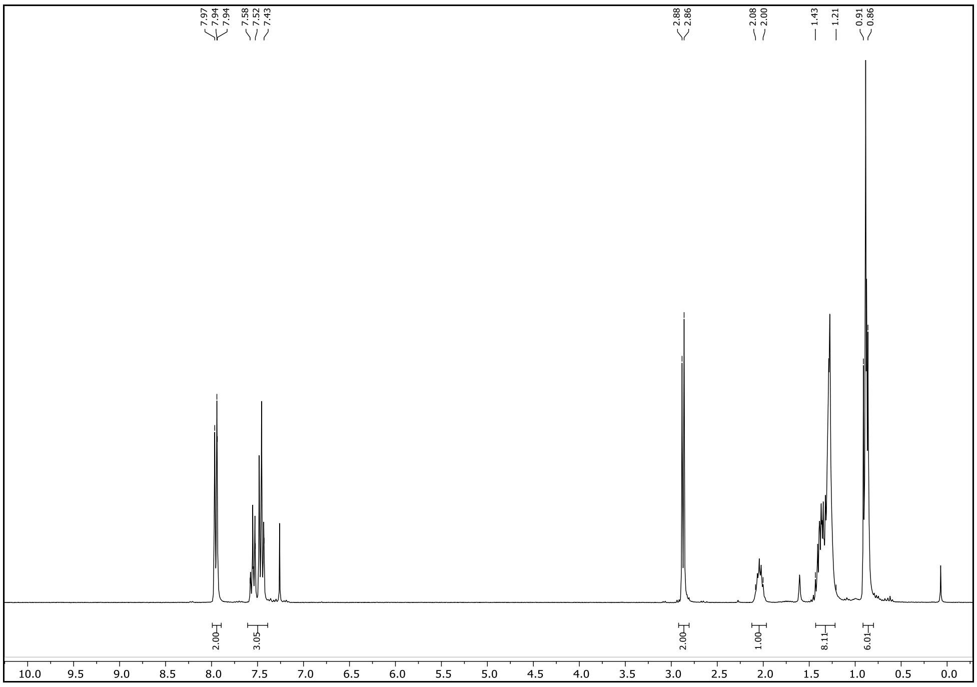

^1H NMR (300 MHz, $CDCl_3$)

3-Ethyl-1-naphthalen-2-ylheptan-1-one (9b)

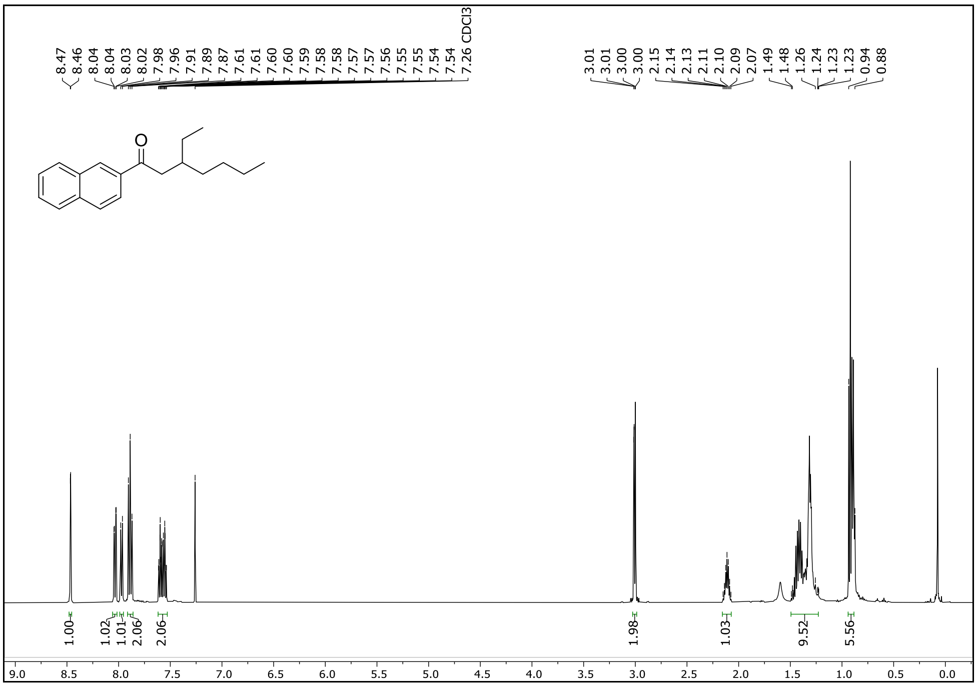

^{1}H NMR (500 MHz, $CDCl_3$)

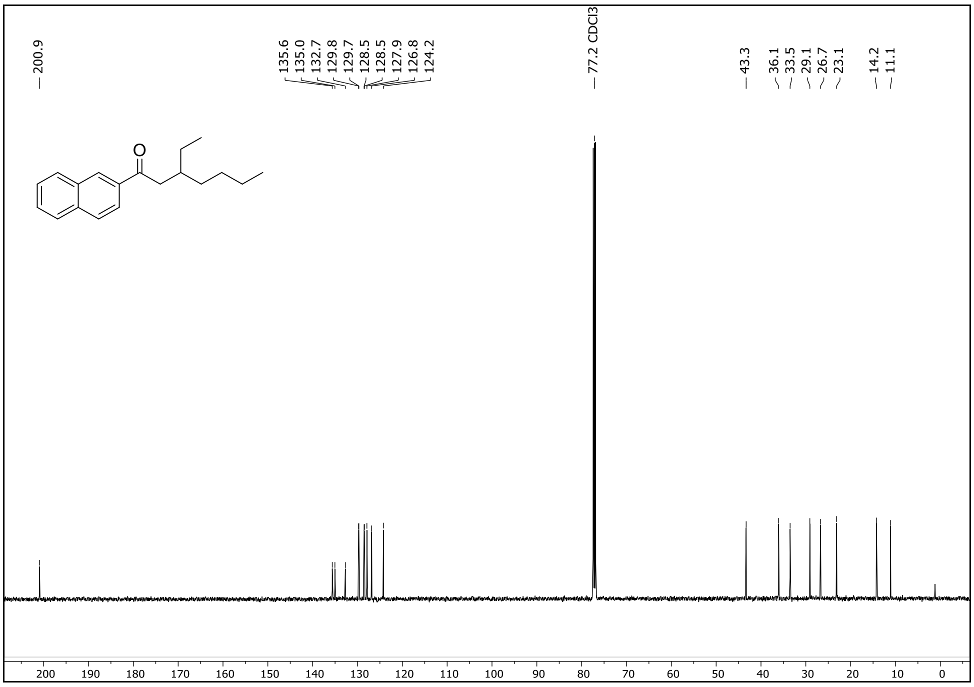

^{13}C{^{1}H} NMR (125 MHz, $CDCl_3$)

3-Ethyl-1-(3-methoxyphenyl)heptan-1-one (9c)

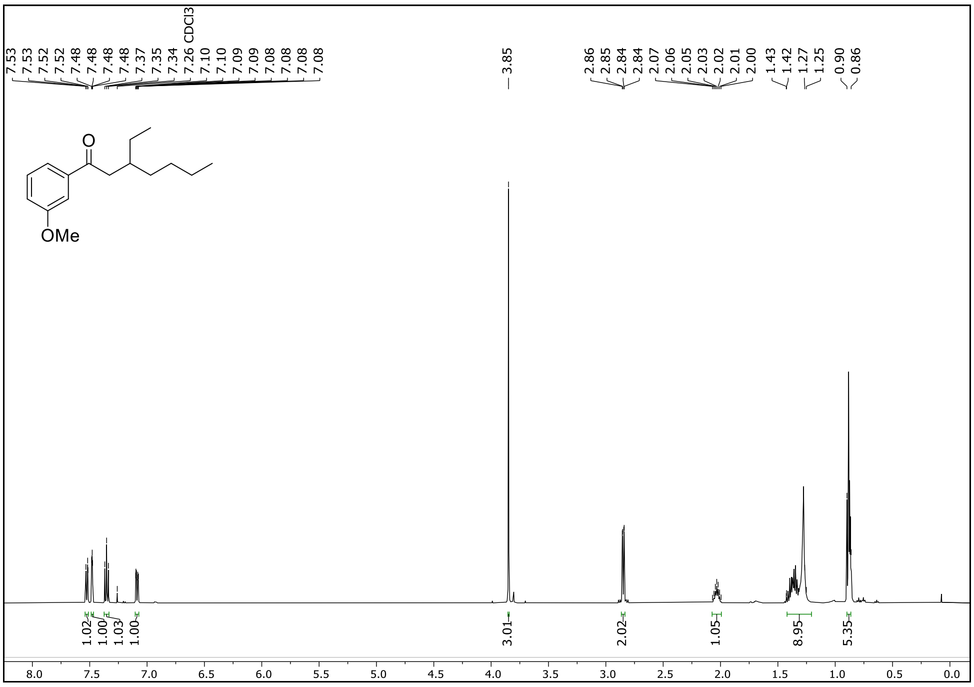

^{1}H NMR (500 MHz, $CDCl_3$)

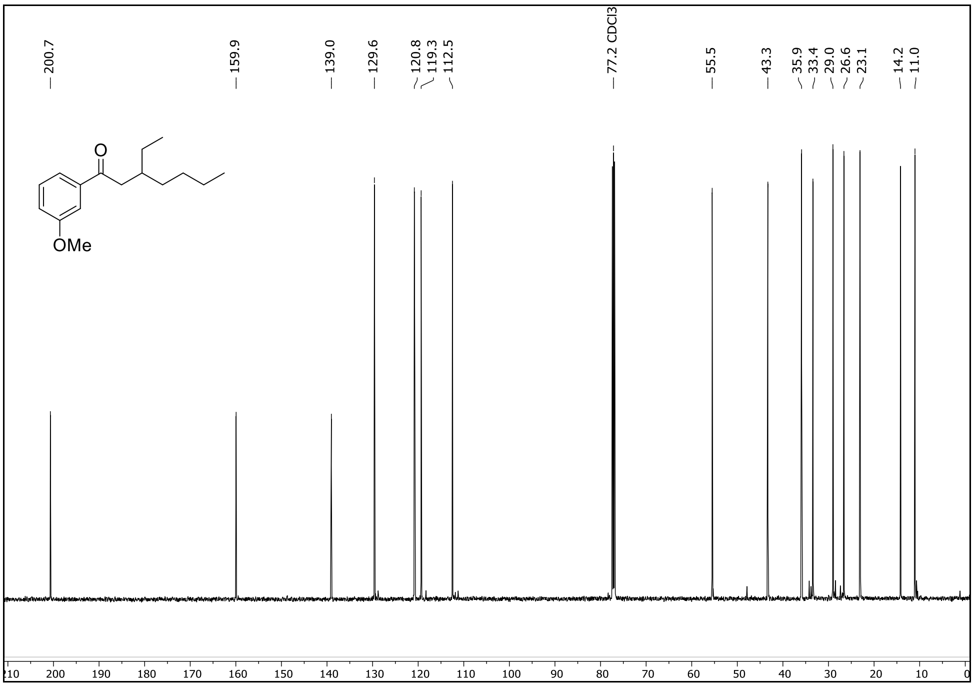

$^{13}C\{^1H\}$ NMR (125 MHz, $CDCl_3$)

3-Ethyl-1-(3-methylphenyl)heptan-1-one (9d)

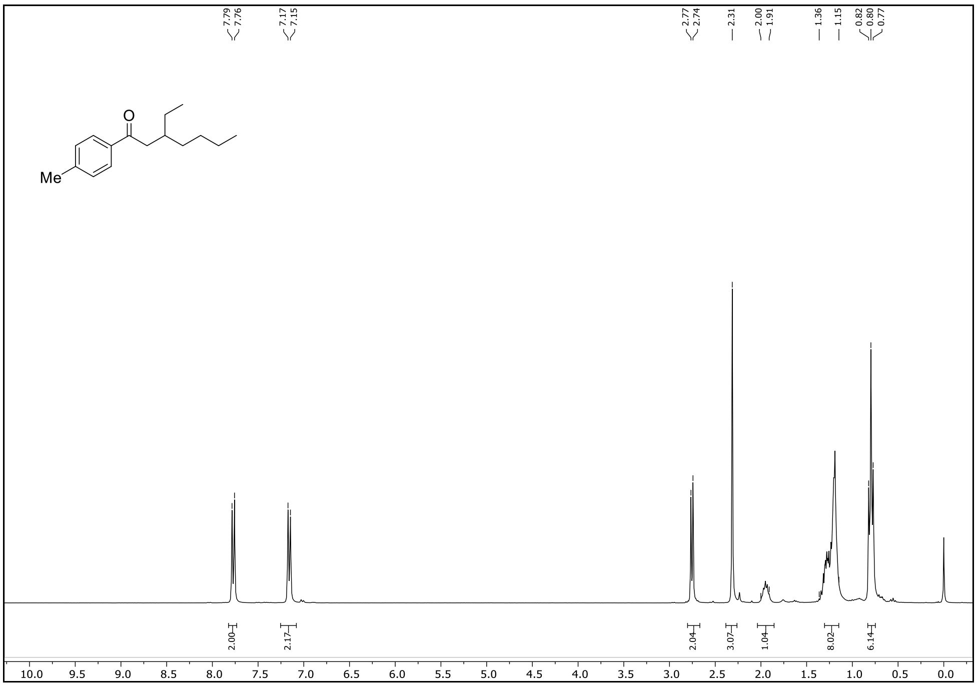

^{1}H NMR (300 MHz, $CDCl_3$)

3-Ethyl-1-(4-propan-2-ylphenyl)heptan-1-one (9e)

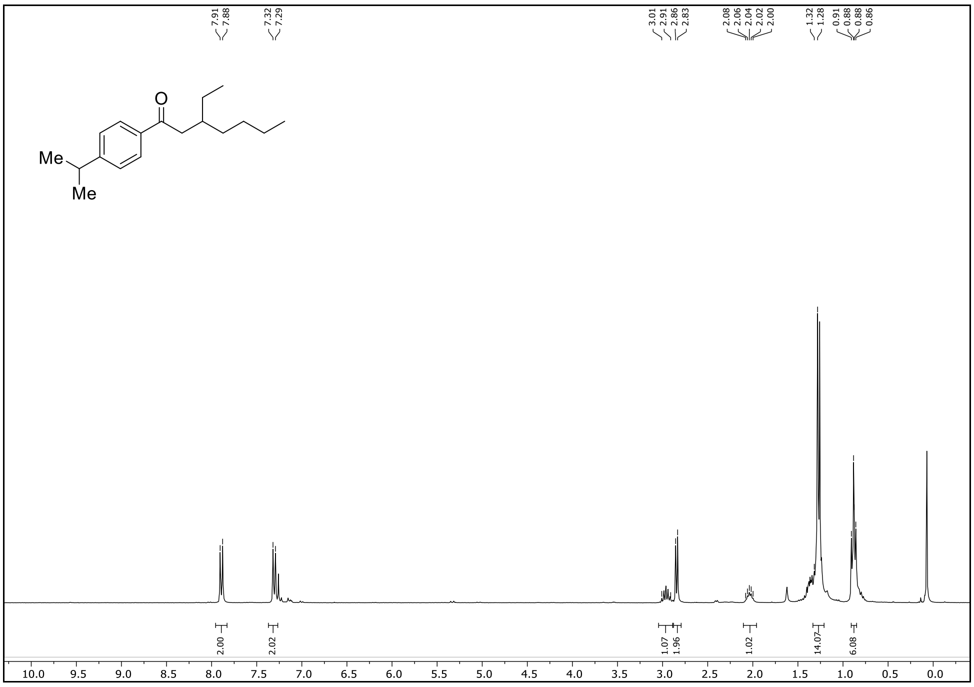

^{1}H NMR (300 MHz, $CDCl_3$)

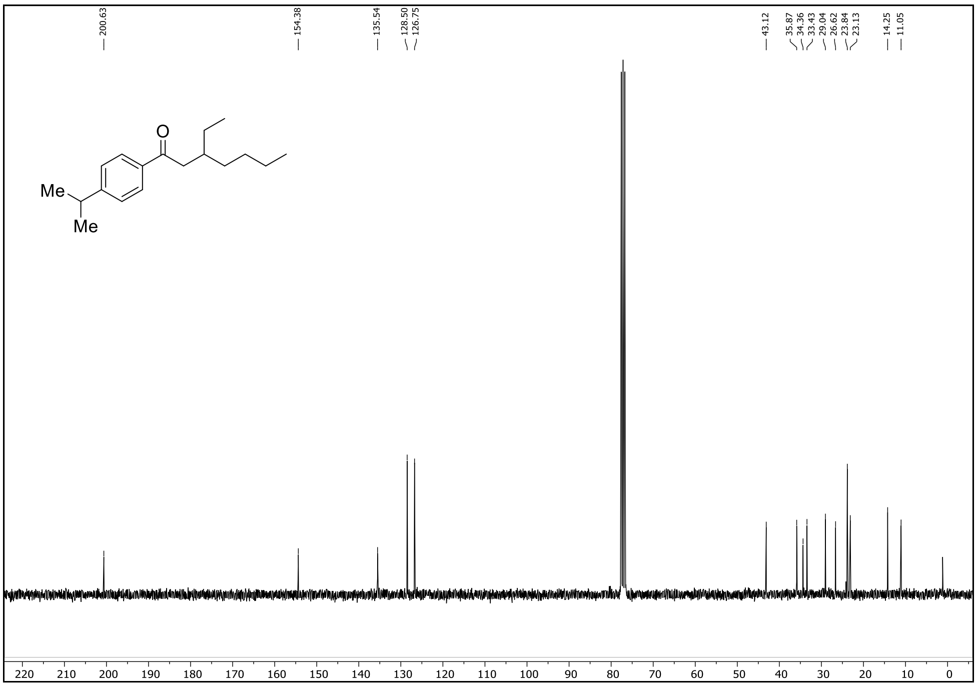

$^{13}C\{^{1}H\}$ NMR (75 MHz, $CDCl_3$)

3-Ethyl-1-[4-(trifluoromethyl)phenyl]heptan-1-one (9f)

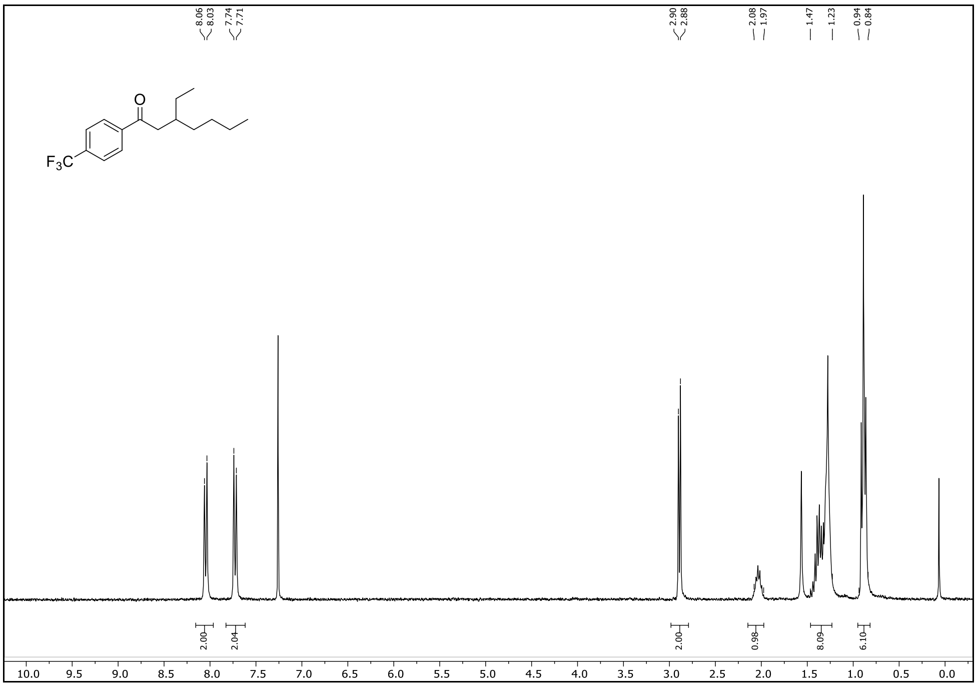

^{1}H NMR (300 MHz, $CDCl_3$)

1-Cyclohexyl-3-ethylheptan-1-one (9g)

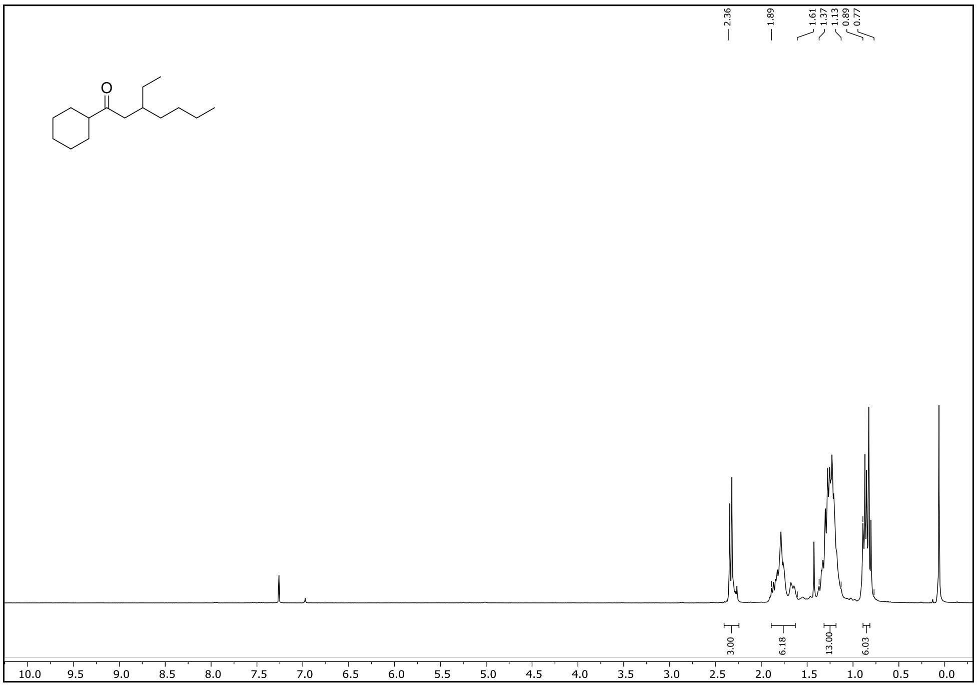

^{1}H NMR (300 MHz, $CDCl_3$)

3-Ethyl-1-pyridin-2-ylheptan-1-one (9h)

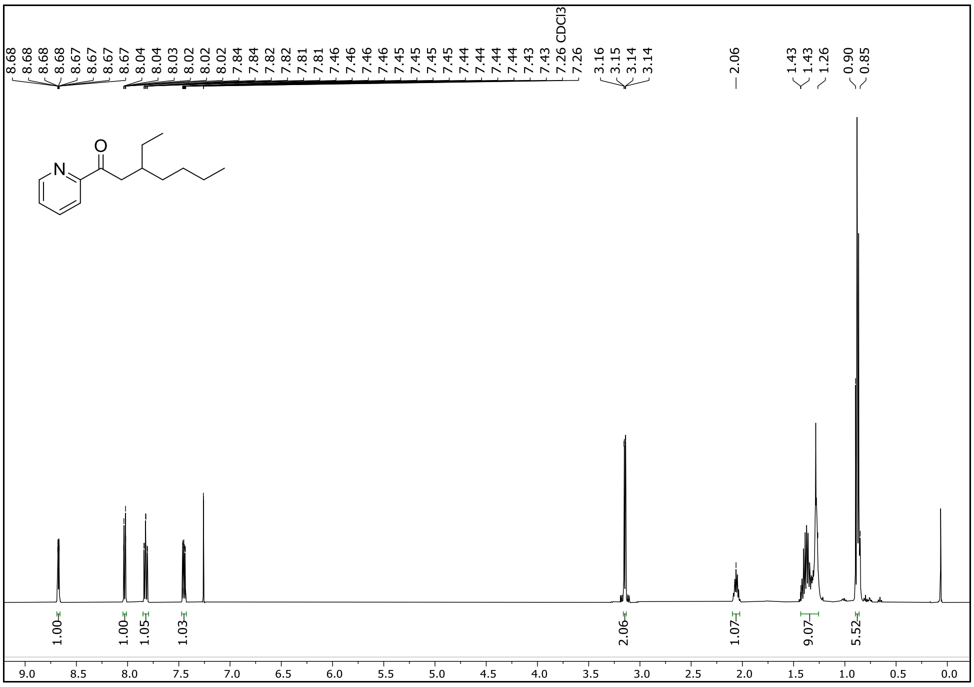

^{1}H NMR (500 MHz, $CDCl_3$)

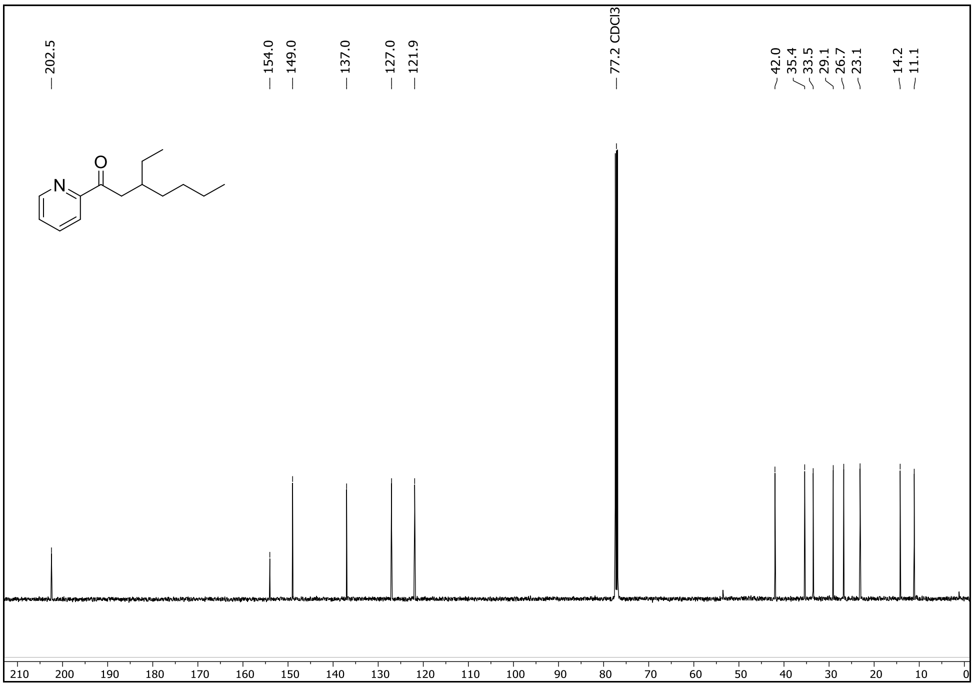

$^{13}C\{^{1}H\}$ NMR (125 MHz, $CDCl_3$)

1-Phenylpentan-1-one (10)

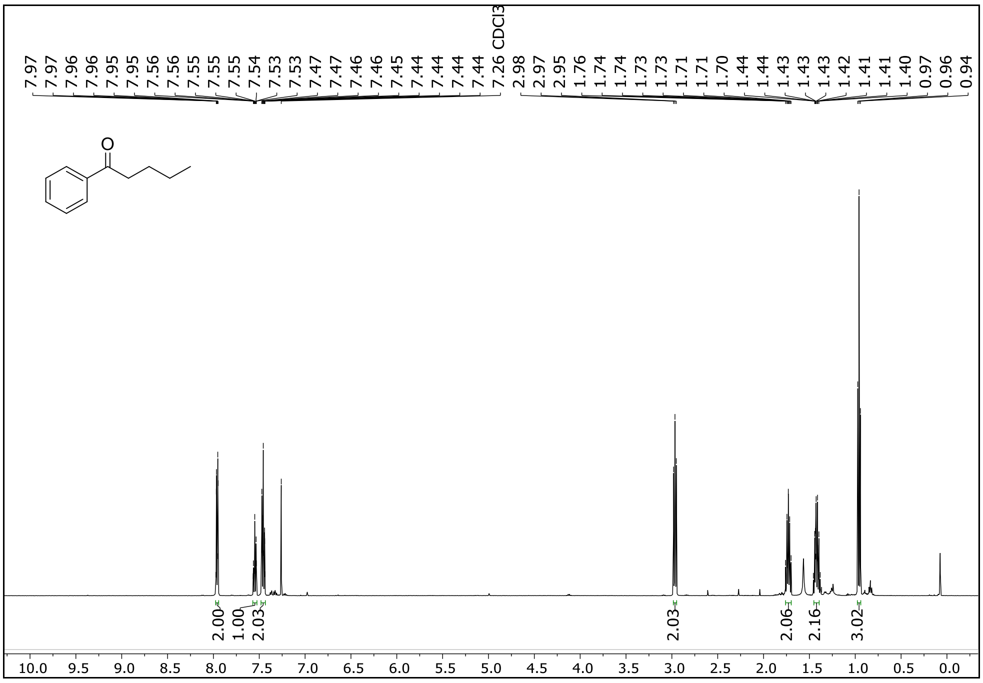

^{1}H NMR (500 MHz, $CDCl_3$)

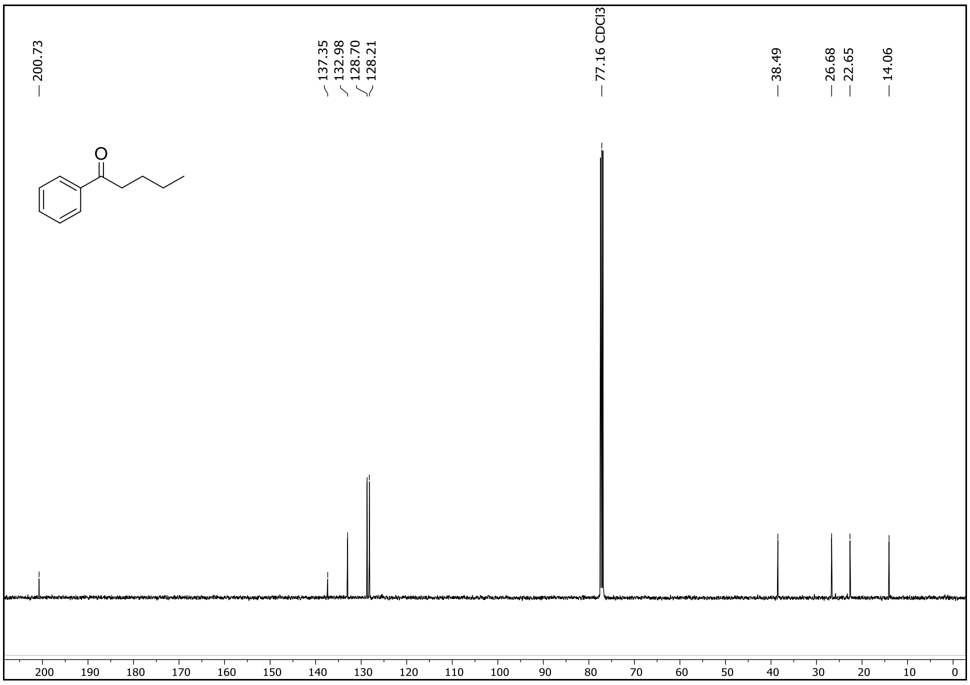

$^{13}C\{^1H\}$ NMR (125 MHz, $CDCl_3$)

8. References

[1] J. H. Harenberg, N. Weidmann, A. J. Wiegand, C. A. Hoefer, R. R. Annapureddy, P. Knochel, *Angew. Chem. Int. Ed.* **2021**, *60*, 14296.

[2] W. J. Kerr, A. J. Morrison, M. Pazicky, T. Weber, *Org. Lett.* **2012**, *14*, 2250.

[3] N. Radhoff, C. G. Daniliuc, A. Studer, *Angew. Chem. Int. Ed.* **2023**, *62*, e202304771.

[4] A. Bourboula, O. G. Mountanea, G. Krasakis, C. Mantzourani, M. G. Kokotou, C. G. Kokotos, G. Kokotos, *Eur. J. Org. Chem.* **2023**, *26,* e202300008.

[5] M. Colella, A. Tota, Y. Takahashi, R. Higuma, S. Ishikawa, L. Degennaro, R. Luisi, A. Nagaki, *Angew. Chem. Int. Ed.* **2020**, *59*, 10924.

[6] D. M. Rudzinski, C. B. Kelly, N. E. Leadbeater, *Chem. Comm.* **2012**, *48*, 9610.

[7] R. N. Gaykar, A. Guin, S. Bhattacharjee, A. T. Biju, *Org. Lett.* **2019**, *21*, 9613.

[8] B. Liu, Q. Liu, *Chin. J. Chem.* **2023**, *41*, 3528.

[9] J.-F. Yang, Y.-F. Liu, L.-L. Wei, Y.-Q. Zhao, L. Shi, *Tetrahedron Lett.* **2023**, *124*, 154585.

[10] C. Schneider, R. Jackstell, B. U. W. Maes, M. Beller, *Eur. J. Org. Chem.* **2020**, 932.

[11] R. C. Betori, C. M. May, K. A. Scheidt, *Angew. Chem. Int. Ed.* **2019**, *58*, 16490.

[12] W. Kong, C. Yu, H. An, Q. Song, *Org. Lett.* **2018**, *20*, 349.

[13] X. Zhu, C. Ye, Y. Li, H. Bao, *Chem. Eur. J.* **2017**, *23*, 10254.

[14] F. Su, J. Zou, X. Lv, F. Lu, Y. Long, K. Tang, B. Li, H. Chai, X. Wu, Y. R. Chi, *Angew. Chem. Int. Ed.* **2023**, *62*, e202303388.

[15] C.-C. Bao, Y.-L. Luo, H.-Z. Du, B.-T. Guan, *Sci. China Chem.* **2021**, *64*, 1349.

[16] L. Li, P. Cai, Q. Guo, S. Xue, *J. Org. Chem.* **2008**, *73*, 3516.

[17] L.-J. Chen, C.-J. Kuo, C.-F. Liang, *J. Org. Chem.* **2023**, *88*, 10501.

[18] Z. Zhang, Y.-H. Liu, X. Zhang, X.-C. Wang, *Tetrahedron* **2019**, *75*, 2763.

[19] G. Pandey, S. K. Tiwari, B. Singh, K. Vanka, S. Jain, *Chem. Comm.* **2017**, *53*, 12337.

[20] H. Tomioka, K. Kimoto, H. Murata, Y. Izawa, *J. Chem. Soc., Perkin Trans. 1* **1991**, 471.

[21] A. Mohanty, M. K. Nayak, S. Roy, *Org. Biomol. Chem.* **2023**, *21*, 5601.

[22] Y. Li, T. Yang, S. Wang, Z. Bian, Z. Liu, *Green Chem.* **2024**, *26*, 2540.
